# Secretory CAZymes profile and GH19 enzymes analysis of *Corallococcus silvisoli* c25j21

**DOI:** 10.3389/fmicb.2024.1324153

**Published:** 2024-02-05

**Authors:** Xiaoli Zhou, Xianmin Zhou, Xianjiao Zhang, Honghong Dong, Yijie Dong, Honghui Zhu

**Affiliations:** Key Laboratory of Agricultural Microbiomics and Precision Application (MARA), Key Laboratory of Agricultural Microbiome (MARA), State Key Laboratory of Applied Microbiology Southern China, Guangdong Provincial Key Laboratory of Microbial Culture Collection and Application, Institute of Microbiology, Guangdong Academy of Sciences, Guangzhou, China

**Keywords:** GH19, myxobacteria, CAZyme, lysozyme, secretory proteome

## Abstract

Extracellular enzymes play important roles in myxobacteria degrading macromolecules and preying on other microorganisms. Glycoside hydrolases 19 (GH19) are widely present in myxobacteria, but their evolution and biological functions have not been fully elucidated. Here we investigated the comparative secretory proteome of *Corallococcus silvisoli* c25j21 in the presence of cellulose and chitin. A total of 313 proteins were detected, including 16 carbohydrate-active enzymes (CAZymes), 7 of which were induced by cellulose or chitin, such as GH6, GH13, GH19, AA4, and CBM56. We further analyzed the sequence and structural characteristics of its three GH19 enzymes to understand their potential functions. The results revealed that myxobacterial GH19 enzymes are evolutionarily divided into two clades with different appended modules, and their different amino acid compositions in the substrate binding pockets lead to the differences in molecular surface electrostatic potentials, which may, in turn, affect their substrate selectivity and biological functions. Our study is helpful for further understanding the biological functions and catalytic mechanisms of myxobacterial CAZymes.

## 1 Introduction

Myxobacteria are a class of bacteria with the ability to degrade biomass and prey on a variety of other microorganisms (Munoz-Dorado et al., 2016). They have good application potential and research value in the fields of biomass waste utilization, pathogen biocontrol, and drug development. Extracellular enzymes have long been considered to be important factors in macromolecular degradation and microbial predation by myxobacteria (Sudo and Dworkin, 1972; Berleman and Kirby, 2009; Konovalova et al., 2010), but the specific enzyme functions and mechanisms remain to be clarified.

In recent years, several extracellular carbohydrate-active enzymes (CAZymes) have been characterized as macromolecular degradation activities. In terms of macromolecular degradation, there are several research reports on amylase, such as AmyM, CoMA, and IsoM from *Corallococcus* sp. EGB, which have been well characterized (Li et al., 2015, 2017; Zhou et al., 2018; Chen X. et al., 2020). Fewer studies on myxobacterial cellulose-active enzymes have been reported. Two enzymes from *Myxobacter* sp. AL-1, Cel9 and chitosanase-cellulase, were biochemically characterized as endoglucanases and bifunctional chitosanase-cellulase, respectively (Pedraza-Reyes and Gutierrez-Corona, 1997; Tellez-Valencia et al., 2003). We have previously characterized two lytic polysaccharide monooxygenases ViLPMO10A and ViLPMO10B from *Vitiosangium* sp. GDMCC 1.1324 and found that they have chitin- and cellulose- activities, respectively. The synergistic activity of ViLPMO10B and cellulase for straw degradation was also investigated (Zhou et al., 2020).

CAZymes have also been found to participate in the process of myxobacteria preying on other microorganisms. The outer membrane β-1,6-glucanase GluM was found to be essential for EGB to prey on phytopathogenic fungi *Magnaporthe oryzae* due to its ability to target and degrade fungal cell wall glucans (Li et al., 2019b). A secreted cocktail of β-1,3-glucanases was confirmed to be a major contributor of *Archangium* sp. AC19 during predation on oomycetes *Phytophthora* (Zhang et al., 2023). Besides, an endochitinase CcCti1 belonging to the glycoside hydrolase 18 (GH18) family was characterized to show biocontrol activity against the plant pathogen fungus *M. oryzae in vitro* (Li et al., 2019a). The cell wall composition of bacteria and fungi is different, and the myxobacterial enzymes that target them may also be different. As for CAZymes involved in the process of myxobacteria preying on bacteria, the GH19 family enzymes have been reported. LlpM from *M. xanthus* has been characterized as a lysozyme and has lytic activity against Gram-positive bacteria e.g., *Micrococcus luteus* and *Bacillus subtilis* (Arend et al., 2021). We previously identified lysozyme C25GH19B from *C. silvisoli* c25j21, which showed bacteriolytic activity against both Gram-positive and Gram-negative pathogenic bacteria (Li et al., 2022). The GH19 family contains enzymes that are biochemically characterized as chitinases and lysozymes (or endolysins). Among them, chitinases come mainly from plants, and a few from *Streptomyces*, and lysozymes mainly come from phages. GH19 enzyme genes are widespread in myxobacteria, and some strains have more than one GH19 gene, such as *C. silvisoli* c25j21, which has three redundant GH19 genes in its genome. However, apart from the two enzymes mentioned above, little is known about the GH19 enzymes derived from myxobacteria.

In this study, to understand the biological functions of myxobacterial CAZymes, especially the GH19 enzymes, we performed a comparative secretory proteomic analysis of *C. silvisoli* c25j21 in the presence of cellulose and chitin. In addition, we investigated the sequence and structural characteristics of its three GH19 enzymes and their myxobacterial homologous proteins. Our study will provide the basis for further understanding and research on the biological functions and enzymatic mechanisms of myxobacterial CAZymes.

## 2 Materials and methods

### 2.1 Culture of *C. silvisoli* c25j21 and protein preparation

Three culture media were used to study the secretory proteome of *C. silvisoli* c25j21. For Blank group, MD1 liquid medium (Casein peptone 0.6%, soluble starch 0.2%, MgSO_4_⋅7H_2_O 0.2%, CaCl_2_⋅2H_2_O 0.04%, pH 7.2) was used. For the Cel and Chi groups, 1% (w/v) rice straw powder or α-chitin was added to the MD1 medium containing half of the casein peptone and half of the soluble starch, respectively. The swarms of c25j21 grown on VY/2 agar were taken and inoculated into the above medium and cultured at 30°C with shaking at 180 rpm for 1 week. Each group had three replicates. After the culture, the culture supernatant was separated by centrifugation at 12,000 *g* and frozen in liquid nitrogen.

Protein preparation and proteomic analysis were performed by Shanghai Applied Protein Technology Co., Ltd., China. SDT (4% SDS, 100 mM Tris-HCl, 1 mM DTT, pH 7.6) buffer was used for protein extraction. The amount of protein was quantified with the BCA Protein Assay Kit (Bio-Rad, USA). Protein was digested by trypsin and the peptides of each sample were desalted, concentrated, and reconstituted in 0.1% (v/v) formic acid.

### 2.2 LC-MS/MS and data analysis

LC-MS/MS analysis was performed on a Q exactive mass spectrometer (Thermo Scientific) that was coupled to Easy nLC (Proxeon Biosystems, now Thermo Fisher Scientific, Inc., Waltham, USA). The peptides were loaded onto a reverse phase trap column (Thermo Scientific Acclaim PepMap100, 100 μm × 2 cm, nanoViper C18) connected to the C18-reversed phase analytical column (Thermo Scientific Easy Column, 10 cm long, 75 μm inner diameter, 3 μm resin) in buffer A (0.1% Formic acid) and separated with a linear gradient of buffer B (84% acetonitrile and 0.1% Formic acid) at a flow rate of 300 nl/min. The mass spectrometer was operated in positive ion mode. The MS raw data for each sample were combined and searched using the MaxQuant 1.5.3.17 software for identification and quantitation analysis.

### 2.3 Annotation of CAZymes

The protein sequences of c25j21 were submitted to the dbCAN3 webserver^1^ and annotated using HMMER (Zheng et al., 2023).

### 2.4 Multiple sequence alignment and phylogenetic tree construction

The protein sequences of C25GH19A (WP_143898266) and C25GH19B (WP_161664743) were used as queries to blast against the NCBI non-redundant protein sequences database through the blastp algorithm. The max target sequences and the expected threshold were set as 100 and 0.05, respectively. The obtained amino acid sequences were conducted with multiple sequence alignment using the Clustal Omega web server^2^ (Sievers and Higgins, 2018) (Supplementary Table 1). The results were rendered by ESPript 3.0 (Robert and Gouet, 2014). The neighbor-joining phylogenetic trees were created by MEGA-X (Kumar et al., 2018), and the figure was generated by the iTOL web server^3^ (Letunic and Bork, 2019). The domain compositions of the proteins were predicted by the NCBI batch CD-search tool (Lu et al., 2020). The amino acid sequences were searched against Pfam database with an expected value threshold of 0.01 (Mistry et al., 2021). The integrated figures of the phylogenetic trees and the conserved domains were generated by TBtools (Chen C. et al., 2020).

### 2.5 Homology modeling

The homology model structures of the proteins were created by the SWISS-MODEL web server^4^ (Waterhouse et al., 2018), and the templates are listed in Table 1. Verify_3D (Luthy et al., 1992) was used to check the residue profiles of the 3D models obtained. PROCHECK (Laskowski et al., 1996) analysis was performed to assess the stereochemical qualities of the 3D models. PyMOL software (The PyMOL Molecular Graphics System, Version 1.8 Schrödinger, LLC, De Lano Scientific, San Carlos, CA, USA) was used to view the structures and generate figures.

**TABLE 1:** Templates for homology modeling in this study.

Proteins (NCBI entry)	Templates (PDB or AlphaFold entry)
WP_239013920	AF-A0A1L9B4N1-F1
WP_143898266	AF-A0A3A8TK71-F1
WP_147439293	4OK7
WP_163999114	AF-A0A1H7SNC6-F1
MCA2980842	AF-A0A848L6Y9-F1
WP_161664743	4OK7
NBD12723	AF-A0A1H7SNC6-F1

### 2.6 Protein sequence conservation analysis

The multiple sequence alignment files of the proteins in Clade I and Clade II were submitted to the AL2CO online server (Pei and Grishin, 2001), respectively, and residue conservation analysis were performed using the default parameters. The results were mapped onto the model structures of C25GH19A and C25GH19B using PyMOL, respectively.

### 2.7 Substrate binding pocket prediction

The substrate binding pockets of C25GH19A and C25GH19B, without any ligand, were calculated by the CavityPlus web server with default parameters^5^ (Wang et al., 2023).

### 2.8 Electrostatic potential and pKa value calculation

Electrostatic potentials of the protein surfaces were calculated using the program APBS (Jurrus et al., 2018). The PQR file and the pKa values were calculated via PDB2PQR specifying the pH at 7 (Dolinsky et al., 2007). The figures were generated by PyMOL software.

### 2.9 Molecular docking

The peptidoglycan disaccharide substrate NAGNAM (N2) was extracted from the complex structure of PGRP-IBETAC (PDB entry: 2EAX) (Cho et al., 2007). The chitin trisaccharide substrate (N3) was extracted from the complex structure of OfChtIII (PDB entry: 5WV9) (Liu et al., 2018). AutoDock 4.2.6 (The Scripps Research Institute, La Jolla, CA, USA) was used for the molecular docking study (Morris et al., 2009). The AutoDock Tools 1.5.6 was used to prepare the protein and ligands for the docking procedure. Kollman charges and polar hydrogens were added. AutoGrid was used to generate the grid maps. Each grid was centered at the substrate binding pockets of the enzymes. The grid dimensions were 126 points in each dimension separated by 0.375 Å. The files were generated in PDBQT format. For ligands, random starting positions and orientations were used. The genetic algorithm was used with 2,500,000 energy evaluations and a population of 150 individuals, and 50 runs were carried out.

## 3 Results

### 3.1 The comparative secretory proteome of *C. silvisoli* c25j21 in the presence of cellulose and chitin

**FIGURE 1:**
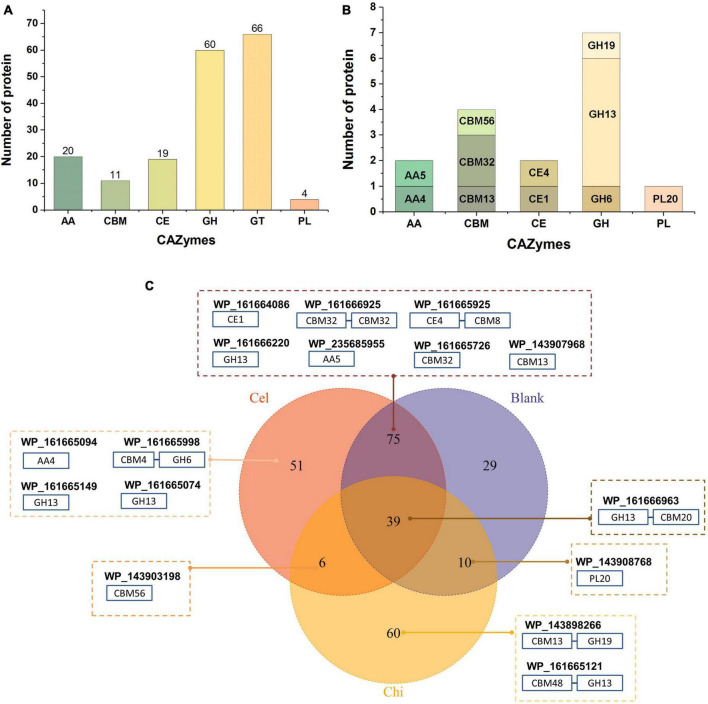
Secretory proteomic analysis of the CAZymes of c25j21. **(A)** CAZymes encoded by the genome of c25j21 annotated by dbCAN. **(B)** CAZymes detected by secretory proteomic analysis. **(C)** Venn plots of secreted proteins under different growth conditions. Numbers indicate the amount of protein detected. Cel, medium added cellulose; Chi, medium added α-chitin; Blank, medium without cellulose and chitin. In the dashed boxes are the NCBI IDs of the annotated CAZymes along with the schematic representation of their domain compositions.

We previously isolated a myxobacterium strain, *C. silvisoli* c25j21 (Zhang et al., 2022), whose genome encodes 7197 proteins, 180 of which are annotated as CAZymes, including 20 auxiliary activities (AA), 11 separate carbohydrate-binding modules (CBM), 19 carbohydrate esterases (CE), 60 glycoside hydrolases (GH), 66 glycosyltransferases (GT), and 4 polysaccharide lyases (PL) (Figure 1A). To understand the roles of these CAZymes in polysaccharides metabolism, we investigated the secretory proteome of c25j21 when grown in the presence of cellulose or chitin, or neither, respectively. A total of 313 proteins were detected, of which 16 CAZymes were identified, including 2 AAs, 4 individual CBMs, 2 CEs, 7 GHs, and 1 PL (Figure 1B and Supplementary Tables 2, 3). There were 153 constitutively expressed proteins, including 9 CAZymes, such as GH13, PL20, CE1, CE4, AA5, CBM32, CBM13, etc (Figure 1C). Cellulose induced the expression of 57 proteins, including 5 CAZymes, namely GH13, GH6, AA4, CBM56, etc. Chitin induced the expression of 66 proteins, three of which were CAZymes, including GH13, GH19, and CBM56.

Consistent with previous studies showing that c25j21 has good starch utilization ability, in this study, it was found that this strain can secrete a variety of amylases, including the members of the subfamily GH13_6, GH13_10, GH13_11, and GH13_13. Interestingly, although c25j21 did not show cellulose and chitin hydrolytic activities in our previous plate experiments, the addition of lignocellulose or α-chitin to MD1 medium with reduced carbon sources could induce the secretion of related enzymes in the present study. Rice straw induced the secretion of two enzymes belonging to the lignin-active AA4 and cellulose-active GH6 family, respectively. At the same time, α-chitin induced the secretion of a GH19 family enzyme, presumably with chitinase activity. Both of these added substrates induced the secretion of a CBM56 family protein, which may assist in polysaccharide degradation. In general, c25j21 has a large number of CAZyme genes, but relatively few of them are secreted. These results suggest that not many of the CAZymes encoded in the genome are involved in polysaccharide metabolism, and they may have other functions.

### 3.2 Three distinct GH19 enzymes from *C. silvisoli* c25j21

**FIGURE 2:**
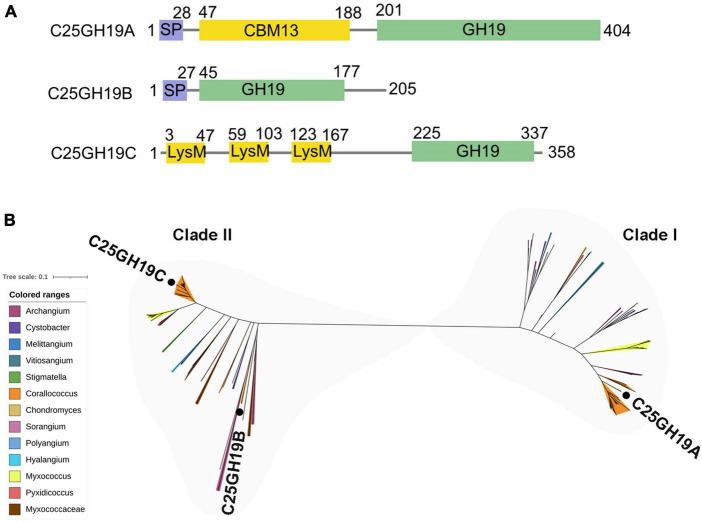
Protein sequences analysis of C25GH19s. **(A)** Module composition of C25GH19A, C25GH19B, and C25GH19C. **(B)** Phylogenetic tree of the GH19 modules of C25GH19s and their homologous proteins. Proteins from different myxobacteria genera are represented by different colors. C25GH19A, C25GH19B, and C25GH19C are labeled. The phylogenetic tree was created by MEGA-X, and the figure was generated by iTOL.

There are three GH19 enzymes encoded in the genome of c25j21, designated as C25GH19A (WP_143898266), C25GH19B (WP_161664743), and C25GH19C (NBD12723) (Figure 2A), of which only C25GH19A is induced by chitin, suggesting that they may have different biological functions. In our previous work, we have recombinantly expressed the C25GH19B in *E. coli* and characterized its enzymatic activity. C25GH19B was shown to be a lysozyme with bacteriolytic activity against both Gram-positive and Gram-negative plant pathogenic bacteria (Li et al., 2022). Both C25GH19A and C25GH19B have an N-terminal signal peptide, while C25GH19C without a signal peptide is predicted to be secreted by a non-classical pathway, indicating that they are extracellular enzymes. Unlike C25GH19B, which has only the catalytic GH19 module, both C25GH19A and C26GH19C have appended modules. A carbohydrate-binding module family 13 (CBM13) and lysin motif (LysM) modules located at the N-terminal of C25GH19A and C25GH19C, respectively, may play an important role in their substrate binding.

Multiple sequence alignment of the GH19 modules of the three enzymes revealed a 66% sequence identity between C25GH19B and C25GH19C, whereas C25GH19A shared less homology with them, with only 37 to 38% sequence identity. Comparing the protein sequences of these three GH19 modules against NCBI-nr protein database showed that their homologous proteins were widely distributed in several genera of myxobacteria (Figure 2B). Phylogenetic analysis revealed that these proteins clustered into two clades, with C25GH19B and C25GH19C in the same clade (clade II) and C25GH19A in the other clade (clade I). These results suggest that the two types of GH19 modules may have evolved different catalytic functions.

### 3.3 Phylogeny of myxobacterial GH19 enzymes

**FIGURE 3:**
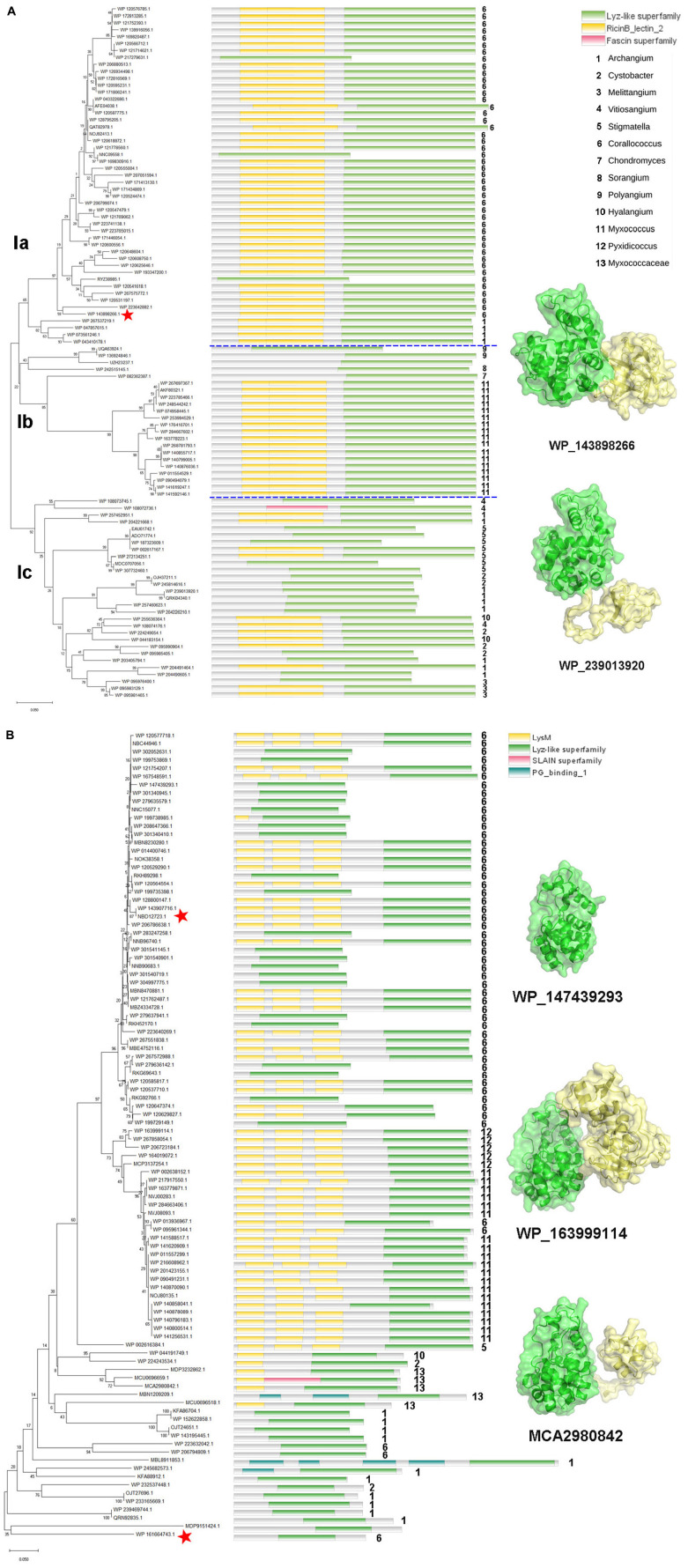
Phylogeny and module composition of the proteins in clade I **(A)** and clade II **(B)**. The tree was generated using the GH19 modules only. Appended modules that are present in the full protein sequence are indicated in the conserved module composition column but were not included in the calculation of the tree. Model structures of the representative members with different module compositions are mapped onto the tree, with colors representing the different modules. C25GH19s are indicated by red stars.

The enzymes of clade I are derived from the orders *Myxococcales* and *Polyangiales*. Phylogenetic analysis of GH19 modules revealed that these members clustered into three groups (Figure 3A). Group Ia includes enzymes derived from the genera *Corallococcus* and *Archangium*. All members of this group have a CBM13 module, except for three proteins with incomplete sequences. In addition to the genus *Myxococcus*, group Ib also includes enzymes derived from the genera *Polyangium*, *Sorangium*, and *Chondromyces*, belonging to the order *Polyangiale*, whose additional modules could not be annotated. The enzymes in the group Ic are derived from six genera of myxobacteria in the *Myxococcaceae* family, namely *Vitiosangium*, *Archangium*, *Stigmatella*, *Cystobacter*, *Hyalangium*, and *Melittangium*, and more than half of them have an appended module of unknown function.

Unlike clade I, the members of clade II are all derived from the order *Myxococcales*, covering seven genera, e.g., *Corallococcus*, *Pyxidicoccus*, *Myxococcus*, *Stigmatella*, *Hyalangium*, *Cystobacter*, and *Archangium* (Figure 3B). In the phylogenetic tree, the members of the basal branches near the root position mostly contain no additional modules, while the members of the terminal branches mostly have one to three LysM modules. It is speculated that they may have acquired appended modules through gene transfer and duplication during evolution.

More than 75% of clade I members have CBM13 modules, which are also known as Ricin-like lectin modules, were first identified in plant lectins such as ricin, and have been found in a number of glycoside hydrolases and glycosyltransferases, which function in binding to a variety of polysaccharides. About 60% of the clade II members have LysM modules, which are also known as CBM50. LysM was initially discovered in the lysozyme of a bacteriophage as a peptidoglycan binding module. Later, it was gradually found that LysM modules were widely present in plants and pathogenic fungi, and regulated plant immune response by binding to chitin in the cell wall of pathogenic fungi. The different composition of additional modules among the myxobacterial GH19 enzymes suggests that they may have different substrate selectivity.

### 3.4 Structural characteristics of the two types of GH19 enzymes

**FIGURE 4:**
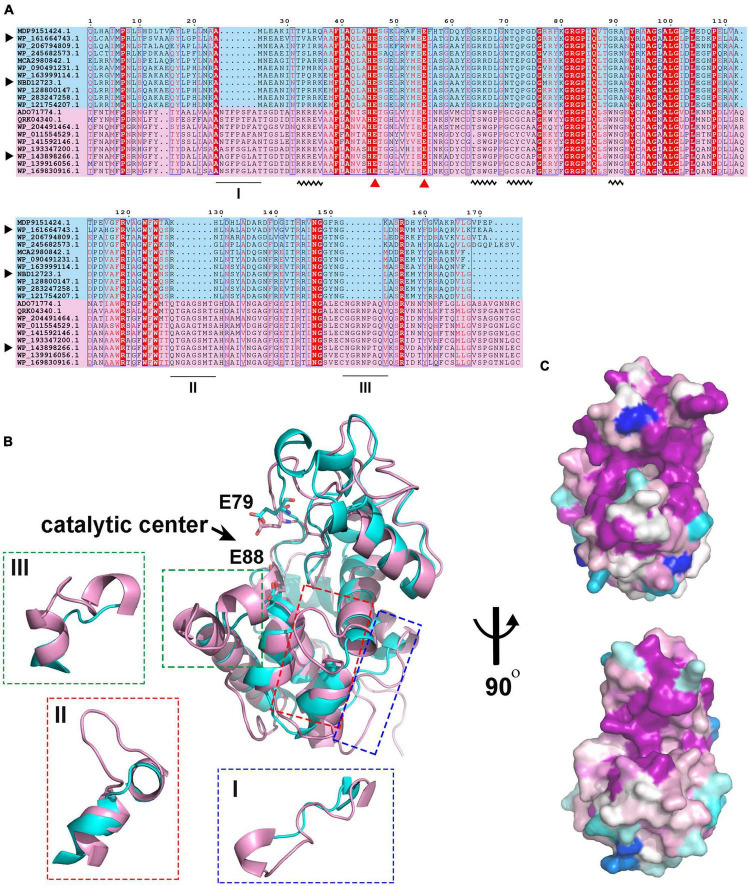
Sequence and structure alignment of C25GH19s. **(A)** Multiple sequence alignment of C25GH19s and their homologs. The pink and blue background indicate the proteins in Clade I and Clade II. The catalytic residues are indicated by red triangles. The black triangle indicates C25GH19A (WP_143898266), C25GH19B (WP_161664743), and C25GH19C (NBD12723). The black underlines indicate loops of significantly different lengths. The black wavy lines indicate conserved sites that differ significantly between the two clades. **(B)** Superposition of the model structures of C25GH19A (magenta) and C25GH19B (cyan). The catalytic residues are shown in sticks and labeled according to C25GH19B. The three loops of remarkably different lengths are circled and shown in the enlarged diagrams. **(C)** Conservation of residues across the Clade I (up) and Clade II (down) calculated by AL2CO and mapped onto C25GH19A and C25GH19B, respectively. Colors range from variable (blue) to conserved (purple).

To understand the differences in amino acid composition and structural characteristics between the enzymes of the two clades, we performed multiple sequence alignment (MSA) and structural alignment of them. MSA showed that the catalytic center residues of the two clade enzymes were conserved (Figure 4A). One significant difference between them is that the enzymes in clade I have three longer loops compared to those in clade II. Among them, loops II and III are spatially located near the catalytic center and may influence the shape of the substrate binding pockets and participate in substrate binding (Figure 4B). Meanwhile, there are some conserved sites within the two clades, but these sites are significantly different between the two clades (Figure 4A, wavy lines). These sites contain more positively charged amino acids such as Arg and Lys in members of clade II compared to clade I. Amino acid conservation analysis of enzymes in both clades was performed separately, and the results were mapped to the 3D model structures of C25GH19A and C25GH19B, respectively (Figure 4C). The results showed that the most conserved sites of enzymes in both clades are located near the catalytic center, at the domain interface, that is, the substrate-binding pockets. These results suggest that the two types of GH19 enzymes may have different substrate recognition modes and catalytic functions.

**FIGURE 5:**
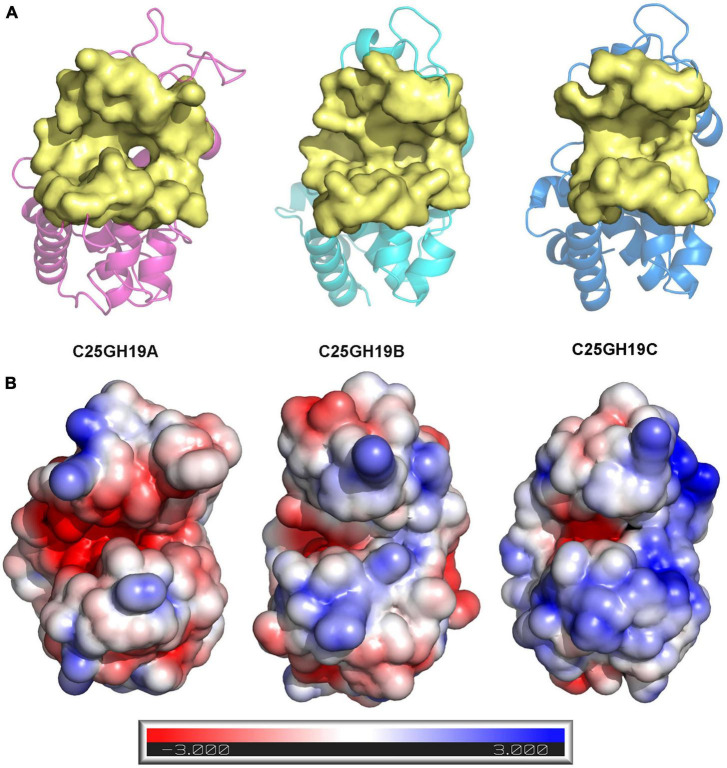
Structural comparison between C25GH19s. **(A)** The substrate binding pockets (yellow) of C25GH19A (magenta), C25GH19B (cyan), and C25GH19C (blue) predicted by CavityPlus. **(B)** The electrostatic potentials of protein surfaces. The electrostatic potential ranges from –3 to +3. Red indicates negatively charged, blue indicates positively charged, and white indicates uncharged.

Since these significant differences in amino acid composition are located in the substrate binding pocket region, to further understand the impact of these differences on enzyme structures as well as catalytic functions, we analyzed the substrate binding pockets of the three C25GH19s (Figure 5A). The results showed that the substrate binding pockets of all three enzymes are relatively wide open regions located at domain interfaces, which are suitable for binding of large polysaccharide substrates. The protein surface electrostatic potentials of these enzymes at pH 7.0 were calculated. As shown in Figure 5B, the substrate binding pocket of C25GH19A is negatively charged, which is favorable for enzymes binding to polysaccharides with positive charge, such as chitin. The substrate binding pockets of C25GH19B and C25GH19C are positively charged, which may be more suitable for the binding of negatively charged polysaccharides, such as peptidoglycan. This is consistent with our previous findings, that is, C25GH19B has peptidoglycan hydrolysis activity but cannot hydrolyze chitin (Li et al., 2022).

### 3.5 Substrate binding modes of C25GH19s

**FIGURE 6:**
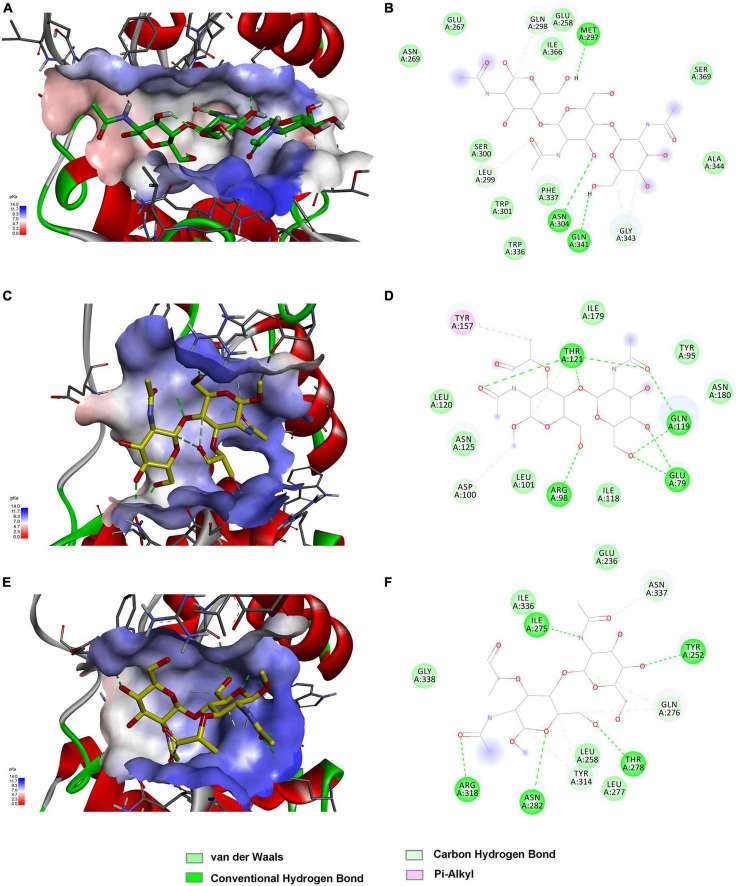
Substrate binding modes of C25GH19s predicted by molecular docking. **(A)** C25GH19A-chitin trisaccharide (GlcNAc)_3_ complex. **(B)** The interactions between (GlcNAc)_3_ and the residues of C25GH19A. **(C)** C25GH19B-NAGNAM complex. **(D)** The interactions between NAGNAM and the residues of C25GH19B. **(E)** C25GH19C-NAGNAM complex. **(F)** The interactions between NAGNAM and the residues of C25GH19C.

The above findings inspired us to further explore the binding modes of these enzymes to different polysaccharide substrates. To analyze the possible interaction of C25GH19A with chitin substrates, the chitin trisaccharide (GlcNAc)_3_ was extracted from the oligosaccharide-complexed crystal structure of the chitinase OfChtI and docked into the model structure of C25GH19A (Figures 6A, B). The chitin trisaccharide binds well in the substrate binding pocket of C25GH19A with a binding free energy of −2.32 kcal/mol. The residues M297, N304, Q341, Q298, L299, and G343 form hydrogen bonds with (GlcNAc)_3_, while residues N269, E267, E258, I366, S369, A344, S300, W301, W336, and F337 interact with the chitin trisaccharide through van der Waals. Disaccharide NAGNAM was extracted from the complex crystal structure of the protein PGRP-Iβ and docked into the model structures of C25GH19B and C25GH19C, respectively, to analyze their potential binding modes to peptidoglycan substrates (Figures 6C–F). The binding free energies of C25GH19B or C25GH19C complexes with NAGNAM obtained by molecular docking were −5.64 and −5.81 kcal/mol, respectively. In C25GH19B, residues T121, Q119, E79, R98, and D100 form hydrogen bonds with the substrate NAGNAM; residues I179, Y95, N180, I118, L101, N125, and L120 participate in van der Waals interactions; in addition, Y157 forms Pi-alky interaction with NAGNAM (Figure 6D). C25GH19C also interacts with NAGNAM via hydrogen bonds and van der Waals interactions, and the residues involved include I275, Y252, T278, N282, R318, N337, Q276, Y314, E236, L277, L258, G338, and I336 (Figure 6F). Since both chitin and peptidoglycan are natural polymers, the use of disaccharides and trisaccharides as model substrates here only indicates the possible binding modes between them, and further studies on the activities and intermolecular interactions of these enzymes on these natural substrates are still needed in future work.

## 4 Discussion

Myxobacteria generally have larger genomes than other bacteria, and the large number of extracellular enzymes encoded by them are considered to be major contributors to their powerful biomass degradation ability and important weapons for preying on other microorganisms. In this study, we performed a comparative secretory proteomic analysis of a previously isolated myxobacterium *C. silvisoli* c25j21. The results showed that the number of CAZymes induced by chitin and cellulose was not large. On the one hand, we found that a considerable number of secreted proteins induced by chitin and cellulose could not be annotated and predicted, and there may be some novel CAZymes that need more biochemical experiments to explore. On the other hand, these results also suggested that most CAZymes of c25j21 may have other biological functions than biomass degradation. As with its three redundant GH19 enzymes, only C25GH19A can be induced by chitin, while C25GH19B, which is not induced by chitin, has previously been characterized by lysozyme activity.

Sequence and structural analysis of these GH19enzymes and their homologous proteins from myxobacteria showed that these proteins have evolved into two clades, in which clade I, where C25GH19A is located, is distributed in the two families of *Myxococcaceae* and *Polyangiaceae*, while clade II, where C25GH19B and C25GH19C are located, is distributed only in the family of *Myxococcaceae*. This difference in the distribution of these enzymes among myxobacteria species may be related to their biological functions.

The substrate binding pocket is the most important region affecting substrate recognition, binding, and catalysis of enzymes. From the multiple sequence alignments and structural comparisons, it can be seen that the amino acid composition of this region is the most conserved within each clade, but the difference between the two clades is significant. Although both clades of GH19s have wide, open substrate binding pockets suitable for binding large polysaccharide substrates, their substrate binding pockets have different molecular surface electrostatic potentials, which makes them suitable for the accommodation of polysaccharides with different properties, such as positively charged chitin or negatively charged peptidoglycan.

Most CAZymes are multi-module enzymes, and in addition to the catalytic modules, appended modules generally have substrate binding ability, thus affecting the substrate selectivity of the enzymes. The appended modules of these two clades of myxobacterial GH19s in this study are also distinct. More than 75% of the enzymes of clade I carry the CBM13 module, while about 60% of the enzymes of clade II carry one or more LysM modules. Meanwhile, phylogenetic analysis showed that these enzymes may have acquired appended modules through gene transfer and duplication during evolution. These results suggest that although the myxobacteria, such as c25j21, have redundant GH19 genes, they have evolved different enzyme properties and may have non-redundant biological functions.

In summary, this study provides a better understanding of the secreted proteome of *C. silvisoli* c25j21 in the presence of cellulose and chitin, as well as its three redundant GH19 enzymes. These results will lay a theoretical and experimental foundation for further study of the physiological functions of CAZymes in the process of myxobacteria predation and polysaccharides metabolism, as well as their development and application.

## Data availability statement

The original contributions presented in this study are included in this article/Supplementary materials, further inquiries can be directed to the corresponding author.

## Author contributions

XiaolZ: Conceptualization, Funding acquisition, Investigation, Methodology, Writing – original draft, Writing – review & editing. XianmZ: Writing – review & editing. XianjZ: Writing – review & editing. HD: Writing – review & editing. YD: Writing – review & editing. HZ: Funding acquisition, Project administration, Writing – review & editing.

## Footnotes

1
https://bcb.unl.edu/dbcan2/

2
https://www.ebi.ac.uk/tools/msa/clustalo/

3
https://itol.embl.de/

4
https://swissmodel.expasy.org/

5
http://pkumdl.cn:8000/cavityplus/

## Supplementary material

Supplementary Table 1 Protein sequences in this study.

Supplementary Table 2 Data for the venn plots of secreted proteins under different growth conditions.

Supplementary Table 3 The secretory proteome sequencing data.
