# Supplementary material for: Secretory CAZymes profile and GH19 enzymes analysis of Corallococcus silvisoli c25j21
Source: Front Microbiol. 2024 Feb 5;15:1324153. doi: 10.3389/fmicb.2024.1324153 (PMC10875029; doi:10.3389/fmicb.2024.1324153)
Supplement: Supplementary Table 1 — Protein sequences in this study. [file Table_1.DOCX]

Supplementary Material

# Supplementary Data

Protein sequences in this study

>WP_161664743.1 glycoside hydrolase family 19 protein [Corallococcus silvisoli]

MPGSGVLLGGAALVGLGAWAMRGGAAAAPLTVDQLCAVMPRLTPSVAASYLGPLLAAMREAEVTTVARVAAFLAQLAHESGELRYWEELATGDAYEGRKDLGNTQPGDGRRYKGRGPIQLTGRANYRAAGAALGLPLEDKPELAALPAHGFRVAGWYWQSRHLNALADVADFVGVTRAINGGTNGLDNRVMYFDRAQRVLKTEAA

>WP_239469744.1 glycoside hydrolase family 19 protein [Archangium violaceum]

MSSNRWALAAAGAAILGGLVYLATRRTSRFAVQLRSIMPRLSLVDAGRYAGELLAAMDEGGITTPARQAAFLAQLAHESGELRYMEELASGEAYEGRKDLGNTQTGDGRRYKGRGPIQLTGRSNYRAAGDALGLPLEQQPELAAQPDVGFRVAAWFWRSRELSALADAGNFREVTRRINGGYNGLDQREAYWSRARQVLGA

>QRN92835.1 glycoside hydrolase family 19 protein [Archangium violaceum]

MRCTRSCGGRTVCCRASRPSRSAARGTCASWCGCAGACGAWSRRAVRAMSSNRWALAAAGAAILGGLVYLATRRTSRFAVQLRSIMPRLSLVDAGRYAGELLAAMDEGGITTPARQAAFLAQLAHESGELRYMEELASGEAYEGRKDLGNTQTGDGRRYKGRGPIQLTGRSNYRAAGDALGLPLEQQPELAAQPDVGFRVAAWFWRSRELSALADAGNFREVTRRINGGYNGLDQREAYWSRARQVLGA

>MBN1209209.1 peptidoglycan-binding protein [Myxococcaceae bacterium]

MAAGTPPPPPSPVGVAQPWRPAPSWDAVLFQGQFLEQGHQGPVVAELQKLLGVSSDGQFGAQTRAAVEAFQRAVHISPSSEAVGRVGKTTLRTLLTLKRHWLPAPSLEEIRKGSRLLGVGQAGPAVKEVQRLLQLAPAQQDGFFGAGTQAAVVDFQRKAGLPQVPGSEGVIGQALLEALLRRQGQAAGTGAGVTLQQLRAIMPRLSEAQASSYLPHLNAAMAQVEITTPRRQAAFLAQLAHESGELRFWEELADGKAYEGRRDLGNTQPGDGPRYKGRGPIQLTGRANYRAAGQALGIDLEKSPERARDPDVGFRVAAWYWQSRGLNALADAGDFREITRRINGGYNGLPQREEYYARALRIL

>MCU0696659.1 LysM peptidoglycan-binding domain-containing protein [Myxococcaceae bacterium]

MSTYQIRRGDTLSALASRFGTSVSALARLNNIKNPDLIYAGATLRLPDSYGSARPSAPGAVPQPRPSQPGASRPQGVDEVQPPSGDTNGITADELRRVMPNLSPELAQRYLPYLNSAMNEAGITTPARRSAFLAQLAHESGELRYMEEIASGAAYEGRRDLGNTQPGDGRRYKGRGPIQLTGRANYRAAGEALGIDLERNPERAADPDVAFRIATWYWQSRGLNGLADQGNFREITRRINGGYNGMADRQRYYERAQQVF

>KFA86704.1 hypothetical protein Q664_52795 [Archangium violaceum Cb vi76]

MQQAGPISLEQLKAIMPRVPVAKASEYLGHLNAAMREREITSPKRAAAFLAQLAHESGELRYMEEIASGAAYEGRADLGNTQPGDGKRYKGRGPIQLTGRANYRTAGKALGVALEDNPTRAAEPDVAFRVAAWYWGSRGLNSLADTGNFREITRLINGGYNGMADREAFWAKAKATLGAA

>WP_147439293.1 glycoside hydrolase family 19 protein, partial [Corallococcus sp. CA049B]

GVTVAQLRRIMPNLSQAKAEQYLPHLNKAMAEASINTPRRKEMFLAQLAHESGELRYMEEIASGAAYEGRKDLGNTQPGDGKRYKGRGPIQLTGRANYRAAGKALGIDLEGHPERAKDPDVAFRIAGWYWQSRNLNSYADAGNFREVTRRINGGYNGLSSREMYYRRAQDVLG

>WP_199753869.1 MULTISPECIES: glycoside hydrolase family 19 protein, partial [Corallococcus]

SSGKGGVTVAQLRRIMPNLSQAKAEQYLPHLNKAMAEANINTPRRKEMFLAQLAHESGELRYMEEIASGAAYEGRKDLGNTQPGDGKRYKGRGPIQLTGRANYRAAGKALGIDLEGHPERAKDPDVAFRIAGWYWQSRNLNSYADAGNFREVTRRINGGYNGLASREMYYRRAQDVLG

>WP_223632042.1 glycoside hydrolase family 19 protein [Corallococcus sp. EGB]

MILEALGLTAVAAAGAPKVVNKVRPKVDLVAGLTLAQLRAIMPNLAEAKAVEYLPLLAKAMNEAGINTPLRRAGFLAQLAHESGEFRWMEEIADGTAYEGRKDLGNTQPGDGARYKGRGPIQLTGRANYRAAGAALGLDLEGNPDQAKTPAVGFRVASWYWTSKSINPLADARDLVGMTKKVNGGTNGLEDRKKYYERALRVLGVAA

>WP_301340945.1 MULTISPECIES: glycoside hydrolase family 19 protein, partial [Corallococcus]

GKGGVTVAQLRRIMPNLSQAKAEQYLPHLNKAMAEANINTPRRKEMFLAQLAHESGELRYMEEIASGAAYEGRKDLGNTQPGDGKRYKGRGPIQLTGRANYRAAGKALGIDLEGHPERAKDPDVAFRIAGWYWQSRNLNSYADAGNFREVTRRINGGYNGLASREMYYRRAQDVLG

>MDP3232862.1 glycoside hydrolase family 19 protein [Myxococcales bacterium]

MRDYQIRRGDTLSQLAARFGTSVSALARLNNIRNPDLIIAGANLRVPDGFSAPRPSAPGSVPSTQPSAPSTRPDRTEDVAPAQGDTRGISAEELQRIMPSLSPQLAQRYLPYLNSAMNEAGITTPARRAAFLAQLGHESGGLRYFEEIASGAAYEGRRDLGNTQPGDGRRFKGRGPIQLTGRANYRAAGQALGIDLERNPERAADPDVGFRIAAWFWQSRGLNSLADQGNFREITRRINGGYNGYDDRVQYWNRARQVL

>WP_152622858.1 glycoside hydrolase family 19 protein [Archangium violaceum]

MPRNVGFILLAAAVVAVGAYVLMQQAGPISLEQLKAIMPRVPVAKASEYLGHLNAAMREREITSPKRAAAFLAQLAHESGELRYMEEIASGAAYEGRADLGNTQPGDGKRYKGRGPIQLTGRANYRTAGKALGVALEDNPTRAAEPDVAFRVAAWYWGSRGLNSLADTGNFREITRLINGGYNGMADREAFWAKAKATLGAA

>WP_199735398.1 glycoside hydrolase family 19 protein, partial [Corallococcus sp. AB045]

GTPVTSGGKGGVTVAQLRRIMPNLSQAKAEQYLPHLNKAMAEANINTPRRKEMFLAQLAHESGELRYMEEIASGAAYEGRKDLGNTQPGDGKRYKGRGPIQLTGRANYRAAGKALGIDLEGNPERAKDPDVAFRIAGWYWQSRNLNSYADAGNFREVTRRINGGYNGLASREMYYRRAQDVLG

>MCA2980842.1 LysM peptidoglycan-binding domain-containing protein [Myxococcaceae bacterium]

MRSYLIRPGDTLSQLAARFGTSVSALAKLNDIRNPDLIYAGATLRLPDSYASPRPSAPGAVNEPSPTRALPSRPEGVDPVEAPRGDTSGISVDELRRVMPNLSPELAQRYLPYLNAAMNEAGITTPARRSAFLAQLAHESGELRYMEEIASGADYEGRRDLGNTEPGDGRRYKGRGPIQLTGRANYRAAGEALGIDLEGNPERAADPDVAFRIATWYWQTRGLNTLADQGNFREITRRINGGYNGMDDRERYYQRAREVF

>WP_302052631.1 glycoside hydrolase family 19 protein, partial [Corallococcus exiguus]

GGTPGTSSGKGGVTVAQLRRIMPNLSQAKAEQYLPHLNKAMAEANINTPRRKEMFLAQLAHESGELRYMEEIASGAAYEGRKDLGNTQPGDGKRYKGRGPIQLTGRANYRAAGKALGIDLEGHPERAKDPDVAFRIAGWYWQSRNLNSYADAGNFREVTRRINGGYNGLASREMYYRRAQDVLG

>OJT24651.1 hypothetical protein BO221_14135 [Archangium sp. Cb G35]

MQQAGPVSIEQLKAIMPRAPAAKASEYLGHLNAAMREREITSPKRAAAFLAQLAHESGELRYMEEIASGAAYEGRVDLGNTQTGDGKRYKGRGPIQLTGRANYRAAGKALGVALEDNPTRAAEPDVAFRVAAWYWDSRGLNSLADTGNFREITRLINGGYNGMADREAFWAKAKATLGAA

>WP_279635579.1 MULTISPECIES: glycoside hydrolase family 19 protein, partial [Corallococcus]

GGVTVAQLRRIMPNLSQAKAEQYLPHLNKAMAEANINTPRRKEMFLAQLAHESGELRYMEEIASGAAYEGRKDLGNTQPGDGKRYKGRGPIQLTGRANYRAAGKALGIDLEGHPERAKDPDVAFRIAGWYWQSRNLNSYADAGNFREVTRRINGGYNGLASREMYYRRAQDVLG

>WP_206794809.1 MULTISPECIES: glycoside hydrolase family 19 protein [unclassified Corallococcus]

MILESLVLAAVAAAGAPKVVKKMKPTLDLSGLTLAQLQAIMPNLSTALAQEYAPLLALAMNEAAINTPIRRAAFLAQLAHESGEFRWMEEIASGAAYEGRKDLGNTQPGDGIRYKGRGPIQVTGRANYRDAGQALGIDLENNPKRAAEPAIGFRTAAWYWTSRNINARADAGDLVGVTKLINGGTNGLEDRRKFYDRAVKVLGVTA

>OJT27696.1 hypothetical protein BO221_04870 [Archangium sp. Cb G35]

MVAAVVLAVGVAVASGGGLLTLEQLRAIMPRLAQARAEELLSHLVAAMAEAGITTPRRKAAFLAQLAHESAEFRYFEELASGDAYEGRRDLGNTQPGDGRRYKGRGPIQLTGRNNYRDAGRALGVDLEGKPELAATPAVGFRTAAWFWRTRELNELADAGNFDAITKRINGGYNGKADRDAYHVRAQAALGAA

>WP_143195445.1 glycoside hydrolase family 19 protein [Archangium sp. Cb G35]

MPRNVGFILLAAAVVAVGAYVLMQQAGPVSIEQLKAIMPRAPAAKASEYLGHLNAAMREREITSPKRAAAFLAQLAHESGELRYMEEIASGAAYEGRVDLGNTQTGDGKRYKGRGPIQLTGRANYRAAGKALGVALEDNPTRAAEPDVAFRVAAWYWDSRGLNSLADTGNFREITRLINGGYNGMADREAFWAKAKATLGAA

>WP_301340410.1 glycoside hydrolase family 19 protein, partial [Corallococcus exercitus]

KGGVTAAQLRRIMPNLSQAKAEQYLPHLNKAMAEANINTPRRKEMFLAQLAHESGELRYMEEIASGAAYEGRKDLGNTQPGDGKRYKGRGPIQLTGRANYRAAGKALGIDLEGHPERAKDPDVAFRIAGWYWQSRNLNSYADAGNFREVTRRINGGYNGLASREMYYRRAQDVLG

>WP_208647366.1 glycoside hydrolase family 19 protein, partial [Corallococcus interemptor]

GGVTAAQLRRIMPNLSQAKAEQYLPHLNKAMAEANINTPRRKEMFLAQLAHESGELRYMEEIASGAAYEGRKDLGNTQPGDGKRYKGRGPIQLTGRANYRAAGKALGIDLEGHPERAKDPDVAFRIAGWYWQSRNLNSYADAGNFREVTRRINGGYNGLASREMYYRRAQDVLG

>WP_233165669.1 glycoside hydrolase family 19 protein [Archangium sp. Cb G35]

MSRGAWLALVAAVVLAVGVAVASGGGLLTLEQLRAIMPRLAQARAEELLSHLVAAMAEAGITTPRRKAAFLAQLAHESAEFRYFEELASGDAYEGRRDLGNTQPGDGRRYKGRGPIQLTGRNNYRDAGRALGVDLEGKPELAATPAVGFRTAAWFWRTRELNELADAGNFDAITKRINGGYNGKADRDAYHVRAQAALGAA

>WP_283247258.1 glycoside hydrolase family 19 protein, partial [Corallococcus exiguus]

GTPGTSGGKAGVTVAQLRRIMPNLSQAKAEQYLPHLNKAMAEANINTPRRKEMFLAQLAHESGELRYMEEIASGAAYEGRKDLGNTQPGDGKRYKGRGPIQLTGRANYRAAGKALGIDLEGHPERAKDPDVAFRIAGWYWGSRNLNSYADAGNFREVTRRINGGYNGLASREMYYRRAQDVLG

>WP_304997775.1 glycoside hydrolase family 19 protein, partial [Corallococcus sp. AB032C]

GKGGVTVAQLRRIMPNLSQAKAEQYLPHLNKAMAEANINTPRRKEMFLAQLAHESGELRYMEEIASGAAYEGRKDLGNTQPGDGKRYKGRGPIQLTGRANYRAAGKALGIDLEGHPERAKDPDVAFRIAGWYWGSRNLNSYADAGNFREVTRRINGGYNGLASREMYYRRAQDVLG

>WP_301540719.1 glycoside hydrolase family 19 protein, partial [Corallococcus exiguus]

KGGVTVAQLRRIMPNLSQAKAEQYLPHLNKAMAEANINTPRRKEMFLAQLAHESGELRYMEEIASGAAYEGRKDLGNTQPGDGKRYKGRGPIQLTGRANYRAAGKALGIDLEGHPERAKDPDVAFRIAGWYWGSRNLNSYADAGNFREVTRRINGGYNGLASREMYYRRAQDVLG

>WP_301540901.1 glycoside hydrolase family 19 protein, partial [Corallococcus exiguus]

DRGGVTVAQLRRIMPNLSQAKAEQYLPHLNKAMAEANINTPRRKEMFLAQLAHESGELRYMEEIASGAAYEGRKDLGNTQPGDGKRYKGRGPIQLTGRANYRAAGKALGIDLEGHPERAKDPDVAFRIAGWYWGSRNLNSYADAGNFREVTRRINGGYNGLASREMYYRRAQDVLG

>WP_199729149.1 glycoside hydrolase family 19 protein, partial [Corallococcus sp. CA053C]

KGGVSVAQLRKIMPNLSQAKAEQYLPHLNKAMAEAKINTPKRQEMFLAQLAHESGELRYMEEIASGAAYEGRKDLGNTQPGDGKRYKGRGPIQLTGRANYRAAGKALGIDLEGHPERAKDPDVAFRIAGWYWGSRNLNSYADAGNFREVTRRINGGYNGMASREMYYRRAQGVLG

>NOK38358.1 LysM peptidoglycan-binding domain-containing protein [Corallococcus exercitus]

MTTYSVRSGDTLSGLAQRFNTSVGSLQKTNHIANANLIRVGQRLTVPDGFQAAPSKAGSYTVRSGDTLSGIAGRHGTTTAALAKANHIANPNKIYVGQKLTIPGAGGGSAPVTSKPPSSGGASYTVRSGDTLSGIAGRYGTTVGALQQANHISNPNKIFVGQKLTIPGRTGGTGGTSKPPPSTGGVNGTPGTSGGKGGVTVAQLRRIMPNLSQAKAEQYLPHLNKAMAEANINTPRRKEMFLAQLAHESGELRYMEELASGAAYEGRKDLGNTQPGDGKRYKGRGPIQLTGRANYRAAGKALGIDLEGHPERAKDPDVAFRIAGWYWQSRNLNSYADAGNFREVTRRINGGYNGLASREMYYRRAQDVLG

>MDP9151424.1 hypothetical protein [Myxococcota bacterium]

MKRRRDLSPGESFQESVCSAAIGVRDALAHSERVASEIYSDMRGLSAARVPRLDERELEAVIRAFSRGDYVIFAANPPSRQHCEGGVSSEQLHAIMPSLSHDLTVAYLPLLNAAMLEAKITTPLRQAAFLAQLAHESGELRAFHEFHTGDQYEGRKDIGNTQPGDGRRFKGRGPIQVTGRATYRAAGSALGLPLEDQPELVATPEVGFRVAGWFWTAKHLDHLADARDFDGITHRVNGGFRGKASRDHYYGVAKRVLGVPEP

>WP_140858041.1 LysM peptidoglycan-binding domain-containing protein [Myxococcus xanthus]

MTTYSVRRGDTLSALAQRFNTSVSSLAKSNGISNPNLIYAGQQLRIPDGFDAPRASGGGRAASSYTVKSGDTLSGIAGRYGTSVGALATANNISNPNLIYAGQRLTIPGGGGASPTRPTPNQPPVGGVGGPKPPTGGSAGVTVQQLRAVMPNLSQATAEQYLPHLNRAMAEANITTPMRKAAFLAQLAHESGQLRYMEEIASGAAYEGRRDLGNTQPGDGVRYKGRGPIQLTGRANYRAAGQALGIDLEGNPQRAKDPDVAFRIAGWYWSSRNLNTYADAGNFREVTRRINGGYNGMADREMYYRRAQNVF

>WP_284663406.1 LysM peptidoglycan-binding domain-containing protein [Myxococcus sp. SDU36]

MTTYSVRRGDTLSALAQRFNTSVSSLAKSNGISNPNLIYAGQQLRIPDGFDAPRASGGGRAASSYTVKSGDTLSGIAGRHGTSVSALAKANNISNPDRIYAGQKLTIPGTGGAAPSSPAPSSGGGSYTVKSGDTLSGIAGRHGTSVSALAKANNISNPNLIYVGQRLTIPGGGGASPTRPTPNPPPVGGVGGPKPPTGGSAGVTVQQLRAVMPNLSQAKAEQYLPHLNRAMAEANITTPIRKAAFLAQLAHESGQLRYMEEIASGAAYEGRRDLGNTQPGDGVRYKGRGPIQLTGRANYRAAGQALGIDLEGNPQRAKDPDVAFRIAGWYWSSRNLNTYADAGNFREVTRRINGGYNGLADREMYYRRAQNVF

>WP_279637941.1 glycoside hydrolase family 19 protein, partial [Corallococcus aberystwythensis]

GKGGVTAAQLRRIMPNLSQAKAEQYLPHLNKAMAEAKINTPRRQEMFLAQLAHESGELRYMEEIASGAAYEGRKDLGNTQPGDGKRYKGRGPIQLTGRANYRAAGKALGIDLEGHPERAKDPDVAFRIAGWYWGSRNLNSYADAGNFREVTRRINGGYNGLASREMYYRRAQDVLG

>WP_120529290.1 LysM peptidoglycan-binding domain-containing protein [Corallococcus exercitus]

MTTYSVRSGDTLSGLAQRFNTSVGSLQKTNHIANANLIRVGQRLTVPDGFQAAPSKAGSYTVRSGDTLSGIAGRHGTTTAALAKANHIANPNKIYVGQKLTIPGAGGGSAPVTSKPPSSGGASYTVRSGDTLSGIAGRYGTTVGALQQANHISNPNKIFVGQKLTIPGRTGGTGGTSKPPPSTGGVNGTPGTSGGKGGVTVAQLRRIMPNLSQAKAEQYLPHLNKAMAEANINTPRRKEMFLAQLAHESGELRYMEEIASGAAYEGRKDLGNTQPGDGKRYKGRGPIQLTGRANYRAAGKALGIDLEGHPERAKDPDVAFRIAGWYWQSRNLNSYADAGNFREVTRRINGGYNGLASREMYYRRAQDVLG

>NBC44946.1 LysM peptidoglycan-binding domain-containing protein [Corallococcus exiguus]

MTTYSVRSGDTLSGLAQRFSTSVSSLQKTNHIANANLIRVGQRLTVPDGFQAAPSKAGSYTVRSGDTLSGIAGRHGTTVGALAKANHIANPNKIYVGQRLTIPGAGGGAAPVTSKPPSSGGASYTVRSGDTLSGIAGRYGTTVGALQQANHIADPNKIQVGQKLTIPGRTGGTGGTSKPPPSTGGVGGTPGTSSGKGGVTVAQLRRIMPNLSQAKAEQYLPHLNKAMAEANINTPRRKEMFLAQLAHESGELRYMEEIASGAAYEGRKDLGNTQPGDGKRYKGRGPIQLTGRANYRAAGKALGIDLEGHPERAKDPDVAFRIAGWYWQSRNLNSYADAGNFREVTRRINGGYNGLASREMYYRRAQDVLG

>WP_121754207.1 LysM peptidoglycan-binding domain-containing protein [Corallococcus sp. AB030]

MTTYSVRSGDTLSGLAQRFSTSVSSLQKTNHIANANLIRVGQRLTVPDGFQAAPSKAGSYTVRSGDTLSGIAGRHGTTVGALAKANHIANPNKIYVGQRLTIPGAGGGAAPVTSKPPPSSGGASYTVRSGDTLSGIAGRYGTTVGALQQANHIADPNKIQVGQKLTIPGRTGGSGGTSKPPPSTGGVGGTPGTSSGKGGVTVAQLRRIMPNLSQAKAEQYLPHLNKAMAEANINTPRRKEMFLAQLAHESGELRYMEEIASGAAYEGRKDLGNTQPGDGKRYKGRGPIQLTGRANYRAAGKALGIDLEGHPERAKDPDVAFRIAGWYWQSRNLNSYADAGNFREVTRRINGGYNGLASREMYYRRAQDVLG

>WP_120564554.1 LysM peptidoglycan-binding domain-containing protein [Corallococcus sp. AB011P]

MTTYSVRSGDTLSGLAQRYNTSVASLQKTNHIANANLIRVGQRLTVPDGFQAAPSKAGSYTVRSGDTLSGIAGRHGTTVGALAKANHIANPNKIYVGQRLTIPGAGGGAAPVTSKPPSSGGASYTVRSGDTLSGIAGRYGTTVGALQQANHIADPNKIYVGQKLTIPGRTGGTGGTSKPPPSTGGVGGTPGTSGGKGGVTVAQLRRIMPNLSQAKAEQYLPHLNKAMAEANINTPRRKEMFLAQLAHESGELRYMEEIASGAAYEGRKDLGNTQPGDGKRYKGRGPIQLTGRANYRAAGKALGIDLEGNPERAKDPDVAFRIAGWYWQSRNLNSYADAGNFREVTRRINGGYNGLASREMYYRRAQDVLG

>WP_167548591.1 LysM peptidoglycan-binding domain-containing protein [Corallococcus exiguus]

MIPSFPGVPFVTTYSVRSGDTLSGLAQRFSTSVSSLQKTNHIANANLIRVGQRLTVPDGFQAAPSKAGSYTVRSGDTLSGIAGRHGTTVGALAKANHIANPNKIYVGQRLTIPGAGGGAAPVTSKPPSSGGASYTVRSGDTLSGIAGRYGTTVGALQQANHIADPNKIQVGQKLTIPGRTGGTGGTSKPPPSTGGVGGTPGTSSGKGGVTVAQLRRIMPNLSQAKAEQYLPHLNKAMAEANINTPRRKEMFLAQLAHESGELRYMEEIASGAAYEGRKDLGNTQPGDGKRYKGRGPIQLTGRANYRAAGKALGIDLEGHPERAKDPDVAFRIAGWYWQSRNLNSYADAGNFREVTRRINGGYNGLASREMYYRRAQDVLG

>WP_014400746.1 LysM peptidoglycan-binding domain-containing protein [Corallococcus coralloides]

MTTYSVRSGDTLSGLAQRFNTSVASLQKTNHIANANLIRVGQRLTVPDGFQAAPSKAGSYTVRSGDTLSGIAGRHGTTVGALAKANHIANPNKIYVGQRLTIPGAGGGAAPVTSKPPPSGGASYTVRSGDTLSGIAGRYGTTVGALQQANHIADPNKIYVGQKLTIPGRTGGTGGTSKPPPSTGGVGGTPGTSGGKGGVTVAQLRRIMPNLSQAKAEQYLPHLNKAMAEANINTPRRKEMFLAQLAHESGELRYMEEIASGAAYEGRKDLGNTQPGDGKRYKGRGPIQLTGRANYRAAGKALGIDLEGHPERAKDPDVAFRIAGWYWQSRNLNSYADAGNFREVTRRINGGYNGLASREMYYRRAQDVLG

>WP_232537448.1 glycoside hydrolase family 19 protein [Cystobacter fuscus]

MSRGAWLALVAAAVLGVGVLMWKGKDVVTLAQLRAVMPRLTEARAAELLPWLLAAMREAGVSTPARAAAFLAQLAHESGELRYFEELASGAAYEGRADLGNTQPGDGVRYKGRGPIQLTGRANYRAAGTALGLDLEGSPARVATPAVGFRTAAWYWRSRGLNELADAGRFDDITRRVNGGLNGKAERDAYHAKARAVLGVTA

>WP_120577718.1 LysM peptidoglycan-binding domain-containing protein [Corallococcus sp. CA041A]

MTTYSVRSGDTLSGLAQRFSTSVSSLQKTNHIANANLIRVGQRLTVPDGFQAAPSKAGSYTVRSGDTLSGIAGRHGTTVGALAKANHIANPNKIYVGQRLTIPGAGGGAAPVTSKPPPSSGGASYTVRSGDTLSGIAGRYGTTVGALQQANHIADPNKIQVGQKLTIPGRTGGSGGTSKPPPSTGGVGGTPGTSSGKGGVTVAQLRRIMPNLSQAKAEQYLPHLNKAMAEANINTPRRKEMFLAQLAHESGELRYMEEIASGAAYEGRKDLGNTQPGDGKRYKGRGPIQLTGRANYRAAGRALGIDLEGHPERAKDPDVAFRIAGWYWQSRNLNSYADAGNFREVTRRINGGYNGLASREMYYRRAQDVLG

>WP_141256531.1 MULTISPECIES: LysM peptidoglycan-binding domain-containing protein [unclassified Myxococcus]

MTTYSVRRGDTLSALAQRFNTSVSSLAKSNGISNPNLIYAGQQLRIPDGFDAPRASGGGRAASSYTVKSGDTLSGIAGRYGTSVGALAKANNISNPDRIYAGQTLTIPGAGGAAPSSPAPSSGGGSYTVKSGDTLSGIAGRHGTTVSALANANNISNPNLIYAGQRLTIPGGGGASPTRPTPNQPPVGGVGGPKPPTGGSAGVTVQQLRAVMPNLSQATAEQYLPHLNRAMAEANITTPMRKAAFLAQLAHESGQLRYMEEIASGAAYEGRRDLGNTQPGDGVRYKGRGPIQLTGRANYRAAGQALGIDLEGNPQRAKDPDVAFRIAGWYWSSRNLNTYADAGNFREVTRRINGGYNGMADREMYYRRAQNVF

>WP_163999114.1 LysM peptidoglycan-binding domain-containing protein [Pyxidicoccus caerfyrddinensis]

MTTYSVRSGDTLGALARRFNTSTDKLAKANGISNPNKIFVGQKLVVDGFDAPRATSKGSGGSSYTVKSGDTLSGIAGRHGTTVGALQKANNISNPNLIRVGQKLTIPGSGGAAAPSKPSSGGGGSYTVKSGDTLSGIAGRYGTSVSALQKANNISNPNLIRVGQKLTIPGGGAAPGKPSNPPPVGGVNGPKPAPGGSAGVTVAQLRGVMPNLSEAKAKQYLPYLNQAMAEANITTPQRKEMFLAQLAHESGELRYMEEIASGAAYEGRKDLGNTQPGDGKRYKGRGPIQLTGRANYRAAGKALGIDLEGHPERAKDPDVAFRIAGWYWQSRNLNSYADQGNFREVTRRINGGYNGMASREAYYHRAQNVF

>WP_140878089.1 MULTISPECIES: LysM peptidoglycan-binding domain-containing protein [Myxococcus]

MTTYSVRRGDTLSALAQRFNTSVSSLAKSNGISNPNLIYAGQQLRIPDGFDAPRASGGGRAASSYTVKSGDTLSGIAGRYGTSVGALAKANNISNPDRIYAGQKLTIPGTGGAAPSSPAPSSGGGSYTVKSGDTLSGIAGRYGTSVGALATANNISNPNLIYAGQRLTIPGGGGASPTRPTPNQPPVGGVGGPKPPTGGSAGVTVQQLRAVMPNLSQATAEQYLPHLNRAMAEANITTPMRKAAFLAQLAHESGQLRYMEEIASGAAYEGRRDLGNTQPGDGVRYKGRGPIQLTGRANYRAAGQALGIDLEGNPQRAKDPDVAFRIAGWYWSSRNLNTYADAGNFREVTRRINGGYNGMADREMYYRRAQNVF

>WP_140800514.1 LysM peptidoglycan-binding domain-containing protein [Myxococcus xanthus]

MTTYSVRRGDTLSALAQRFNTSVSSLAKSNGISNPNLIYAGQQLRIPDGFDAPRASGGGRAASSYTVKSGDTLSGIAGRYGTSVGALATANNISNPDRIYAGQKLTIPGTGGAAPSSPAPSSGGGSYTVKSGDTLSGIAGRYGTSVGALAKANNISNPNLIYAGQRLTIPGGGGASPTRPTPNQPPVGGVGGPKPPTGGSAGVTVQQLRAVMPNLSQATAEQYLPHLNRAMAEANITTPMRKAAFLAQLAHESGQLRYMEEIASGAAYEGRRDLGNTQPGDGVRYKGRGPIQLTGRANYRAAGQALGIDLEGNPQRAKDPDVAFRIAGWYWSSRNLNTYADAGNFREVTRRINGGYNGMADREMYYRRAQNVF

>WP_140796183.1 LysM peptidoglycan-binding domain-containing protein [Myxococcus xanthus]

MTTYSVRRGDTLSALAQRFNTSVSSLAKSNGISNPNLIYAGQQLRIPDGFDAPRASGGGRAASSYTVKSGDTLSGIAGRYGTSVGALAKANNISNPDRIYAGQKLTIPGTGGAAPSSPAPSSGGGSYTVKSGDTLSGIAGRYGTSVGALANANNISNPNLIYAGQRLTIPGGGGASPTRPTPNQPPVGGVGGPKPPTGGSAGVTVQQLRAVMPNLSQATAEQYLPHLNRAMAEANITTPMRKAAFLAQLAHESGQLRYMEEIASGAAYEGRRDLGNTQPGDGVRYKGRGPIQLTGRANYRAAGQALGIDLEGNPQRAKDPDVAFRIAGWYWSSRNLNTYADAGNFREVTRRINGGYNGMADREMYYRRAQNVF

>WP_163779871.1 LysM peptidoglycan-binding domain-containing protein [Myxococcus vastator]

MTTYSVRRGDTLSALAQRFNTSVSSLAKSNGISNPNLIYAGQQLRIPDGFDAPRASGGGRAASSYTVKSGDTLSGIAGRHGTSVSALAKANNISNPDRIYAGQKLTIPGTGGAAPSSPAPSSGGGSYTVKSGDTLSGIAGRHGTSVSALAKANNISNPNLIYVGQRLTIPGGGGASPTRPTPNQPPVGGVGGPKPPTGGSAGVTVQQLRAVMPNLSQAKAEQYLPHLNRAMAEANITTPMRKAAFLAQLAHESGQLRYMEEIASGAAYEGRRDLGNTQPGDGVRYKGRGPIQLTGRANYRAAGQALGIDLEGNPQRAKDPDVAFRIAGWYWSSRNLNTYADAGNFREVTRRINGGYNGLADREMYYRRAQNVF

>NVJ00283.1 LysM peptidoglycan-binding domain-containing protein [Myxococcus sp. AM009]

MTTYSVRRGDTLSALAQRFKTSVSSLAKSNGISNPNLIYAGQQLRIPDGFDAPRASGGGRAASSYTVKSGDTLSGIAGRHGTSVSALAKANNISNPDRIYAGQRLTIPGTGGAAPSSPAPSSGGGSYTVKSGDTLSGIAGRHGTSVSALAKANNISNPNLIYVGQRLTIPGGGGASPTRPTPNQPPVGGVGGPKPPTGGSAGVTVQQLRAVMPNLSQAKAEQYLPHLNRAMAEANITTPMRKAAFLAQLAHESGQLRYMEEIASGAAYEGRRDLGNTQPGDGVRYKGRGPIQLTGRANYRAAGQALGIDLEGNPQRAKDPDVAFRIAGWYWSSRNLNTYADAGNFREVTRRINGGYNGLADREMYYRRAQNVF

>WP_279636142.1 glycoside hydrolase family 19 protein, partial [Corallococcus terminator]

TPGTSGGKGGVSVAQLRKIMPNLSQAKAEQYLPHLNKAMAEAKINTPKRQEMFLAQLAHESGELRYMEEIASGAAYEGRKDLGNTQPGDGKRYKGRGPIQLTGRANYRAAGKALGIDLEGNPARAKDPDVAFRIAGWYWGSRNLNSYADAGNFREVTRRINGGYNGMASREMYYRRAQDVLG

>NVJ08093.1 LysM peptidoglycan-binding domain-containing protein [Myxococcus sp. AM001]

MTTYSVRRGDTLSALAQRFKTSVSSLAKSNGISNPNLIYAGQQLRIPDGFDAPRASGGGRAASSYTVKSGDTLSGIAGRHGTSVSALAKANNISNPDRIYAGQKLTIPGTGGAAPSSPAPSSGGGSYTVKSGDTLSGIAGRHGTSVSALAKANNISNPNLIYVGQRLTIPGGGGASPTRPTPNQPPVGGVGGPKPPTGGSAGVTVQQLRAVMPNLSQAKAEQYLPHLNRAMAEANITTPMRKAAFLAQLAHESGQLRYMEEIASGAAYEGRRDLGNTQPGDGVRYKGRGPIQLTGRANYRAAGRALGIDLEGNPQRAKDPDVAFRIAGWYWSSRNLNTYADAGNFREVTRRINGGYNGLADREMYYRRAQNVF

>WP_128800147.1 LysM peptidoglycan-binding domain-containing protein [Corallococcus coralloides]

MTTYSVRSGDTLSGLAQRFNTSVSSLQKTNHIANANLIRVGQRLTVPDGFQAAPSKAGSYTVRSGDTLSGIAGRHGTTVGALAKANGIANPNKIYVGQRLTIPGAGGGAAPVTSKPPSGGASYTVRSGDTLSGIAGRYGTTVGALQQANRIADPNKIQVGQKLTIPGRTGGTGGTSKPPPSTGGVGGTPGTSGGKGGVSVAQLRRIMPNLSQAKAEQYLPHLNKAMAEANINTPRRKEMFLAQLAHESGELRYMEEIASGAAYEGRKDLGNTQPGDGKRYKGRGPIQLTGRANYRAAGKALGIDLEGHPERAKDPDVAFRIAGWYWQSRNLNSYADAGNFREVTRRINGGYNGLASREMYYRRAQDVLG

>WP_201423155.1 LysM peptidoglycan-binding domain-containing protein [Myxococcus xanthus]

MTIYSVRRGDTLSALAQRFNTSVSSLAKSNGISNPDLIYAGQQLRIPDGFDAPRASGGGSYTVKSGDTLSGIAGRHGTSVGALAKANNISNPNLIYAGQRLTIPGGGGAAPSSGGGSYTVKSGDTLSGIAGRYGTSVGALASANNISNPNLIYAGQRLTIPGGGGASPTRPAPNQPPVGGVGGPKPPTGGSAGVTVQQLRAVMPNLSQAKAEQYLPHLNRAMAEANITTPMRKAAFLAQLAHESGQLRYMEEIASGAAYEGRRDLGNTQPGDGVRYKGRGPIQLTGRANYRAAGQALGIDLEGNPQRAKDPDVAFRIAGWYWSSRNLNTYADAGNFREVTRRINGGYNGMADREMYYRRAQNVF

>WP_140870090.1 LysM peptidoglycan-binding domain-containing protein [Myxococcus xanthus]

MTTYSVRRGDTLSALAQRFNTSVSSLAKSNGISNPNLIYAGQQLRIPDGFDAPRASGGGRAASSYTVKSGDTLSGIAGRYGTSVGSLAKANNISNPDRIYAGQKLTIPGTGGAAPSSPAPSSGGGSYTVKSGDTLSGIAGRHGTTVSALARANNISNPNLIYAGQRLTIPGGGGASPTRPTPNQPPVGGVGGPKPPTGGSAGVTVQQLRAVMPNLSQAKAEQYLPHLNRAMAEANITTPMRKAAFLAQLAHESGQLRYMEEIASGAAYEGRRDLGNTQPGDGVRYKGRGPIQLTGRANYRAAGQALGIDLEGNPQRAKDPDVAFRIAGWYWSSRNLNTYADAGNFREVTRRINGGYNGMADREMYYRRAQNVF

>WP_217917550.1 MULTISPECIES: LysM peptidoglycan-binding domain-containing protein [unclassified Myxococcus]

MFSGVCPPVTTYSVRRGDTLSALAQRFKTSVSSLAKSNGISNPNLIYAGQQLRIPDGFDAPRASGGGRAASSYTVKSGDTLSGIAGRHGTSVSALAKANNISNPDRIYAGQRLTIPGTGGAAPSSPAPSSGGGSYTVKSGDTLSGIAGRHGTSVSALAKANNISNPNLIYVGQRLTIPGGGGASPTRPTPNQPPVGGVGGPKPPTGGSAGVTVQQLRAVMPNLSQAKAEQYLPHLNRAMAEANITTPMRKAAFLAQLAHESGQLRYMEEIASGAAYEGRRDLGNTQPGDGVRYKGRGPIQLTGRANYRAAGQALGIDLEGNPQRAKDPDVAFRIAGWYWSSRNLNTYADAGNFREVTRRINGGYNGLADREMYYRRAQNVF

>WP_301541145.1 glycoside hydrolase family 19 protein, partial [Corallococcus exiguus]

AQLRRIMPNLSQAKAEQYLPHLNKAMAEANINTPRRKEMFLAQLAHESGELRYMEEIASGAAYEGRKDLGNTQPGDGKRYKGRGPIQLTGRANYRAAGKALGIDLEGHPERAKDPDVAFRIAGWYWGSRNLNSYADAGNFREVTRRINGGYNGLASREMYYRRAQDVLG

>WP_090491231.1 LysM peptidoglycan-binding domain-containing protein [Myxococcus virescens]

MTIYSVRRGDSLSALAQRFNTSVSSLAKSNGISNPDLIYAGQKLRIPDGFDAPRASGGGSYTVKSGDTLSGIAGRHGTSVGALAKANNISNPDLIYAGQRLTIPGGGGASPSSGGGSYTVKSGDTLSGIAGRYGTSVGALARANNISNPNLIYAGQRLTIPGGGGASPTRPAPNQPPVGGVGGPKPPTGGSAGVTVQQLRAVMPNLSQAKAEQYLPHLNRAMAEANITTPMRKAAFLAQLAHESGQLRYMEEIASGAAYEGRRDLGNTQPGDGVRYKGRGPIQLTGRANYRAAGQALGIDLEGNPQRAKDPDVAFRIAGWYWSSRNLNTYADAGNFREVTRRINGGYNGMADREMYYRRAQNVF

>NOJ80135.1 LysM peptidoglycan-binding domain-containing protein [Myxococcus xanthus]

MTTYSVRRGDTLSALAQRFNTSVSSLAKSNGISNPNLIYAGQQLRIPDGFDAPRASGGGRAASSYTVKSGDTLSGIAGRYGTSVGALAKANNISNPDRIYAGQRLTIPGTGGAAPSSPAPSSGGGSYTVKSGDTLSGIAGRHGTTVSALARANNISNPNLIYAGQRLTIPGGGGASPTRPTPNQPPVGGVGGPKPPTGGSAGVTVQQLRAVMPNLSQAKAEQYLPHLNRAMAEANITTPMRKAAFLAQLAHESGQLRYMEEIASGAAYEGRRDLGNTQPGDGVRYKGRGPIQLTGRANYRAAGQALGIDLEGNPQRAKDPDVAFRIAGWYWSSRNLNTYADAGNFREVTRRINGGYNGMADREMYYRRAQNVF

>WP_141588517.1 LysM peptidoglycan-binding domain-containing protein [Myxococcus sp. AB056]

MTIYSVRRGDSLSALAQRFNTSVSSLAKSNGISNPDLIYAGQKLRIPDGFDAPRASGGGSYTVKSGDTLSGIAGRHGTSVGALAKANNISNPNLIYAGQRLTIPGGGGASPSSGGGSYTVKSGDTLSGIAGRYGTSVGALAKANNISNPNLIYAGQRLTIPGGGGASPTRPAPNKPPVGGVGGPKPPTGGSAGVTVQQLRAVMPNLSQAKAEQYLPHLNRAMAEANITTPMRKAAFLAQLAHESGQLRYMEEIASGAAYEGRRDLGNTQPGDGVRYKGRGPIQLTGRANYRAAGRALGIDLEGNPQRAKDPDVAFRIAGWYWSSRNLNTYADAGNFREVTRRINGGYNGMADREMYYRRAQNVF

>MBN8230280.1 LysM peptidoglycan-binding domain-containing protein [Corallococcus macrosporus]

MTTYSVRSGDTLSGLAQRFNTSVGSLQKTNHIANANLIRVGQRLTVPDGFQAAPSKAGSYTVRSGDTLSGIAGRHGTTVGALAKANHITNPNKIYVGQRLTIPGAGGGASPVTSKPPSSGGASYTVRSGDTLSGIAGRYGTTVGALQQANHISNPNKIYVGQKLTIPGRTGGTGGTSKPPPSTGGVGGTPGTSGGKGGVTAAQLRRIMPNLSQAKAEQYLPHLNKAMAEANINTPRRKEMFLAQLAHESGELRYMEEIASGAAYEGRKDLGNTQPGDGKRYKGRGPIQLTGRANYRAAGKALGIDLEGHPERAKDPDVAFRIAGWYWQSRNLNSYADAGNFREVTRRINGGYNGLASREMYYRRAQDVLG

>WP_011557299.1 MULTISPECIES: LysM peptidoglycan-binding domain-containing protein [Myxococcus]

MTIYSVRRGDTLSALAQRFNTSVSSLAKSNGISNPDLIYAGQQLRIPDGFDAPRASGGGSYTVKSGDTLSGIAGRHGTSVGALAKANNISNPDRIYAGQRLTIPGAGGAAPSSGGGSYTVKPGDTLSGIAGRYGTSVGALAQANNISNPNLIYAGQRLTIPGGGGASPTRPAPNQPPVGGVGGPKPPTGGSAGVTVQQLRAVMPNLSQAKAEQYLPHLNRAMAEANITTPMRKAAFLAQLAHESGQLRYMEEIASGAAYEGRRDLGNTQPGDGVRYKGRGPIQLTGRANYRAAGQALGIDLEGNPQRAKDPDVAFRIAGWYWSSRNLNTYADAGNFREVTRRINGGYNGMADREMYYRRAQNVF

>RKH89298.1 glycoside hydrolase family 19 protein [Corallococcus sp. AB045]

MPNLSQAKAEQYLPHLNKAMAEANINTPRRKEMFLAQLAHESGELRYMEEIASGAAYEGRKDLGNTQPGDGKRYKGRGPIQLTGRANYRAAGKALGIDLEGNPERAKDPDVAFRIAGWYWQSRNLNSYADAGNFREVTRRINGGYNGLASREMYYRRAQDVLG

>WP_002638152.1 LysM peptidoglycan-binding domain-containing protein [Myxococcus hansupus]

MTTYSVRSGDTLGALAKRFNTSVSSLAKTNGISNPNLIFTGQKLRVPDGFDAPRASGGGRAASSYTVKSGDTLSGIAGRHGTTVSALAKANNISNPNRIYAGQKLTIPGSGGAAPTKPSSGGGGGGSYTVKSGDTLSGIASRHGTTVSALARANNISNPNLIHVGQRLTIPGGGGASPTRPTPSQPPVGGVGGPKPPTGGSAGVTVGQLRAVMPNLSQAKAEQYLPYLNQAMAEANITTPMRKAAFLAQLAHESGQLRYMEEIASGAAYEGRRDLGNTQPGDGVRYKGRGPIQLTGRANYRAAGRALGIDLEGNPQRAKDPDVAFRIAGWYWSSRNLNTYADAGNFREVTRRINGGYNGLADREMYYRRAQNVF

>WP_141620909.1 LysM peptidoglycan-binding domain-containing protein [Myxococcus sp. AB036A]

MTIYSVRRGDSLSALAQRFNTSVSSLAKSNGISNPDLIYAGQKLRIPDGFDAPRASGGGSYTVKSGDTLSGIAGRHGTSVGALAKANNISNPDLIYAGQRLTIPGGGGASPSSGGGSYTVKSGDTLSGIAGRYGTSVGALAKANNISNPNLIYAGQRLTIPGGGGASPTRPAPNQPPVGGVGGPKPPTGGSAGVTVQQLRAVMPNLSQAKAEQYLPHLNRAMAEANITTPMRKAAFLAQLAHESGQLRYMEEIASGAAYEGRRDLGNTQPGDGVRYKGRGPIQLTGRANYRAAGRALGIDLEGNPQRAKDPDVAFRIAGWYWSSRNLNTYADAGNFREVTRRINGGYNGMADREMYYRRAQNVF

>NNC15077.1 glycoside hydrolase family 19 protein [Corallococcus exiguus]

MPNLSQAKAEQYLPHLNKAMAEANINTPRRKEMFLAQLAHESGELRYMEEIASGAAYEGRKDLGNTQPGDGKRYKGRGPIQLTGRANYRAAGKALGIDLEGHPERAKDPDVAFRIAGWYWQSRNLNSYADAGNFREVTRRINGGYNGLASREMYYRRAQDVLG

>WP_120647374.1 LysM peptidoglycan-binding domain-containing protein [Corallococcus llansteffanensis]

MTTYSVRSGDTLSGLAQRFHTSVGTLQKTNHIANANLIRVGQKLSVPDSFQAAAPSKGGGKAGSYTVRSGDTLSGIASRHGTTVSALAKANHISNPNKIYVGQKLTIPGRSGGTGTSNPPPTTGGTGGVGGATGGTKGGVSVAQLRKIMPNLSQAKAEQYLPHLNKAMAEAKINTPKRQEMFLAQLAHESGELRYMEEIASGAAYEGRKDLGNTQPGDGKRYKGRGPIQLTGRANYRAAGKALGIDLEGHPERAKDPDVAFRIAGWYWGSRNLNSYADAGNFREVTRRINGGYNGMASREMYYRRAQGVLG

>WP_206786638.1 LysM peptidoglycan-binding domain-containing protein [Corallococcus sp. NCRR]

MTTYSVRSGDTLSGLAQRFNTSVSSLQKTNHIANANLIRVGQRLTVPDGFQAAPSKAGSYTVRSGDTLSGIAGRHGTTVGALAKANGIANPNKIYVGQRLTIPGAGGGAAPVTSKPPSSGGASYTVRSGDTLSGIAGRYGTTVGALQQANHIANPNKIYVGQKLTIPGRTGGTGGTSKPPPSTGGVGGTPGTSGGKGGVTVAQLRRIMPNLSQAKAEQYLPHLNKAMAEANINTPRRKEMFLAQLAHESGELRYMEEIASGAAYEGRKDLGNTQPGDGKRYKGRGPIQLTGRANYRAAGKALGIDLEGHPERAKDPDVAFRIAGWYWSSRNLNSYADAGNFREVTRRINGGYNGLASREMYYRRAQDVLG

>WP_120585817.1 MULTISPECIES: LysM peptidoglycan-binding domain-containing protein [Corallococcus]

MTTYSVRSGDTLSGLAQRFNTSVGSLQKTNHIANANLIRVGQKLSVPDSFQAAAPSKAGAKAGSYTVRSGDTLSGIASRHGTTTGALAKANNISNPNKIYVGQKLTIPGKGGSAPVTPKPSSGGSTYTVRSGDTLSGIAGRHGTTVNALQQANNISNPNKIYVGQKLTIPGKSGGGGTTNPPPTTGGVGGTPGTSGGKGGVSVAQLRKIMPNLSQAKAEQYLPHLNKAMAEAKINTPKRQEMFLAQLAHESGELRYMEEIASGAAYEGRKDLGNTQPGDGKRYKGRGPIQLTGRANYRAAGKALGIDLEGHPERAKDPDVAFRIAGWYWGSRNLNSYADAGNFREVTRRINGGYNGMASREMYYRRAQDVLG

>WP_120537710.1 LysM peptidoglycan-binding domain-containing protein [Corallococcus sp. CA047B]

MTTYSVRSGDTLSGLAQRFNTSVGSLQKTNHIANANLIRVGQKLTVPDSFQAAAPSKAGAKAGSYTVRSGDTLSGIASRHGTTTGALAKANNISNPNKIYVGQKLTIPGKGGSAPVTPKPSSGGSTYTVRSGDTLSGIAGRHGTTVNALQQANNISNPNKIYVGQKLTIPGKSGGGGTTNPPPTTGGVGGTPGTSGGKGGVSVAQLRKIMPNLSQAKAEQYLPHLNKAMAEAKINTPKRQEMFLAQLAHESGELRYMEEIASGAAYEGRKDLGNTQPGDGKRYKGRGPIQLTGRANYRAAGKALGIDLEGHPERAKDPDVAFRIAGWYWGSRNLNSYADAGNFREVTRRINGGYNGMASREMYYRRAQDVLG

>WP_216608962.1 LysM peptidoglycan-binding domain-containing protein [Myxococcus xanthus]

MWVRRLSVFRSLPPVTIYSVRRGDTLSALAQRFNTSVSSLAKSNGISNPDLIYAGQQLRIPDGFDAPRASGGGSYTVKSGDTLSGIAGRHGTSVGALAKANNISNPDRIYAGQRLTIPGAGGAAPSSGGGSYTVKPGDTLSGIAGRYGTSVGALAQANNISNPNLIYAGQRLTIPGGGGASPTRPAPNQPPVGGVGGPKPPTGGSAGVTVQQLRAVMPNLSQAKAEQYLPHLNRAMAEANITTPMRKAAFLAQLAHESGQLRYMEEIASGAAYEGRRDLGNTQPGDGVRYKGRGPIQLTGRANYRAAGQALGIDLEGNPQRAKDPDVAFRIAGWYWSSRNLNTYADAGNFREVTRRINGGYNGMADREMYYRRAQNVF

>WP_267551838.1 LysM peptidoglycan-binding domain-containing protein [Corallococcus sp. BB11-1]

MTTYSVRSGDTLSGLAQRFNTSVGSLQKTNGIANANLIRVGQRLTVPDSFQAAPSKGGSYTVRGGDTLSGIAGRHGTTTAALAKANNIANANKIYVGQRLTIPGKGGAAPVTQKPTSGGSTYTVRSGDTLSGIAGRHGTTVNALQQANNIANANKIYVGQKLTIPGRSGGGGTANPPPTTGGVGGTPGTSGGKGGVSVAQLRRIMPNLSQAKAEQYLPHLNKAMAEANINTPQRQEMFLAQLAHESGELRYMEEIASGAAYEGRKDLGNTQPGDGKRYKGRGPIQLTGRANYRAAGKALGIDLEGNPERAKDPDVAFRIAGWYWGSRNLNSYADAGNFREVTRRINGGYNGLASREMYYRRAQDVLG

>WP_267858054.1 LysM peptidoglycan-binding domain-containing protein [Pyxidicoccus sp. MSG2]

MTTYSVRSGDTLGALARRFNTTTDKLAKANGISNPNKIFVGQKLVVDGFDAPRATGGSSKGSSYTVKSGDTLSGIAGRHGTTVGALQKANNISNPNLIYAGQKLTIPGSGAAAPSKPASGGGGSYTVKSGDTLSGIAGRYGTSVSALQQANGISNPNKIYVGQKLTIPGGGASPGKPSNPPPVGGVNGPKPAPGGSAGVTVAQLRGVMPNLSEAKAKQYLPYLNQAMAEANITTPQRKEMFLAQLAHESGELRYMEEIASGAAYEGRKDLGNTQPGDGVRYKGRGPIQLTGRANYRAAGKALGIDLEGHPERAKDPDVAFRIAGWYWQSRNLNSYADQGNFREVTRRINGGYNGMASREAYYRRAQNVF

>NNB96740.1 LysM peptidoglycan-binding domain-containing protein [Corallococcus exiguus]

MTTYSVRSGDTLSGLAQRFSTSVSSLQKTNHIANANLIRVGQRLTVPDGFQAAPSKAGSYTVRSGDTLSGIAGRHGTTVGALAKANGIANPNKIYVGQRLTIPGAGGGAAPVTSKPPSSGGASYTVRSGDTLSGIAGRYGTTVGALQQANHIADPNKIQVGQKLTIPGRTGGSGGTSKPPPSTGGVGGTPGTSGGKAGVTVAQLRRIMPNLSQAKAEQYLPHLNKAMAEANINTPRRKEMFLAQLAHESGELRYMEEIASGAAYEGRKDLGNTQPGDGKRYKGRGPIQLTGRANYRAAGKALGIDLEGHPERAKDPDVAFRIAGWYWGSRNLNSYADAGNFREVTRRINGGYNGLASREMYYRRAQDVLG

>MBZ4334728.1 LysM peptidoglycan-binding domain-containing protein [Corallococcus sp. AS-1-12]

MTTYSVRSGDTLSGLAQRFNTSVSSLQKTNHIANANLIRVGQRLTVPDGFQAAPSKAGSYTVRSGDTLSGIAGRHGTTVGALAKANHIANPNKIYVGQRLTIPGAGGGAAPVTSKPPPSGGASYTVRSGDTLSGIAGRYGTTVGALQQANHIANPNKIYVGQKLTIPGRTGGTGGASKPPPSTGGVGGTPGTSGGKGGVTVAQLRRIMPNLSQAKAEQYLPHLNKAMAEANINTPRRKEMFLAQLAHESGELRYMEEIASGAAYEGRKDLGNTQPGDGKRYKGRGPIQLTGRANYRAAGKALGIDLEGHPERAKDPDVAFRIAGWYWGSRNLNSYADAGNFREVTRRINGGYNGLASREMYYRRAQDVLG

>WP_121762487.1 LysM peptidoglycan-binding domain-containing protein [Corallococcus sp. AB038B]

MTTYSVRSGDTLSGLAQRFSTSVSSLQKTNHIANANLIRVGQRLTVPDGFQAAPSKAGSYTVRSGDTLSGIAGRHGTTVGALAKANGIANPNKIYVGQRLTIPGAGGGAAPVTSKPPSSGGASYTVRSGDTLSGIAGRYGTTVGALQQANHIADPNKIQVGQKLTIPGRTGGSGGTSKPPPSTGGVGGTPGTSGGKGGVTVAQLRRIMPNLSQAKAEQYLPHLNKAMAEANINTPRRKEMFLAQLAHESGELRYMEEIASGAAYEGRKDLGNTQPGDGKRYKGRGPIQLTGRANYRAAGKALGIDLEGHPERAKDPDVAFRIAGWYWGSRNLNSYADAGNFREVTRRINGGYNGLASREMYYRRAQDVLG

>MBE4752116.1 LysM peptidoglycan-binding domain-containing protein [Corallococcus soli]

MTTYSVRSGDTLSGLAQRFNTSVGSLQKTNGIANANLIRVGQRLTVPDSFQAAPSKGGSYTVRGGDTLSGIAGRHGTTTAALAKANNIANPNKIYVGQRLTIPGKGGAAPVTQKPTSGGSTYTVRSGDTLSGIAGRHGTTVNALQQANDIANPNKIYVGQKLTIPGRSGGGGTANPPPTTGGVGGTPGTSGGKGGVSVAQLRRIMPNLSQAKAEQYLPHLNKAMAEANINTPQRQEMFLAQLAHESGELRYMEEIASGAAYEGRKDLGNTQPGDGKRYKGRGPIQLTGRANYRAAGKALGIDLEGNPERAKDPDVAFRIAGWYWGSRNLNSYADAGNFREVTRRINGGYNGLASREMYYRRAQDVLG

>MBN8470881.1 LysM peptidoglycan-binding domain-containing protein [Corallococcus exiguus]

MTTYSVRSGDTLSGLAQRFNTSVSSLQKTNHIANANLIRVGQRLTVPDGFQAAPSKAGSYTVRSGDTLSGIAGRHGTTVGALAKANGIANPNKIQVGQRLTIPGAGGGAAPVTSKPPSSGGASYTVRSGDTLSGIAGRYGTTVGALQQANHIADPNKIYVGQKLTIPGRTGGSGGTSKPPPSTGGVGGTPGTSGGKGGVTVAQLRRIMPNLSQAKAEQYLPHLNKAMAEANINTPRRKEMFLAQLAHESGELRYMEEIASGAAYEGRKDLGNTQPGDGKRYKGRGPIQLTGRANYRAAGKALGIDLEGHPERAKDPDVAFRIAGWYWGSRNLNSYADAGNFREVTRRINGGYNGLASREMYYRRAQDVLG

>WP_013936967.1 LysM peptidoglycan-binding domain-containing protein [Corallococcus macrosporus]

MTTYSVRRGDTLSALAQRFKTSVSSLAKTNGISNPNLIYAGQQLRIPDGFDAPRASGGGRAASSYTVKSGDTLSGIAGRHGTSVGALAKANNISNPNLIYVGQRLTIPGGGGASPTRPTPNQPPVGGVGGPKPPTGGSAGVTVQQLRAVMPNLSQAKAEQYLPHLNRAMAEANITTPKRKAAFLAQLAHESGQLRYMEEIASGAAYEGRRDLGNTQPGDGVRYKGRGPIQLTGRANYRAAGRALGIDLEGNPQRAKDPDVAFRIAGWYWSSRNLNTYADAGNFREVTRRINGGYNGMADREMYYRRALNVF

>MBL8911853.1 peptidoglycan-binding protein [Archangium sp.]

MIRPALPSPVPLPPTTPLPPTPGQIKRAENQLKKLGFSPGAVDGRATAAFTNALKEFQTAWGLPSTGTLDDATRAKLDHTIERKADSKGKLISIGEKSKRIAEFENQLARLGYSVGKRDGIYSRETAEAVKAFRADQKELKDGFGSVREGALKVLAREANALSHAPERRRVKETAASRQLDRATLDASRLGVREGDHSRVVANIQQHLRAAGFDPQRVSGTFDERTTGALKAFQRRSKLEPTGVVDSKTWRELRKARMEATSGTSPAQRLNERSGAVKASEKLLKKLGFNPGKVDGLFDKRTEKAVRAFERQQHMKVDGAIGTGQLAKMKKLSKGVTLGQLHAIMPSLPMSKARAYLPLLNRAMAEANINTRQRKAMFIAQLAHESGSLKFFEELASGAAYEGRLDLGNTQPGDGVRYKGRGPIQLTGRANYRAAGRALGLPLEAHPKMAARPSVGFRTATWFWSSRGLNALADRGDFIGVTRRINGGTNGLADRQAYYRRALKVIH

>WP_224243534.1 glycoside hydrolase family 19 protein [Cystobacter gracilis]

MSTYSVRRGDTLSSLAKRFGTTVSSLAKANNIKNVNLIRVGQKLTFKDGFDRPTTRPSTRPAGGTTQSGGAARPGGTTGTGGTQSSAPVGGPAPVGNGKGVTVEQLRKIMPSLSQAKAEQYLPHINAAMQEAGITTKKQQAAFLAQLGHESGGLRYMEEIASGAAYEGRRDLGNTQPGDGTRFKGRGPIQLTGRANYRAAGKALGLDLENNPRLAADPSVGFRTAAWFWNSRNLNSLAEAGNFREVTRRINGGYNGLADREAYYQRALNAL

>WP_120629827.1 LysM peptidoglycan-binding domain-containing protein [Corallococcus sicarius]

MKTYSVRSGDTLSGLAQRFHTSVGTLQKTNRIANANLIRVGQRLSVPDSFQAAAPSKGGSKASSYTVRSGDTLSAIAGRHGTTVGALAKANHISNPNKIYVGQKLTIPGRSGGTGTSKPPPTTGGTGGTGSVGGATGGSKGGVSVAQLRKIMPNLSQAKAEQYLPHLNKAMAEAKINTPKRQEMFIAQLAHESGELRYMEEIASGAAYEGRKDLGNTQPGDGKRYKGRGPIQLTGRANYRAAGKALGIDLEGHPERAKDPDVAFRIAGWYWGSRNLNSYADAGNFREVTRRINGGYNGMASREMYYRRAQGALG

>MCP3137254.1 LysM peptidoglycan-binding domain-containing protein [Pyxidicoccus xibeiensis]

MTTYSVRSGDTLGALARRFNTSVDALAKANGIANPNKIYAGQKLNVPDGFDAPRVSGSGGGSSYTVKSGDTLSGIAGKFGTSVSALAKANGISNPNRIFAGQRLTIPGSGGAAPSSPAPSGGSRTHTVKSGDTLSGIAGKYGTSVGALQRANNISNPNLIYVGQKLTIPGGGAAPGQPAPNPPPVGGVGGPKPVPGGSAGVTVAQLRRVMPNLSQAKAEQYLPHLNRAMAEANITTPQRKAAFLAQLAHESGELRYMEEIASGAAYEGRRDLGNTQPGDGVRYKGRGPIQLTGRANYRAAGQALGIDLEGNPARAKDPDVAFRIAGWYWSSRNLNTYADAGNFREVTRRINGGYNGMASREMYYRRAQDVF

>WP_095961344.1 LysM peptidoglycan-binding domain-containing protein [Corallococcus macrosporus]

MTTYSVRRGDTLSALAQRFKTSVSSLAKTNGISNPNLIYAGQQLRIPDGFDAPRASGGGRAASSYTVKSGDTLSGIAGRHGTSVGALAKANNISNPDRIYVGQKLTIPGSGGAAPTRPSTGGGGGGSYTVKSGDTLSGIAGRHGTTVGALAKANNISNPNLIYVGQRLTIPGGGGASPTRPTPNQPPVGGVGGPKPPTGGSAGVTVQQLRAVMPNLSQAKAEQYLPHLNRAMAEANITTPKRKAAFLAQLAHESGQLRYMEEIASGAAYEGRRDLGNTQPGDGVRYKGRGPIQLTGRANYRAAGRALGIDLEGNPQRAKDPDVAFRIAGWYWSSRNLNTYADAGNFREVTRRINGGYNGMADREMYYRRALNVF

>RKH52170.1 glycoside hydrolase family 19 protein [Corallococcus aberystwythensis]

MPNLSQAKAEQYLPHLNKAMAEAKINTPRRQEMFLAQLAHESGELRYMEEIASGAAYEGRKDLGNTQPGDGKRYKGRGPIQLTGRANYRAAGKALGIDLEGHPERAKDPDVAFRIAGWYWGSRNLNSYADAGNFREVTRRINGGYNGLASREMYYRRAQDVLG

>WP_044191749.1 glycoside hydrolase family 19 protein [Hyalangium minutum]

MIHNIRRGDTLSALAQKYGTSVSALAKANNIQNPNLIIAGKKLTIPDGFDRPGQRPATAPPGMPGSSASGRPGSTPSTTQAGPVAGPTAPAGSGVTVDQLKKIMPNLSDAKAREYLPHINNAMAEAGITTKNQKAAFLAQLAHESGQLRYMEEIASGSAYEGRRDLGNTQPGDGTRFKGRGPIQLTGRNNYRAAGQALGIDLENNPRRAADPDVGFRTAAWFWKSRGLNSLAESGNFREVTRRINGGYNGLSDRQQYYNRALSVL

>WP_143907716.1 LysM peptidoglycan-binding domain-containing protein [Corallococcus sp. Z5C101001]

MTTYSVRSGDTLSGLAQRFNTSVGSLQKTNHIANANLIRVGQRLTVPDGFQAAPSKGGSYTVRSGDTLSGIAGRHGTTVAALAKANRIADPNKIYVGQKLTIPGASGGGGAAPVPSKPTGGATYTVRSGDTLSGIAGRYGTSVGALQQANHIADPNKIYVGQKLTIPGRSGGAGTSNPPPTTGGVGGTPGTSGGKGGVSVAQLRRIMPNLSQAKAEQYLPHLNKAMAEANINTPRRKEMFLAQLAHESGELRYMEEIASGAAYEGRKDLGNTQPGDGMRYKGRGPIQLTGRANYRAAGKALGIDLEGHPERAKDPDVAFRIAGWYWNSRNLNSYADAGNFREVTRRINGGYNGLASREMYYRRAQDVLG

>NNB90683.1 glycoside hydrolase family 19 protein [Corallococcus exiguus]

MPNLSQAKAEQYLPHLNKAMAEANINTPRRKEMFLAQLAHESGELRYMEEIASGAAYEGRKDLGNTQPGDGKRYKGRGPIQLTGRANYRAAGKALGIDLEGHPERAKDPDVAFRIAGWYWGSRNLNSYADAGNFREVTRRINGGYNGLASREMYYRRAQDVLG

>RKG92766.1 glycoside hydrolase family 19 protein [Corallococcus sp. CA053C]

MPNLSQAKAEQYLPHLNKAMAEAKINTPKRQEMFLAQLAHESGELRYMEEIASGAAYEGRKDLGNTQPGDGKRYKGRGPIQLTGRANYRAAGKALGIDLEGHPERAKDPDVAFRIAGWYWGSRNLNSYADAGNFREVTRRINGGYNGMASREMYYRRAQGVLG

>NBD12723.1 LysM peptidoglycan-binding domain-containing protein [Corallococcus silvisoli]

MTTYSVRSGDTLSGLAQRFNTSVGSLQKTNHIANANLIRVGQRLTVPDGFQAAPSKGGSYTVRSGDTLSGIAGRHGTTVAALAKANHIADPNKIYVGQKLTIPGASGGGGAAPVPSKPTGGATYTVRSGDTLSGIAGRYGTSVGALQQANHIADPNKIYVGQKLTIPGRSGGAGTSNPPPATGGVGGTPGTSGGKGGVSVAQLRRIMPNLSQAKAEQYLPHLNKAMAEANINTPRRKEMFLAQLAHESGELRYMEEIASGAAYEGRKDLGNTQPGDGMRYKGRGPIQLTGRANYRAAGKALGIDLEGHPERAKDPDVAFRIAGWYWNSRNLNSYADAGNFREVTRRINGGYNGLASREMYYRRAQDVLG

>WP_206723184.1 LysM peptidoglycan-binding domain-containing protein [Pyxidicoccus parkwaysis]

MTTYSVRSGDTLGALARRFNTSVDKLAKANNISNPNLIYVGQKLVVDGFDAPRAAGGGSGGSSYTVKSGDTLSGIAGRYGTSVSALQQANGIKNPNLIYAGQKLTIPGSGGAAPTKPSGGGGGGSYTVKSGDTLSGIAGRYGTSVSALQQANGIKNPNLIYVGQKLTIPGGGSSPGKPSNPPPVGGVNGPKPAPGGSAGVTVAQLRGVMPNLSEAKAKQYLPYLNQAMAEANITTPQRKAMFLAQLAHESGELRYMEEIASGAAYEGRSDLGNTQPGDGVRYKGRGPIQLTGRANYRAAGRALGIDLEGHPERAKDPDVAFRIAGWYWSSRNLNSYADAGNFREVTRRINGGYNGMASREAYYRRAQNVF

>WP_223640269.1 LysM peptidoglycan-binding domain-containing protein [Corallococcus sp. EGB]

MTTYSVRSGDTLSGLAQRFNTSVASLQKTNHIANANLIRVGQRLTVPDGFQAAPSKAGSYTVRSGDTLSGIAGRHGTTVGALAKANHIANPNKIYVGQKLTIPGAGGGAAPVTSKPPSSGGASYTVRSGDTLSGIAGRYGTTVGALAQANHIANPNKIYVGQKLTIPGRAGGTGGTSKPPPSTGGVGGTPGTSGGKGGVSVAQLRRIMPNLSQAKAEQYLPHLNKAMAEAHINTPRRQEMFLAQLAHESGELRYMEEIASGAAYEGRADLGNTQPGDGKRYKGRGPIQLTGRANYRAAGRALGIDLEGHPERAKDPDVAFRIAGWYWSSRNLNSYADAGNFREVTRRINGGYNGLASREMYYRRAQDVLG

>WP_164019072.1 LysM peptidoglycan-binding domain-containing protein [Pyxidicoccus trucidator]

MTTYSIRSGDTLGAIARRFNTSVDKLAKANGISNPNKIYAGQKLVVDGFDAPKATGGGSGGSSYTVKSGDTLSGIAGRHGTTVAALAQANGISNPNRIFAGQRLTIPGSGGAAPSAPASGGGGGSSYTVRSGDTLSGIAGRHGTSVGALQQANGIRNPNLIYVGQKLTIPGGGAAPGRPSNPPPVGGVGGPKPAPGGSAGVTVAQLRGVMPNLSQAKAEQYLPHLNQAMAEANITTPQRKAMFLAQLAHESGQLRYMEEIASGAAYEGRRDLGNTQPGDGVRYKGRGPIQLTGRANYRAAGQALGIDLEGNPARAKDPDVAFRIAGWYWQSRNLNSYADAGNFREVTRRINGGYNGMADREAYYRRAQDVF

>MCU0696518.1 LysM peptidoglycan-binding domain-containing protein [Myxococcaceae bacterium]

MHTHRIRPGETLTMVAKANRTTVERLVAANGLRNADVVPAGTVLRLPDHFEPARTGPGAVRPSTTPTSQPSRGVTVDQLRRIMPRLPLADARRYLPFLNAAMAEVNITTPKRQAAFLAQVSHESGQLRFFEEIASGAAYEGRRDLGNVRPGDGRRFKGRGPIQLTGRANYRDAGRALGVDLENRPTRAADPDIAFRVAGWYWKTRDLNPLADRGDFVEITRRINGGVNGLADRQQAWARARAVLQA

>RKG69643.1 glycoside hydrolase family 19 protein [Corallococcus terminator]

MPNLSQAKAEQYLPHLNKAMAEAKINTPKRQEMFLAQLAHESGELRYMEEIASGAAYEGRKDLGNTQPGDGKRYKGRGPIQLTGRANYRAAGKALGIDLEGNPARAKDPDVAFRIAGWYWGSRNLNSYADAGNFREVTRRINGGYNGMASREMYYRRAQDVLG

>WP_267572988.1 LysM peptidoglycan-binding domain-containing protein [Corallococcus sp. bb12-1]

MTTYSVRSGDTLSGLAQRFNTSVGSLQKTNHIPNANLIRVGQKLTVPDSFQAAAPSGAGAKAGSYTVRSGDTLSGIAGRNGTTTVALAKANNISNPNKIYVGQKLTIPGKGGAAPVTPKPSSGGSTYTVRSGDTLSGIAGRHGTTVNALQQANNISNPNKIYVGQKLTIPGKSGGGGTTNPPPTTGGVGGVPGSSGGKGGVSVAQLRKIMPNLSQAKAEQYLPHLNKAMAEAKINTPQRQEMFLAQLAHESGELRYMEEIASGAAYEGRKDLGNTQPGDGKRYKGRGPIQLTGRANYRAAGKALGIDLEGNPARAKDPDVAFRIAGWYWGSRNLNSYADAGNFREVTRRINGGYNGMASREMYYRRAQDVLG

>WP_199738985.1 glycoside hydrolase family 19 protein, partial [Corallococcus sp. CA054B]

SYTVRSGDTLSGIAERHGTTVGAQAKAEQYLPHLNKAMAEANINTPRRKEMFLAQLAHESGELRYMEEIASGAAYEGRKDLGNTQPGDGKRYKGRGPIQLTGRANYRAAGKALGIDLEGHPERAKDPDVAFRIAGWYWQSRNLNSYADAGNFREVTRRINGGYNGLASREMYYRRAQDVLG

>WP_002616384.1 LysM peptidoglycan-binding domain-containing protein [Stigmatella aurantiaca]

MSIHPVGPGETLTGIARKYNTTVNKLAQDNGIANPDKIQVGQKLNVSGSASAARPGTDSPQNYTVRAGDTLSGIAQKFGTTTGALAKANNISNPNLIRVGQKLTIPGASASRPPSPPPSQSYTVRSGDTLSGIAQRFGTTTNALAKANNISNPNLIRVGQKLTIPGGTKPGSQDGFDPPATGGKPSTGPVTGPGNGAGTAGGVTVAQLRKVMPNLSQAKAEQYLPHLNRAMAEAKINTPKRQAAFLAQLAHESGEFRYMEEIASGAAYEGRKDLGNTQPGDGVRFKGRGPIQLTGRSNYRAAGKALGIDLENNPKRAADPDVGFRTAAWFWNSRNLNTYADAGNFREVTRRINGGYNGLASREAYYQRALNVLS

>WP_245682573.1 peptidoglycan-binding protein [Archangium gephyra]

MSLPREELRLGAQGAAVKLLQRALVELGFLPSQPGSVDGDFGPKTKAALEAFQATEKLPRDGAYTQKAREAFERRRSGGGGGLSMAQLCTIMPRLKPDKAAQYLPFLNAAMNEAEINTSLRRAAFLAQLAHESAQLRFFEELASGEAYEGRRDLGNTQKGDGVRYKGRGPIQLTGRNNYRKAGLALGLDLEGFPQRAADPDVGFRVAGWFWKSNGLNPLADAGCFDAITRRINGGYNGKADRDAHYRGALQVLGDGQPLKSV

>KFA88912.1 hypothetical protein Q664_38290 [Archangium violaceum Cb vi76]

MTVRLNEKQLREIMPRLLASRAGECLPHLDAAMVEADILTPQRQAAFLAQLAHESAELRFFEELATGEAYEGRRDLGNTRPGDGMRYKGRGPIQLTGRNNYRAAGRALGIDLEGNPQRAADVDVGFRVAAWFWTSRGLNALADVGDFREITRRINGGFNGLAQREAYYRRALEVLTH

>WP_143898266.1 MULTISPECIES: glycoside hydrolase family 19 protein [Corallococcus]

MTNTLMFRCLSLLGMVGAVGGCSGPEMGGEPPEATGRVESPAIVSTITEGDYVIRSARTNKCIDVASSSTADGATVQQWDCNGTNAQKFHISPTSGGYFKIINVNSGKGLDIKEVSTAENALVHQWSYVGGNNQQFRFVNRGGTQFSFHPRHTDMAVDLSWGSPDNGTLLVQYPYQDRDNQRWTFDLVSGGGGGGGGSGFAGILTRDTFNAMFPSRNGFYTYDALIAAAAGFPGLATTGDTDTRKREVAAFLANVSHETGGLVYIEEINKADYCDTSWGPPGCGCAPGKRYYGRGPMQLSWNGNYCAAGNALGLPLQANP

DLLAQDANAAWRTGFWFWTTQTGAGSMTAHNAIVNGAGFGETIRTINGSLECNGRNPTQVKSRVDAYKNFCALLGVSPGNNLEC

>WP_171446054.1 glycoside hydrolase family 19 protein [Corallococcus carmarthensis]

MNHKGMIRGLSLLGVVGVLGGCSGPETGGTPPEATGQVESAAVVSTITEGDYVIRSVRTNKCIDVASSSTADGAKVQQWDCNGTNAQKFHISPTSDGFFKIINVNSGKALDIAGVSTAENAVLHQWSYVGGANQQFQFVNRGGTQFSFHPRHTGMAMDLSYGSPDNGTLIVQYPFYADRDNQRWTFDLVSGGGGGGGSGFAGILTRDTFNAMFPSRNGFYTYDALIAAAATFPSVATTGDTDTRKREVAAFLANVSHETGGLVYIEEINKADYCDTSWGPPGCSCAPGKRYYGRGPMQLSWNGNYCAAGNALGLPLQANPDLLAQDANAAWRTGFWFWTTQNGAGSMTAHNAIVNGAGFGETIRTINGSLECYGRNPGQVQSRIDKYQQFCALLGVSPGNNLGC

>WP_217279631.1 chitinase, partial [Corallococcus exiguus]

TGGGGGGSGFGGILTRDMFNTMFPGRNGFYTYDALIAAANGFPGLATTGDTDTRKREVAAFLANVSHETGGLVYIEEINKGDYCDTSWGPPGCSCAPGKRYFGRGPMQLSWNGNYCAAGNALGLPLQSNPDLLAQDANAAWRTGFWFWTTQNGAGSMTAHNAIVNGAGFGETIRTINGSVECYGRNPGQVKSRVDTYKHFCSLLGVSPGNNLEC

>WP_169820487.1 glycoside hydrolase family 19 protein [Corallococcus exiguus]

MSHKGMIRGLSLLGVVGALAGCSGPEAGDTAQEAVGQVESAAVVSTITEGDYVIRSVRTNKCIDVASSSTADGAKVQQWDCNGTNAQKFHISPTSGGFWKIINVNSGKALDIVGVSTAENAVLHQWTYVDGANQQFQFINRGGVQFSFHPRHTGMAMDLSYGLPDNGTLIVQYPFYADRDNQRWTFDKVSGGGTGGGSGFGGILTRDMFNTMFPGRNGFYTYDALIAAANGFPGLATTGDTDTRKREVAAFLANVSHETGGLVYIEEINKGDYCDTSWGPPGCSCAPGKRYFGRGPMQLSWNGNYCAAGNALGLPLQANPDLLAQDANAAWRTGFWFWTTQNGAGSMTAHNAIVNGAGFGETIRTINGSLECYGRNPGQVKSRVDTYKHFCSLLGVSPGNNLEC

>WP_120600556.1 glycoside hydrolase family 19 protein [Corallococcus carmarthensis]

MNHKGMIRGLSLLGVVGVLGGCSGPETGGTPPEATGQVESAAVVSTITEGDYVIRSVRTNKCIDVASSSTADGAKVQQWDCNGTNAQKFHISPTSDGFFKIINVNSGKALDIAGVSTAENAVLHQWSYVGGANQQFQFVNRGGTQFSFHPRHTGMAMDLSYGSPDNGTLIVQYPFYADRDNQRWTFDLVSGGGGGGGSGFAGILTRDTFNAMFPSRNGFYTYDALIAAAATFPSVATTGDTDTRKREVAAFLANVSHETGGLVYIEEINKADYCDTSWGPPGCSCAPGKRYFGRGPMQLSWNGNYCAAGNALGLPLQANPDLLAQDANAAWRTGFWFWTTQNGAGSMTAHNAIVNGAGFGETIRTINGSLECYGRNPGQVQSRIDKYQQFCALLGVSPGNNLGC

>WP_121752393.1 glycoside hydrolase family 19 protein [Corallococcus sp. AB030]

MSHKGMIRGLSLLGVVGALAGCSGPEAGDTAQEAVGQVESAAVVSTITEGDYVIRSVRTNKCIDVASSSTADGAKVQQWDCNGTNAQKFHISPTSGGFWKIINVNSGKALDIVGVSTAENAVLHQWTYVDGANQQFQFINRGGVQFSFHPRHTGMAMDLSYGLPDNGTLIVQYPFYADRDNQRWTFDKVSGGGGGGGSGFGGILTRDMFNTMFPGRNGFYTYDALIAAANGFPGLATTGDTDTRKREVAAFLANVSHETGGLVYIEEINKGDYCDTSWGPPGCSCAPGKRYFGRGPMQLSWNGNYCAAGNALGLPLQANPDLLAQDANAAWRTGFWFWTTQNGAGSMTAHNAIVNGAGFGETIRTINGSVECYGRNPGQVKSRVDTYKHFCSLLGVSPGNNLEC

>WP_172813285.1 glycoside hydrolase family 19 protein [Corallococcus exiguus]

MSHKGMIRGLSLLGVVGALAGCSGPEAGDTAQEAVGQVESAAVVSTITEGDYVIRSVRTNKCIDVASSSTADGAKVQQWDCNGTNAQKFHISPTSGGFWKIINVNSGKALDIVGVSTAENAVLHQWTYVDGANQQFQFINRGGVQFSFHPRHTGMAMDLSYGLPDNGTLIVQYPFYADRDNQRWTFDKVSGGGTGGGSGFGGILTRDMFNTMFPGRNGFYTYDALIAAANGFPGLATTGDTDTRKREVAAFLANVSHETGGLVYIEEINKGDYCDTSWGPPGCSCAPGKRYFGRGPMQLSWNGNYCAAGNALGLPLQANPDLLAQDANAAWRTGFWFWTTQNGAGSMTAHNAIVNGAGFGETIRTINGSVECYGRNPGQVKSRVDTYKHFCSLLGVSPGNNLEC

>WP_120566712.1 glycoside hydrolase family 19 protein [Corallococcus sp. AB011P]

MSHKGMIRGLSLLGVVGALAGCSGPEAGDTAQEAVGQVESAAVVSTITEGDYVIRSVRTNKCIDVASSSTADGAKVQQWDCNGTNAQKFHISPTSGGFWKIINVNSGKALDIVGVSTAENAVLHQWTYVDGANQQFQFINRGGTQFSFHPRHTGMAMDLSWGSADNGTLIVQYPFYADRDNQRWTFDKVSGGGTGGGSGFGGILTRDMFNTMFPGRNGFYTYDALIAAANGFPGLATTGDTDTRKREVAAFLANVSHETGGLVYIEEINKGDYCDTSWGPPGCSCAPGKRYFGRGPMQLSWNGNYCAAGNALGLPLQSNPDLLAQDANAAWRTGFWFWTTQNGAGSMTAHNAIVNGAGFGETIRTINGSVECYGRNPGQVKSRVDTYKHFCSLLGVSPGNNLEC

>WP_139916056.1 glycoside hydrolase family 19 protein [Corallococcus exiguus]

MSHKGMIRGLSLLGVVGALAGCSGPEAGDTAQEAVGQVESAAVVSTITEGDYVIRSVRTNKCIDVASSSTADGAKVQQWDCNGTNAQKFHISPTSGGFWKIINVNSGKALDIVGVSTAENAVLHQWTYVDGANQQFQFINRGGTQFSFHPRHTGMAMDLSYGLPDNGTLIVQYPFYADRDNQRWTFDKVSGGGTGGGSGFGGILTRDMFNTMFPGRNGFYTYDALIAAANGFPGLATTGDTDTRKREVAAFLANVSHETGGLVYIEEINKGDYCDTSWGPPGCSCAPGKRYFGRGPMQLSWNGNYCAAGNALGLPLQANPDLLAQDANAAWRTGFWFWTTQNGAGSMTAHNAIVNGAGFGETIRTINGSVECYGRNPAQVKSRVDTYKHFCNLLGVSPGNNLEC

>WP_120576785.1 glycoside hydrolase family 19 protein [Corallococcus sp. CA041A]

MSHKGMIRGLSLLGVVGALAGCSGPEAGDTAQEAVGQVESAAVVSTITEGDYVIRSVRTNKCIDVASSSTADGAKVQQWDCNGTNAQKFHISPTSGGFWKIINVNSGKALDIVGVSTAENAVLHQWTYVDGANQQFQFINRGGVQFSFHPRHTGMAMDLSYGLPDNGTLIVQYPFYADRDNQRWTFDKVSGGGTGGGSGFGGILTRDMFNTMFPGRNGFYTYDALIAAANGFPGLATTGDTNTRKREVAAFLANVSHETGGLVYIEEINKGDYCDTSWGPPGCSCAPGKRYFGRGPMQLSWNGNYCAAGNALGLPLQANPDLLAQDANAAWRTGFWFWTTQNGAGSMTAHNAIVNGAGFGETIRTINGSVECYGRNPGQVKSRVDTYKHFCSLLGVSPGNNLEC

>WP_121714621.1 glycoside hydrolase family 19 protein [Corallococcus sp. AB045]

MSHKGMIRGLSLLGVVGALAGCSGPEAGDTAQEAVGQVESAAVVSTITEGDYVIRSVRTNKCIDVASSSTADGAKVQQWDCNGTNAQKFHISPTSGGFWKIINVNSGKALDIVGVSTAENAVLHQWTYVDGANQQFQFINRGGTQFSFHPRHTGMAMDLSWGSADNGTLIVQYPFYADRDNQRWTFDKVSGGGTGGGSGFGGILTRDMFNTMFPGRNGFYTYDALIAAANGVPGLATTGDTDTRKREVAAFLANVSHETGGLVYIEEINKGDYCDTSWGPPGCSCAPGKRYFGRGPMQLSWNGNYCAAGNALGLPLQSNPDLLAQDANAAWRTGFWFWTTQNGAGSMTAHNAIVNGAGFGETIRTINGSVECYGRNPGQVKSRVDTYKHFCSLLGVSPGNNLEC

>WP_120648604.1 glycoside hydrolase family 19 protein [Corallococcus llansteffanensis]

MANTLGFRCLSLLGIVGALSGCSGAEPSGESPEATGQVESPAIVSSITEGDYVIRSAMTNKCIDVASSSTADGAKVQEWDCNGTNAQKFHISPTSDGYFKIINVNSGKGLDIQGVSTAQNALVHQWSYVGGANQQFRFVNRGGTQFSMQLRHTDMAVDLYWGSAENGTSLVQYPYTGGANQHWTFDLVSGGGGGTGFGGILTRDTFNAMFPSRNAFYSYDALVAAAAGFPGLATTGDTDTRKREVAAFLANVSHETGGLVYIEEINKADYCDTSWGPPGCFCAAGKRYYGRGPMQLSWNGNYCAAGNALGLPLQANPDLLAQDANAAWRTGFWFWTTQTGAGTMTAHNAIVNGAGFGETIRTINGSLECNGRNPGQVQSRIDAYVRFTGMLGVSPGNNLGC

>WP_120531197.1 MULTISPECIES: glycoside hydrolase family 19 protein [Corallococcus]

MANTLMFRCLSLMGMVGALGGCSGAEPSGEAPEALGQQESPAIVSSIVEGDYVIRSAMTNKCIDVASSSTADGAKVQEWDCNGTNAQKFHISPTSGGYFKIINVNSGKGLDVKDVSTAQNAIIHQWSYLGGNNQQFRFVNRGGTQFSMHLRHTDMAVDLSWGSADNGTPLLQYPYGNTANQHWTFDLVSGGGGGGTGFGGILTRDTFNAMFPSRNGFYTYDALIAAASGFPGLATTGDTDTRKREVAAFLANVNHETGGLVYIEEINKADYCDTSWGPPGCFCAPGKRYYGRGPMQLSWNGNYCAAGNALGLPLQANPDLLAQDANAAWRTGFWFWTTQNGAGSMTAHNAIVNGAGFGETIRTINGSRECNGANPGQVQSRIDAFVRFCGMLGVSPGNNLGC

>QAT82978.1 class I chitinase [Corallococcus coralloides]

MYESTSQEFLHLLAVLTGDVMSHKGMIRGLSLLGVVGALAGCSGPEAGDTAPETVGQVESAAVVSTITEGDYVIRSVRTNKCIDVASSSTADGAKVQQWDCNGTNAQKFRISPTSGGFWKIINVNSGKALDIQGVSTAENAVLHQWTYLDGANQQFQFINRGGTQFSFHPRHTGMAMDLSWGSFDNGTLIVQYPYYADRDNQRWTFDKVSGGGSGGSGFGGILTRDMFNTMFPSRNGFYTYDALIAAANGFPGLATTGDTDTRKREVAAFLANVSHETGGLVYIEEINKGDYCDTSWGPPGCSCAPGKRYFGRGPMQLSWNGNYCAAGNALGLPLQANPDLLAQDANAAWRTGFWFWTTQNGAGSMTAHNAIVNGAGFGETIRTINGSVECYGRNPGQVQSRIDKYLQFCSLLGVSPGNNLGC

>NNC09558.1 chitinase [Corallococcus exiguus]

GGGGGSGFGGILTRDTFNTMFPSRNGFYTYDALISAANSFPGLATTGDTDTRKREVAAFLANVSHETGGLVYIEEINKGDYCDTSWGPPGCSCAPGKRYFGRGPMQLSWNGNYCAAGNALGLPLQANPDLLAQDANAAWRTGFWFWTTQNGAGSMTAHNAIVNGAGFGETIRTINGSVECYGRNPGQVQSRIDKYLQFCSLLGVSPGTNLGC

>NOJ92413.1 chitinase [Corallococcus coralloides]

MSHKGMIRGLSLLGVVGALAGCSGPEAGDTAPETVGQVESAAVVSTITEGDYVIRSVRTNKCIDVASSSTADGAKVQQWDCNGTNAQKFRISPTSGGFWKIINVNSGKALDIQGVSTAENAVLHQWTYLDGANQQFQFINRGGTQFSFHPRHTGMAMDLSWGSFDNGTLIVQYPFYADRDNQRWTFDKVSGGGSGGSGFGAILTRDMFNTMFPSRNGFYTYDALIAAANGFPGLATTGDTDTRKREVAAFLANVSHETGGLVYIEEINKGDYCDTSWGPPGCSCAPGKRYFGRGPMQLSWNGNYCAAGNALGLPLQANPDLLAQDANAAWRTGFWFWTTQNGAGSMTAHNAIVNGAGFGETIRTINGSVECYGRNPGQVQSRIDKYLQFCSLLGVSPGNNLGC

>WP_128795205.1 glycoside hydrolase family 19 protein [Corallococcus coralloides]

MSHKGMIRGLSLLGVVGALAGCSGPEAGDTAPETVGQVESAAVVSTITEGDYVIRSVRTNKCIDVASSSTADGAKVQQWDCNGTNAQKFRISPTSGGFWKIINVNSGKALDIQGVSTAENAVLHQWTYLDGANQQFQFINRGGTQFSFHPRHTGMAMDLSWGSFDNGTLIVQYPYYADRDNQRWTFDKVSGGGSGGSGFGGILTRDMFNTMFPSRNGFYTYDALIAAANGFPGLATTGDTDTRKREVAAFLANVSHETGGLVYIEEINKGDYCDTSWGPPGCSCAPGKRYFGRGPMQLSWNGNYCAAGNALGLPLQANPDLLAQDANAAWRTGFWFWTTQNGAGSMTAHNAIVNGAGFGETIRTINGSVECYGRNPGQVQSRIDKYLQFCSLLGVSPGNNLGC

>RYZ38985.1 chitinase, partial [Myxococcaceae bacterium]

GGGTGFGGILTRDMFNAMFPSRNGFYTYDALIAAASGFPGLATTGDTDTRKREVAAFLANVNHETGGLVYIEEINKGDYCDPSWGPPGCSCAPNKRYYGRGPMQLSWNGNYCAAGNALGLPLQANPDLLAQDANAAWRTGFWFWTTQNGAGSMTAHNAIVNGAGFGQTIRTINGSVECDGRNPGQVQSRIDAFVRFCGMLGVSPGNNLGC

>WP_171434869.1 glycoside hydrolase family 19 protein [Corallococcus exercitus]

MSHKGIIRGLSLLGVVGVLGGCSGPEAEGNPPEATGQVESAAVVSTITEGDYVIRSVRTNKCIDVASSSTADGAKVQQWDCNGTNAQKFHISPTSGGFWKIINVNSGKALDIAGVSTAENAVLHQWTYVDGANQQFQFINRGGTQFSFHPRHTGMAMDLSWGSFDNGTQIVQYPYYADRDNQRWTFDLVSGGGSGGTGFAGILTRDMFNAMFPSRNAFYSYDALIAAANGFPGLATTGDTDTRKREVAAFLANVSHETGGLVYIEEINKADYCDTSWGPPGCSCAPGKRYYGRGPMQLSWNGNYCAAGNALGLPLQANPDLLAQDANAAWRTGFWFWTTQNGAGSMTAHNAIVNGAGFGETIRTINGSVECYGRNPAQVQSRIDKYLQFCSMLGVSPGANLGC

>WP_120608750.1 glycoside hydrolase family 19 protein [Corallococcus sp. CA053C]

MANTLGFRCLSLLGIVGALSGCSGAEPSGESPEATGQVEGAAVVSSITEGDYVIRSAMTNKCIDVASSSTADGAKVQEWDCNGTNAQKFHISPTSDGFFKIINVNSGKGLDIQGVSTAQNALVHQWSYVGGANQQFRFVNRGGTQFSMQLRHTDMAVDLYWGSAENGTSLVQYPYTGGANQHWTFDLVSGGGGTGFGGILTRETFNAMFPNRTAFYSYDALVAAAAGFPGLATTGDTDTRKREVAAFLANVSHETGGLVYIEEINKADYCDTSWGPPGCTCAAGKRYYGRGPMQLSWNGNYCAAGNALGLPLQANPDLLAQDANAAWRTGFWFWTTQTGAGSMTAHNAMVNGAGFGETIRTINGSLECNGRNPGQVQSRIDAYVRFTGMLGVSPGNNLGC

>WP_120618872.1 glycoside hydrolase family 19 protein [Corallococcus sp. CA049B]

MSHKGMIRGLSLLGVVGALAGCSGPEAGDTAPETVGQVESAAVVSTITEGDYVIRSVRTNKCIDVASSSTADGAKVQQWDCNGTNAQKFRISPTSGGFWKIINVNSGKALDIQGVSTAENAVLHQWTYLDGANQQFQFINRGGTQFSFHPRHTGMAMDLSWGSFDNGTLIVQYPYYADRDNQRWTFDKVSGGGSGGSGFGAILTRDMFNTMFPSRNGFYTYDALIAAANGFPGLATTGDSDTRKREVAAFLANVSHETGGLVYIEEINKGDYCDTSWGPPGCSCAPGKRYFGRGPMQLSWNGNYCAAGNALGLPLQANPDLLAQDANAAWRTGFWFWTTQNGAGSMTAHNAIVNGAGFGETIRTINGSVECYGRNPGQVQSRIDKYLQFCSLLGVSPGNNLGC

>WP_120524474.1 glycoside hydrolase family 19 protein [Corallococcus exercitus]

MNHKGMIRGLSLLGVVGALGGCSGPEAEGTPPEATGRVESAAVVSTITEGDYVIRSVRTNKCIDVASSSTADGAKVQQWDCNGTNAQKFHISPTSGGFWKIINVNSGKALDIAGVSTAENAVLHQWTYVDGANQQFQFINRGGTQFSFHPRHTGMAMDLSWGSFDNGTQIVQYPYYADRDNQRWTFDLVSGGGSGGTGFAGILTRDMFNAMFPSRNAFYSYDALIAAANGFPGLATTGDTDTRKREVAAFLANVSHETGGLVYIEEINKADYCDTSWGPPGCSCAPGKRYYGRGPMQLSWNGNYCAAGNALGLPLQANPDLLAQDANAAWRTGFWFWTTQNGAGSMTAHNAIVNGAGFGETIRTINGSVECYGRNPAQVQSRIDKYLQFCSMLGVSPGANLGC

>WP_267575772.1 glycoside hydrolase family 19 protein [Corallococcus sp. bb12-1]

MANTLMFRCLSLLGVVGALGGCSGEEPSGGAPETLGQQESAAIVSSIVEGDYVIRSAMTNKCIDVASSGTADGTKVQQWDCNGTNAQKFHISPTSGGYFKIINVNSGKGLDVVGVSTAANAVIHQWSYLGGNNQQFRFVNRGGTSFSMHLRHTDMAVDLSWGSPDNGTLLLQYPYGNTANQHWTFDLVSGGGGGGGTGFGGILTRETFNAMFPSRNGFYTYDALISAASGFPGLATTGDADTRKREVAAFLANVNHETGGLVYIEEINKADYCDTSWGPPGCSCAPGKRYFGRGPMQLSWNGNYCAAGNALGLPLQANPDLLSQDANAAWRTGFWFWTTQTGAGSMTAHNAIVNGAGFGQTIRTINGSRECNGANPGQVQSRIDAFVRFCGMLGVSPGNNLGC

>WP_206880513.1 glycoside hydrolase family 19 protein [Corallococcus exiguus]

MSHKGMIRGLSLLGVVGALAGCSGPEAGDTAQEAVGQVESAAVVSSITEGDYVIRSVRTNKCIDVASSSTADGAKVQQWDCNGTNAQKFHISPTSGGFWKIINVNSGKALDIVGVSTAENAVLHQWTYVDGANQQFQFINRGGTQFSFHPRHTGMAMDLSYGSPDNGTLIVQYPFYADRDNQRWTFDKVSGGGTGGGSGFGGILTRDMFNTMFPGRNGFYTYDALIAAANGFPGLATTGDTDTRKREVAAFLANVSHETGGLVYIEEINKGDYCDTSWGPPGCSCAPGKRYFGRGPMQLSWNGNYCAAGNALGLPLQSNPDLLAQDANAAWRTGFWFWTTQNGAGSMTAHNAIVNGAGFGETIRTINGSVECYGRNPGQVQSRIDKYVQFCSLLGVSPGNNLGC

>WP_169830916.1 glycoside hydrolase family 19 protein [Corallococcus exiguus]

MSHKGMIRGLSLLGVVGALAGCSGPEAGDTAQEAVGQVESAAVVSTITEGDYVIRSVRTNKCIDVASSSTADGAKVQQWDCNGTNAQKFHISPTSGGFWKIINVNSGKALDIVGVSTAENAVLHQWTYVDGANQQFQFINRGGVQFSFHPRHTGMAMDLSYGLPDNGTLIVQYPFYADRDNQRWTFDKVSGGGGGGGSGFGGILTRDTFNTMFPSRNGFYTYDALISAANSFPGLATTGDTDTRKREVAAFLANVSHETGGLVYIEEINKGDYCDTSWGPPGCSCAPGKRYFGRGPMQLSWNGNYCAAGNALGLPLQANPDLLAQDANAAWRTGFWFWTTQNGAGSMTAHNAIVNGAGFGETIRTINGSVECYGRNPGQVQSRIDKYLQFCSLLGVSPGTNLGC

>WP_043322686.1 glycoside hydrolase family 19 protein [Corallococcus coralloides]

MSHKGMFRGLSLLGVVGALAGCSGPEAGDTAPETVGQVESAAVVSTITEGDYVIRSVRTNKCIDVASSSTADGAKVQQWDCNGTNAQKFRISPTSGGFFKIINVNSGKALDIQGVSTAENAVLHQWTYVDGANQQFQFINRGGTQFSFHARHTGMAMDLSWGSFDNGTLIVQYPFYADRDNQRWTFDKVSGGGSGGGSGFGGILTRDMFNTMFPNRNGFYTYDALIAAANGFPGLATTGDTDTRKREVAAFLANVSHETGGLVHIEEINKGDYCDTSWGPPGCSCAPGKRYFGRGPMQLSWNGNYCAAGNALGLPLQANPDLLAQDANAAWRTGFWFWTTQNGAGSMTAHNAIVNGAGFGETIRTINGSVECYGRNPGQVQSRIDKYLQFCSLLGVSPGNNLGC

>AFE04038.1 class I chitinase [Corallococcus coralloides DSM 2259]

MYESTSQEFLHLLVVPTGDVMSHKGMFRGLSLLGVVGALAGCSGPEAGDTAPETVGQVESAAVVSTITEGDYVIRSVRTNKCIDVASSSTADGAKVQQWDCNGTNAQKFRISPTSGGFFKIINVNSGKALDIQGVSTAENAVLHQWTYVDGANQQFQFINRGGTQFSFHARHTGMAMDLSWGSFDNGTLIVQYPFYADRDNQRWTFDKVSGGGSGGGSGFGGILTRDMFNTMFPNRNGFYTYDALIAAANGFPGLATTGDTDTRKREVAAFLANVSHETGGLVHIEEINKGDYCDTSWGPPGCSCAPGKRYFGRGPMQLSWNGNYCAAGNALGLPLQANPDLLAQDANAAWRTGFWFWTTQNGAGSMTAHNAIVNGAGFGETIRTINGSVECYGRNPGQVQSRIDKYLQFCSLLGVSPGNNLGC

>WP_120587775.1 glycoside hydrolase family 19 protein [Corallococcus sp. CA054B]

MSHKGMFRGLSLLGVVGALAGCSGPEAGDTTPETVGQVESAAVVSTITEGDYVIRSVRTNKCIDVASSSTADGAKVQQWDCNGTNAQKFRISPTSGGFWKIINVNSGKALDIQGVSTAENAVLHQWTYVDGANQQFQFINRGGTQFSFHPRHTGMAMDLSWGSFDNGTLIVQYPFYADRDNQRWTFDKVSGGGSGGGSGFGGILTRDMFNTMFPNRNGFYTYDALIAAANGFPGLATTGDTDTRKREVAAFLANVSHETGGLVHIEEINKGDYCDTSWGPPGCSCAPGKRYFGRGPMQLSWNGNYCAAGNALGLPLQANPDLLAQDANAAWRTGFWFWTTQNGAGSMTAHNAIVNGAGFGETIRTINGSVECYGRNPGQVQSRIDKYLQFCSLLGVSPGNNLGC

>WP_126934498.1 glycoside hydrolase family 19 protein [Corallococcus sp. AB018]

MSHKGMIRGLSLLGVVGALAGCSGPEAGDTAQEAVGQVESAAVVSTITEGDYVIRSVRTNKCIDVASSSTADGAKVQQWDCNGTNAQKFHISPTSGGFWKIINVNSGKALDIVGVSTAENAVLHQWTYVDGANQQFQFINRGGVQFSFHPRHTGMAMDLSYGLPDNGTLIVQYPFYADRDNQRWTFDKVSGGGGGGGSGFGGILTRDMFNTMFPGRNGFYTYDALIAAANGFPGLATTGDTDTRKREVAAFLANVSHETGGLVYIEEINKGDYCDTSWGPPGCSCAPGKRYFGRGPMQLSWNGNYCAAGNALGLPLQSNPDLLAQDANAAWRTGFWFWTTQNGAGSMTAHNAIVNGAGFGETIRTINGSVECYGRNPGQVQSRIDKYQQFCSLLGVSPGTNLGC

>WP_120541618.1 glycoside hydrolase family 19 protein [Corallococcus terminator]

MANTLMFRCLSLLGVVGALGGCSGEPSGEAPETLGQQESPAIVSSIVEGDYVIRSAMTNKCIDVASSGTADGTKVQQWDCNGTNAQKFHISPTSGGYFKIINVNSGKGLDVQGVSTAPNAVIHQWSYVGGNNQQFRFVNRGGTSFSMHLRHTDMAVDLSWGSADNGTLLLQYPYGYTANQHWTFDLVSGGGGGGGGTGFGGILTRDTFNAMFPSRNGFYTYDALISAAGSFPGLATTGDADTRKREVAAFLANVNHETGGLVYIEEINKGDYCDTSWGPPGCSCAGGKRYYGRGPMQLSWNGNYCAAGNALGLPLQANPDLIAQDANVAWRTGFWFWTTQTGAGSMTAHNAIVNGAGFGQTIRTINGSLECDGRNPGQVQSRIDAYVRFCGMLGVSPGNNLGC

>WP_171413130.1 glycoside hydrolase family 19 protein [Corallococcus exercitus]

MSHKGMVRGLSLLGVVGVLAGCSGPEAGDAAPGAVGQVESAAVVSTITEGDYVIRSVRTNKCIDVASSSTADGAKVQQWDCNGTNAQKFHISPTSGGFWKIINVNSGKALDIAGVSTAENAVLHQWSYVGGANQQFQFINRGGTQFSFHVRHTGMAMDLSWGSFDNGTQIVQYPFYADRDNQRWTFDLVSGGGSGGTGFAGILTRDMFNAMFPSRNAFYSYDALIAAANGFPGLATTGDTDTRKREVAAFLANVSHETGGLVYIEEINKAPYCDTSWGPPGCSCAPGKQYYGRGPMQLSWNGNYCAAGNALGLPLQANPELLAQDANAAWRTGFWFWTTQTGAGSMTAHNAIVNGAGFGETIRTINGSVECYGRNPAQVQSRIDKYLQFCSMLGVSPGGNLGC

>WP_121778560.1 MULTISPECIES: glycoside hydrolase family 19 protein [Corallococcus]

MSHKGMIRGLSLLGVVGALAGCSGPEAGDTAQEAVGQVESAAVVSTITEGDYVIRSVRTNKCIDVASSSTADGAKVQQWDCNGTNAQKFHISPTSGGFWKIINVNSGKALDIVGVSTAENAVLHQWTYVDGANQQFQFINRGGVQFSFHPRHTGMAMDLSYGLPDNGTLIVQYPFYADRDNQRWTFDKVSGGGTGGGSGFGGILTRDMFNTMFPSRNGFYTYDALISAANSFPGLATTGDTDTRKREVAAFLANVSHETGGLVYIEEINKGDYCDTSWGPPGCSCAPGKRYFGRGPMQLSWNGNYCAAGNALGLPLQANPDLLAQDANAAWRTGFWFWTTQNGAGSMTAHNAIVNGAGFGETIRTINGSVECYGRNPGQVQSRIDKYLQFCSLLGVSPGTNLGC

>WP_120595231.1 MULTISPECIES: glycoside hydrolase family 19 protein [Corallococcus]

MSHKGMIRGLSLLGVVGALAGCSGPEAGDTAQEAVGQVESAAVVSTITEGDYVIRSVRTNKCIDVASSSTADGAKVQQWDCNGTNAQKFHISPTSGGFWKIINVNSGKALDIVGVSTAENAVLHQWTYVDGANQQFQFINRGGTQFSFHPRHTGMAMDLSYGSPDNGTLIVQYPFYADRDNQRWTFDKVSGGGTGGGSGFGGILTRDMFNTMFPGRNGFYTYDALIAAANGFPGLATTGDTDTRKREVAAFLANVSHETGGLVYIEEINKGDYCDTSWGPPGCSCAPGKRYFGRGPMQLSWNGNYCAAGNALGLPLQSNPDLLAQDANAAWRTGFWFWTTQNGAGSMTAHNAIVNGAGFGETIRTINGSVECYGRNPGQVQSRIDKYLQFCSLLGVSPGTNLGC

>WP_172816569.1 glycoside hydrolase family 19 protein [Corallococcus exiguus]

MSHKGMIRGLSLLGVVGALAGCSGPEAGDTAQEAVGQVESAAVVSTITEGDYVIRSVRTNKCIDVASSSTADGAKVQQWDCNGTNAQKFHISPTSGGFWKIINVNSGKALDIVGVSTAENAVLHQWTYVDGANQQFQFINRGGVQFSFHPRHTGMAMDLSYGLPDNGTLIVQYPFYADRDNQRWTFDKVSGGGGGGGSGFGGILTRDMFNTMFPGRNGFYTYDALIAAANGFPGLATTGDTDTRKREVAAFLANVSHETGGLVYIEEINKGDYCDTSWGPPGCSCAPGKRYFGRGPMQLSWNGNYCAAGNALGLPLQSNPDLLAQDANAAWRTGFWFWTTQNGAGSMTAHNAIVNGAGFGETIRTINGSVECYGRNPGQVQSRIDKYLQFCSLLGVSPGTNLGC

>WP_171806241.1 glycoside hydrolase family 19 protein [Corallococcus exiguus]

MSHKGMFRGLSLLGVVGALAGCSGPEAGDTAQEAVGQVESAAVVSTITEGDYVIRSVRTNKCIDVASSSTADGAKVQQWDCNGTNAQKFHISPTSGGFWKIINVNSGKALDIVGVSTAENAVLHQWTYVDGANQQFQFINRGGTQFSFHPRHTGMAMDLSYGSPDNGTLIVQYPFYADRDNQRWTFDKVSGGGTGGGSGFGGILTRDMFNTMFPGRNGFYTYDALIAAANGFPGLATTGDTDTRKREVAAFLANVSHETGGLVYIEEINKGDYCDTSWGPPGCSCAPGKRYFGRGPMQLSWNGNYCAAGNALGLPLQSNPDLLAQDANAAWRTGFWFWTTQNGAGSMTAHNAIVNGAGFGETIRTINGSVECYGRNPGQVQSRIDKYLQFCSLLGVSPGTNLGC

>WP_120555004.1 glycoside hydrolase family 19 protein [Corallococcus aberystwythensis]

MNHKGMIRGLSLLGVVGALGGCSGPDSVEGSPEATGQVESAAVVTTITEGDYVIRSVRTNKCIDVASSSTADGAKVQQWDCNGTNAQKFHISPTSGGFWKIINVNSGKALDIVGVSTAENAVLHQWSYVGGNNQQFQFINRGGTQFSFHPRHTGMAMDLSYGLPDNGTPIVQYPFYADRDNQRWTFDLVSGGGSGGGSGFAGILTRDMFNTMFPSRNGFYSYDALIAAANGFPGLATTGDTDTRKREVAAFLANVSHETGGLVYIEEINRGEYCDTSWGPPGCSCAPGKRYYGRGPMQLSWNGNYCAAGNALGLPLQANPDLLAQDANAAWRTAFWFWTTQNGAGSMTAHNAIVNGAGFGETIRTINGSVECYGRNPGQVQSRIDKYLQFCSLLGVSPGGNLGC

>WP_206799074.1 MULTISPECIES: glycoside hydrolase family 19 protein [unclassified Corallococcus]

MSHKGMIRGLSLLGVVGALAGCSGPEAEDTAPGAVGQVESAAVVSTITEGDYVIRSVRTNKCIDVASSSTADGAKVQQWDCNGTNAQKFHISPTSGGFWKIINVNSGKALDIVGVSTAENAVLHQWTYVDGANQQFQFINRGGTQFSFHPRHTGMAMDLSWGSFDNGTLIVQYPYYADRDNQRWTFDKVSGGGSGGTGFGGILTRDMFNTMFPGRNGFYTYDALIAAANTFPSVATTGDTDTRKREVAAFLANVSHETGGLVYIEEINKGDYCDTSWGPPGCSCAPGKRYFGRGPMQLSWNGNYCAAGNALGLPLQANPDLLAQDANAAWRTGFWFWTTQNGAGSMTAHNAIVNGAGFGETIRTINGSVECYGRNPGQVQSRIDKYLQFCSLLGVSPGNNLGC

>WP_223741138.1 glycoside hydrolase family 19 protein [Corallococcus sp. AS-1-12]

MSHKGIIRGLSLLGVVGALAGCSGPETGDTAQETVGQVESAAVVSTITEGDYVIRSVRTNKCIDVASSSTADGAKVQQWDCNGTNAQKFHISPTSGGFFKIINVNSGKALDIQGVSTAENAVLHQWTYVDGANQQFQFINRGGTQFSFHPRHTGMAMDLSWGSFDNGTQIVQYPYYPDRDNQRWTFDKVSGGGGGGTGFGGILTRDMFNTMFPSRNGFYTYDALIAAANTFPSVATTGDTDTRKREVAAFLANVSHETGGLVYIEEINKGDYCDTSWGPPGCFCAAGKRYYGRGPMQLSWNGNYCAAGNALGLPLQANPDLLAQDANAAWRTGFWFWTTQNGAGSMTAHNAIVNGAGFGETIRTINGSVECYGRNPGQVQSRIDKYLQFCSMLGVSPGNNLGC

>WP_223642882.1 glycoside hydrolase family 19 protein [Corallococcus sp. EGB]

MSHKGVIRGLSLLGVVGALAGCSGPETGDTAQAAVGQVESAAVVSTITEGDYVIRSVRTNKCIDVASSSTADGAKVQQWDCNGTNAQKFHISPTSGGFYKIINVNSGKALDIEGVSTAENAVLHQWTYVGGANQQFQFINRGGVQFSFHPRHTGMAMDLSWGSPDNGTLIVQYPFYPDRDNQRWTFDLVSGGGGGGGTGFAGILTRDTFNTMFPNRNGFYTYDALIAAANTFPGLATTGDTDTRKREVAAFLANASHETGGLVYIEEINQTSSYCDTSWGPPGCTCAPNKKYFGRGPMQLSWNGNYCAAGNALGLPLQANPELLAQDANASWRSGFWFWTTQTGAGSMTAHNAMVNGAGFGETIRTLNGSLECNGRNPAQVKSRVDAYKYFCSLLGVSPGANLEC

>WP_223765015.1 glycoside hydrolase family 19 protein [Corallococcus sp. AS-1-6]

MSHKGIIRGLSLLGVVGALAGCSGPETGDTAQETVGQVESAAVVSTITEGDYVIRSVRTNKCIDVASSSTADGAKVQQWDCNGTNAQKFHISPTSGGFFKIINVNSGKALDIQGVSTAENAVLHQWTYVDGANQQFQFINRGGTQFSFHPRHTGMAMDLSWGSFDNGTLIVQYPYYADRDNQRWTFDKVSGGGSGGTGFGGILTRETFNTMFPSRNGFYTYDALIAAANTFPSVATTGDTDTRKREVAAFLANVSHETGGLVYIEEINKGDYCDTSWGPPGCFCAAGKRYYGRGPMQLSWNGNYCAAGNALGLPLQANPDLLAQDANAAWRTGFWFWTTQNGAGSMTAHNAIVNGAGFGETIRTINGSVECYGRNPGQVQSRIDKYLQFCSMLGVSPGSNLGC

>WP_120625646.1 glycoside hydrolase family 19 protein [Corallococcus sicarius]

MANTLGFRCLSLLGIVGALSGCSGAEPSGESPEATGQVESPAIVSSITEGDYVIRSAMTNKCVDVASSSTADGAKVQEWDCNGTNAQKFHISPTSDGYFKIINVNSGKGLDIQGVSTAQNALVHQWSYVGGANQQFRFVNRGGTQFSMQLRHTDMAVDLYWGSAENGTSLVQYPYTGGANQHWTFDRVSGGGGGTGFGAILTRETFNAMFPNRTAFYSYDALIAAAAGFPGLATTGDTDTRKREVAAFLANVSHETGGLVYVEEINRGEYCDTSWGPPGCFCAAGKRYYGRGPIQLSWNGNYCAAGNALGLPLQANPDLLAQDANAAWRTGFWFWTTQTGAGSMTAHNAMVNGAGFGETIRTINGSVECYGRNPGQVQSRIDAYLRFTGMLGVSPGNNLGC

>WP_120547479.1 glycoside hydrolase family 19 protein [Corallococcus sp. AB049A]

MSHKGMIRGLSLLGAMGALAGCSGPEAGGDAPDTLSQVESAAVVSTITEGDYVIRSVRTNKCIDVASSSTADGAKVQQWDCNGTNAQKFHISPTSGGFWKIINVNSGKALDIQGVSTAENAVLHQWTYVDGANQQFQFINRGGTQFSFHPRHTGMAMDLSWGSFDNGTQIVQYPYYADRDNQRWTFDKVSGGGSGGTGFGAILTRDMFNTMFPGRNGFYTYDALIAAAATFPSVGTTGDTDTRKREVAAFLANVSHETGGLVYIEEINKGDYCDTSWGPPGCSCAPGKRYFGRGPMQLSWNGNYCAAGNALGLPLQANPDLLAQDANAAWRTGFWFWTTQNGAGSMTAHNAIVNNAGFGETIRTINGSVECYGRNPGQVQSRIDKYLQFCSMLGVSPGGNLGC

>WP_207051594.1 RICIN domain-containing protein [Corallococcus macrosporus]

MGHKGMIRGLSLLGVVGVLGGCSGPEAESTPPEATGQVESAAVVSTITEGDYVIRSVRTNKCIDVASSSTADGAKVQQWDCNGTNAQKFRISPTSGGFWKIINVNSGKALDIAGVSTAENAVLHQWTYVDGANQQFQFINRGGTQFSFHVRHTGMAMDLSWGSFDNGTQIVQYPFYADRDNQRWTFDKVSGGGTGGSGFGAILSRDLFNSMFPGRNGFYTYDALIAAANTFPGLATTGDTDTRKREVAAFLANVSHETGGLVYIEEINRGLYCDTSWGPPGCSCAPGKQYYGRGPMQLSWNGNYCAAGNALGLPLQANPDLLAQDANAAWRTGFWFWTTQTGAGSMTAHNAIVNGAGFGETIRTINGSVECYGRNPGQVQSRIDKYLQFCSMLGVAPGGNLGC

>WP_121769062.1 glycoside hydrolase family 19 protein [Corallococcus interemptor]

MSHKGMIRGLSLLGAMGALAGCSGPEAGGDAPDTLSQVESAAVVSTITEGDYVIRSVRTNKCIDVASSSTADGAKVQQWDCNGTNAQKFHISPTSGGFWKIINVNSGKALDIQGVSTAENAVLHQWTYVDGANQQFQFINRGGTQFSFHPRHTGMAMDLSWGSFDNGTQIVQYPYYADRDNQRWTFDKVSGGGSGGTGFGAILTRDMFNTMFPGRNGFYTYDALIAAAATFPSVGTTGDTDTRKREVAAFLANVSHETGGLVYIEEINKGDYCDTSWGPPGCSCAPGKRYFGRGPMQLSWNGNYCAAGNALGLPLQANPDLLAQDANAAWRTGFWFWTTQNGAGSMTAHNAIVNNAGFGETIRTINGSVECYGRNPGQVQSRIDNYLRFCSMLGVSPGGNLGC

>WP_193347200.1 MULTISPECIES: RICIN domain-containing protein [Corallococcus]

MANTSGFRCLSLLGIVGALGGCSGPEAPGESPEATGQVERPAIVSTITEGDYVIRSVMTNKCIDVASSSTADGAKVQQWDCNGTNAQKFHVAPTTNGYFKIINVNSGKGLDVQGVSTAQNAIIHQWSYVGGANQQFRFVNRGGTQFSMHLRHTDMAVDLSWGSADNGTPLLQYPYGGTANQHWSFDRVSGGGGGGGTGFGGILTRETFNAMFPNRHGFYTYDALVSAAASFSGLATTGDADTRKREVAAFLANASHETGGLVHIEEINKGDYCDTSWGPPGCFCAAGKRYFGRGPMQLSWNGNYCAAGIALGLPLQANPDLLAQDANAAWRAGFWFWTTQTGAGSMTAHNAIVNGAGFGETIRTINGSRECNGGNPGQVQSRIDTYVRFCGMLGVSPGNNLGC

>WP_047857615.1 RICIN domain-containing protein [Archangium gephyra]

MTRESLYRGLSLLGVVGALSGCALEAPESSEEISRTESAVIVSSITEGDYVIRSAMTQKCIDISASSTADGAKVQEWDCNGTNAQKFHLAPTSDGYWRIINVNSNKVLDIKDVSTAPNALVHQWSYVGGANQQFKFVARGNNQFSIHARHTDMAIDLYWGSADNGTILVQYPYSGGANQLWTFDNVGGGGTGGSGFGAILSRDMFNTMFPNRNPFYTYDALVAAASTFPGLATTGDTDTRKREVAAFLANVSHETGGLVHVEEINKAVYCDTSWGPPGCGCAAGKWYYGRGPMQLSWNGNYCAAGNALGLPLQSNPDLLAQDANASWRSGFWFWMTQTGAGTMTAHNAMVNNAGFGETIRTINGALECYGRNPGQVQSRIDTYLRFTSLLGVSPGSNLGC

>WP_073561246.1 glycoside hydrolase family 19 protein [Archangium sp. Cb G35]

MTREFVYRGLSLLGVVGALSGCALEAPESTEEISRTESAVIVSSITEGDYVIRSAMTQKCIDIASSSTADGAKVQQWDCNNTNAQKFRISPTSDGYWRIINVNSNKGLDIKEVSYAENAQVHQWAYLGGANQQFKFVPRGNNQFSIHPRHTDMALDLYWGSADNGTIIVQYPYTDRANQRWTFDKVGGGGGGGSGFGAILSRATFDTMFPNRNPFYTYDALVAAASTFPGLATTGDTDTRKREVAAFLANVSHETGGLVHIEEINKAAYCDTSWGPPGCSCAPGKWYYGRGPMQLSWNGNYCAAGNALGLPLQSNPDLLAQDANAAWRTGFWFWMTQTGAGNMTAHNAMVNNAGFGETIRTINGSLECYGRNPGQVQSRIDTYLRFTSLLGVNPGTNLGC

>WP_043410178.1 glycoside hydrolase family 19 protein [Archangium violaceum]

MTREFVYRGLSLLGVVGALSGCAMEASESTEEISRVESAAIVSSITEGDYVIRSAMTQKCIDISSSSTADGAKVQQWDCNNTNAQKFRISPTSDGYWRIINVNSNKGLDIKEVSYAENAQVHQWSYLGGANQQFKFVPRGNNQFSIHARHTDMALDLYWGSADNGTIIVQYPYSDRANQRWTFDKVDGGGSGGSGFGAILSRATFDTMFPNRNPFYTYDALVAAASTFPGLATTGDTDTRKREVAAFLANVSHETGGLVHIEEINKAAYCDTSWGPPGCSCAPGKWYYGRGPMQLSWNGNYCAAGNALGLPLQSNPDLLAQDANAAWRTGFWFWMTQTGAGNMTAHNAMVNNAGFGETIRTINGALECYGRNPGQVQSRIDTYLRFTGLLGVNPGSNLGC

>WP_267537219.1 glycoside hydrolase family 19 protein [Archangium lansinium]

MVRKSLYRSVSLLGVLGALSGCAVEELKSSEESAQTESAVIVSSITEGDYVIRSAMTQKCIDISSSSTADGAKVQEWDCNGTNAQKFHISPTSDGYWKIINVNSNKGLDIKEVSTAQNALVHQWTYGGGANQQFKFVARGNNQFSIHARHTDMAIDLYWGSAENGTILVQYPYTGGANQLWTFDKLGGSGATGLGAILSASMFNTMFPNRNPFYTYDALIAAASTFPGFATTGDTDTRKREVAAFLANVSHETGGLMYVEEINKAAYCDTSWGPPGCSCASGKWYYGRGPMQLSWNGNYCAAGNALGLPLQANPDLLAQDANASWRSGFWFWMTQTGAGSMTAHNAMVNNAGFGETIRTINGSLECYGRNPAQVQSRVDTYLRFTNLLGVSAGGNLGC

>WP_242515145.1 glycoside hydrolase family 19 protein [Sorangium cellulosum]

MSHQLGIIPGSFLSLAFIAAAVSASGCLVETSLDEEGFDSHEAAMCSSAPGWAEGVAYTIGSVVSYGGKSYSCRQSHTSLAGWTPAAVPALWQETGTCSGSSTSSSSSGGNGSGSGGNGSGCSYPAWQQGKNYRTGDIVIFNGSAYVAEHDNPGYDPTISTWFWDPHAGCGGSGSGSGSGGGGGGGGSGFGAILSESMFNTMFPARNPFYMYQALIAAALTFPGLATTGDADTRKREVAAFLANVSHETGGLVYVEEIAKAPLCDTSWGPPGCGCAPGKSYYGRGPMQLSWNGNYCAAGNALGLPLQSNPDLLAQDANAAWRSAFWFWTTQTGAGAMTAHDAIVTGAGFGETIRTINGALECNGRNPAQVQSRVNRYLQFTRLLGVSPGDHTGC

>WP_257452951.1 glycoside hydrolase family 19 protein [Archangium sp. CY-1]

MSRMSVYRCLSLLGVVGGLGGCAANELEGAEELAQLESAAIVSSITEGDYVIRSAMTNKCVDIASSSTADGAKVQQWDCNGTNAQKFHISPTSDGYWKIINVNSNKALDIKEVSTAQNALFHQWTYVGGNNQQFKFVARGNNQFSIHARHTDMAMDLYWGSANNGTEYVQYPYTGTANQLFTFDKAGGTPGNGLGAILSESTFNSMFPGRNPFYSYNALISAAATFPSFATTGDTDTRKREVAAFLANIAHETGNLVYIEEINKSVMCDTSWGPPGCGCAPGKMYYGRGPIQLSWNGNYCAAGNALGLNLMNDPDILSRDATASWRSGFWFWTTQTGAGSMTAHNAIVNGAGFGETIRTINGALECNGRNPAQVQSRIDNYLRFTSMLGVSPGSNLGC

>WP_108073745.1 glycoside hydrolase family 19 protein [Vitiosangium sp. GDMCC 1.1324]

MWRQGLCDISKRFLYSAMLFSSVAMVLASQEAAAACRGAWTEGSAYNVGDGVTYNGGSYTALVSHTACVGCGWNPVAAPSLWKTGGDCGSGGGGGGGGGGGGGTGLRAILSEATFNSMFPGRNGFYTYAALVAAADTFPGFATTGDTDTRKREVAAFLANISHETGGLVYTEEINKAPYCDTSWGPPGCSCASGKWYYGRGPMQLSWNGNYCAAGNALGVDLKNNPDLVATDATISWRTGFWFWMTQTGAGSMTAHNAMVNGAGFGQTIQTINGSLECGGRNPAQVQSRVNNYLNFTSKLGVSPGGNTGC

>WP_095983129.1 glycoside hydrolase family 19 protein [Melittangium boletus]

MASKTASRCLSLLGVLGGLSGCTVDEVKSVEPVDQIQREAIVSSISEGDYVIRSAQNNKCVDIAASGTADGTKVQLWDCNGTNAQKFAISPTSDGYWKIINVNSGKGFDIKEVSYAQNAELHQWSYVGGANQQFKFVNRGNNNFSIHARHTDMVIDLLWGSADNGTGFVQYPYTGTANQLYTLDKVSGGGTTPPPSGNGIAGILSESTFNAMFPGRNGFYSYSALVAAANTFSGFATTGDTDTRKREVAAFLANIAHETGNLVYIEEIAKSTMCDTSWGPPGCGCAAGKMYYGRGPIQLSWNGNYCAAGNALGVDLKNNPDLVARDATISWRTGFWFWMTQTGAGSMTAHNAIVNGAGFGETIRTINGALECGGRNPAQVQSRVNNYTRFCSLLGVSPGANTGC

>WP_203405794.1 glycoside hydrolase family 19 protein [Archangium violaceum]

MLRQSLSGLSKSILSAAMVFASMAMALASQEAAAACRGAWAEGSAYNAGDGVTYNGGSYTALVSHTACVGCGWNPVAAPSLWSQGGDCGGGGGGGGGGNGGGGDGGNGGTGLRAILSEATFNSMFPGRSSFYTYSALVAAADTFPGFATTGDLDTRKREVAAFLANIAHETGHLVYVEEINKSVMCDTSWGPPGCGCAPGKWYYGRGPIQLSWNGNYCAAGNALGVDLMNNPDLVARDATIAWRTGFWFWMTQTGAGSMTAHNAIVNGAGFGETIRTINGALECNGRNPAQVQSRVNNYLNFTSKLGVSPGGNTGC

>WP_108072736.1 glycoside hydrolase family 19 protein [Vitiosangium sp. GDMCC 1.1324]

MKAIGNRWVLLAGVGSMLLGAASCAPETAPASEAVGEVTQSLAAPIGQTIWLKACLNSKFISADGNLGATAPLVADRAAAAGWELFQVVDAGSGTIALRVSETGMYVSADTNLGAQLVANRTAIGDWERFTWVDFNNGTIGLIAKSTGKYVSTDANRGANAPLYADRATAGCWEAFSFGTSGSGGGGGGGGTGLGAILSESTFNAMFPGRNGFYTYAALVEAANTFPTFATTGDTDTRKREVAAFLANINHETGGLVYTEEINKAAYCDTSWGPPGCGCAPGKWYYGRGPIQLSWNGNYCAAGNYLGVDLKNNPELVATNATISWRTGFWFWMTQTGAGTMTAHNAMANGAGFGETIRTINGSLECGGRNPAQVQSRIDAYKRFCDMLGVSYGSNISC

>WP_095981465.1 glycoside hydrolase family 19 protein [Melittangium boletus]

MSRLTVFRCVSLLGALSGLGGCAVDEVKSVEPVDQLQREAIVSSITAGDYVIRSAQNNKCIDIASSGTADGTKVQLWDCNGTNAQKFAISATSDGYWKIINVNSGKGFDIKEVSYAQNAELHQWSYVGGANQQFKFVNRGNNNFSIHARHTDMVIDLLWGSANNGTGFVQYPYTGTANQLYTLDKVSGGGTTPPPSGNGIAGILSESTFNAMFPGRNGFYSYSALVAAANTFSGFATTGDTDTRKREVAAFLANIAHETGNLVYIEEIAKSTMCDTGWGPPGCGCAAGKMYYGRGPIQLSWNGNYCAAGNALGVDLKNNPDLVARDATISWRTGFWFWMTQTGAGSMTAHNAIVNGAGFGETIRTINGALECGGRNPAQVQSRVNNYTRFCSLLGVSPGANTGC

>WP_095976400.1 glycoside hydrolase family 19 protein [Melittangium boletus]

MKNFFYAATLVSSLAMGLVSQEAAAACRGAWTEGTTYSTGDGVTYNGGNYGALVGHTACVGCGWNPVAAPSLWQAGTGGCGGSGDGGNNPPNPPNPPTGNGIAGILSESTFNAMFPGRNGFYTYSALVAAANSFSGFATTGTTETRKREVAAFLANIAHETGNLVYIEEIAKSTMCDTSWGPPGCGCAAGKMYYGRGPIQLSWNGNYCAAGNALGVDLKNNPDLVARDATISWRTGFWFWMTQTGAGSMTGHNAIVNGAGFGETIRTINGALECGGRNPAQVQSRVNNYTRFCSLLGVSPGANTGC

>WP_204221668.1 RICIN domain-containing protein [Archangium violaceum]

MSRMSVYRCLSLLGVVGGLGGCAANELESAEETAQLRSAAIVSSITEGDYVIRSAMTNKCVDISSSSTADGAKVQQWDCNGTNAQKFHISPTSDGYWKIINVNSNKAIDVKDVSTAQNALFHQWTYVGGNNQQFKFVARGNNQFSIHVRHTDMVMDLYWGSANNGTEYVQYPYTGTANQLYTFDKVDGTTPGNGLGAILSESTFNSMFPGRNPFYSYSSLVAAAATFPSFATTGDTDTRKREVAAFLANVAHETGNLVYIEEINKSVMCDTSWGPPGCGCAPGKMYYGRGPIQLSWNGNYCAAGNALGLNLMNDPDILSRDATASWRSGLWFWNTQTGAGSMTAHNAMVNNAGFGETIRTINGALECNGRNPAQVQSRIDNYLRFTNMLGVSAGSNLGC

>MDC0707056.1 chitinase [Stigmatella ashevillena]

MTYNGGKYTARQAHTACVGCGWNPVAAPSLWQAGGDCTGGGDPPPPTGSGIAAILSESTFNTMFPGRNGFYTYSALVAAANTFPSFATSGDTATRKREVAAFLANISHETGGLVYIEEINKSVMCDTSWGPPGCGCAAGKWYYGRGPIQLSWNGNYCAAGNALGVDLKNNPDLVAQNATIAWRTGFWFWTTQTGAGSMTGHNAIVNGAGFGETIRTINGALECNGRNPAQVQSRINNYTRFLGLLGASAVGNNGC

>WP_307732460.1 glycoside hydrolase family 19 protein [Stigmatella ashevillena]

MAPQSSVFKEKFNMLRQELQGVSKSLLGAAVLFFSMAVVFAPQDAAAACRGAWAEGSAYNTGDGVTYNGGKYTARQAHTACVGCGWNPVAAPSLWQAGGDCTGGGDPPPPTGSGIAAILSESTFNTMFPGRNGFYTYSALVAAANTFPSFATSGDTATRKREVAAFLANISHETGGLVYIEEINKSVMCDTSWGPPGCGCAAGKWYYGRGPIQLSWNGNYCAAGNALGVDLKNNPDLVAQNATIAWRTGFWFWTTQTGAGSMTGHNAIVNGAGFGETIRTINGALECNGRNPAQVQSRINNYTRFLGLLGASAVGNNGC

>WP_187323609.1 glycoside hydrolase family 19 protein [Stigmatella aurantiaca]

MTYNGGTYTALQAHTACVGCGWNPAAVPSLWKAGGDCGSTPPPPPPPPPPPGGNGIAAILSESTFNTMFPSRNGFYTYAALVAAANTFPSFATSGDTATRKREVAAFLANISHETGGLVYIEEINKSVMCDTSWGPPGCGCAAGKWYYGRGPIQLSWNGNYCAAGNALGVDLKNNPDLVAQNATIAWRTGFWFWMTQTGAGSMTGHDAIVNGAGFGETIRTINGALECNGRNPAQVDSRVNNYNRFLGLLGASAVGNNRC

>WP_257460623.1 glycoside hydrolase family 19 protein [Archangium sp. CY-1]

MRPQGLCDISKKFMFAALVFSSTVTVLAPQVAEAACRGAWAEGTSYSTGDVVSYSGANYTALVGHTACVGCGWNPVAAPSLWSSGGNCDGGGGNNGGGGNNGGGGANPGVGGVLSEAMFNMMFPNRNPFYSFDAFFAAASTFPGFATTGDVDTRKREVAAFLANISHETGGLVYTEEINKSVMCDTSWGPPGCGCAPGKWYYGRGPIQLSWNGNYCAAGNALGVDLKNDPDLVARDAVIAWRTGLWFWMTQTGAGYMTGHNAIVNGAGFGETIRTINGALECNGRNPAQVQSRVNNYLNFTSMLGVNPGGNTGC

>EAU61742.1 chitinase [Stigmatella aurantiaca DW4/3-1]

MMWRQELRDVSKSLLGAAVLFFSMVVVFAPQDAAAACRGAWAEGSAYNVGDGVTYNGGTYTALQAHTACVGCGWNPAAVPSLWKAGGDCGSTPPPPPPPPPPPGGNGIAAILSESTFNTMFPSRNGFYTYAALVAAANTFPSFATSGDTATRKREVAAFLANISHETGGLVYIEEINKSVMCDTSWGPPGCGCAAGKWYYGRGPIQLSWNGNYCAAGNALGVDLKNNPDLVAQNATIAWRTGFWFWMTQTGAGSMTGHDAIVNGAGFGETIRTINGALECNGRNPAQVDSRVNNYNRFLGLLGASAVGNNRC

>ADO71774.1 Glycoside hydrolase, family 19 [Stigmatella aurantiaca DW4/3-1]

MALQPGSLREMFNMMWRQELRDVSKSLLGAAVLFFSMVVVFAPQDAAAACRGAWAEGSAYNVGDGVTYNGGTYTALQAHTACVGCGWNPAAVPSLWKAGGDCGSTPPPPPPPPPPPGGNGIAAILSESTFNTMFPSRNGFYTYAALVAAANTFPSFATSGDTATRKREVAAFLANISHETGGLVYIEEINKSVMCDTSWGPPGCGCAAGKWYYGRGPIQLSWNGNYCAAGNALGVDLKNNPDLVAQNATIAWRTGFWFWMTQTGAGSMTGHDAIVNGAGFGETIRTINGALECNGRNPAQVDSRVNNYNRFLGLLGASAVGNNRC

>WP_002617167.1 glycoside hydrolase family 19 protein [Stigmatella aurantiaca]

MSRMNLYRWLPLLGVMGGLGGCATPELETAEPLAQLQGEAIVSSITEGDYVIRSAMTNKCIDIASSNTADGAKVQQWDCNGTNAQKFHISPTSDGYWKIINVNSGKGLDIKEVSTAANAEVHQWSYVGGANQQFRFVGRGNNQFSIHVRHTDMAIDLYWGSADNGTIYVQYPYTGTSNQHYTFDKVDGGTQPPPTGTGIAAILSESTFNTMFPSRNGFYTYAALVAAANTFPSFATSGDTATRKREVAAFLANISHETGGLVYIEEINKSVMCDTSWGPPGCGCAAGKWYYGRGPIQLSWNGNYCAAGNALGVDLKNNPDLVAQNATIAWRTGFWFWMTQTGAGSMTGHDAIVNGAGFGETIRTINGALECNGRNPAQVDSRVNNYNRFLGLLGASAVGNNRC

>WP_272134251.1 glycoside hydrolase family 19 protein [Stigmatella ashevillena]

MSRMSLYRCLSLLGVMGGLGGCAVDEVETAGSIAQIQGEAIVSSITEGDYVIRSAMTNKCIDIASSNTADGAKVQQWDCNGTNAQKFHISPTSDGYWKIINVNSGKGLDIKEVSTAQNAEVHQWSYVGGANQQFKFVGRGNNQFSIHVRHTDMAIDLYWGSADNGTIYVQYPYTGAANQFYTFDKVDGTTPPPTGNGIAAILSESTFNTMFPGRNGFYTYSALVAAANTFPSFATSGDTATRKREVAAFLANISHETGGLVYIEEINKSVMCDTSWGPPGCGCAAGKWYYGRGPIQLSWNGNYCAAGNALGVDLMNNPDLVAQNATIAWRTGFWFWMTQTGAGSMTGHNAIVNGAGFGETIRTINGALECNGRNPAQVQSRINNYTRFLGLLGASAVGNNGC

>UQA63924.1 chitinase [Polyangium aurulentum]

MFNGAAYIAEHDNPGYDPTISTWFWDPYSGCTGSTGSGGSGGSGGSGGSGGSDPGTGLGAILSKSMFESMFPSRAPFYTYEALVAAAATFPAFATTGDLTTRKREVAAFLANVSHETGGLVYIEEIAKAPYCDTSWGPPGCSCAPGKWYYGRGPIQLSWNGNYCAAGNALGLPLQANPDLLAQDANAAWRTGFWFWTTQTGAGTMTPHNAMVTGAGFGETIRTINGALECNGGNPGQVQSRVNKYLEFTSKLGVTPGNNTGC

>UZH23237.1 hypothetical protein [myxobacterium MSr12020]

MTRKHSILPGSLLSLAFVAAATSAGGCLVDDAAEDDFESMELALCSAPAWAAGVAYTAGTVVNYGGKSYQCTQSHTSLAGWEPTAVPALWTETGTCSGSSSSSSSSSASSGSGGSGGAGSCNYPAWQQGKNYYTGNIVIFNGAPYIAEHDNPGYDPTISTWFWDPYPGCTSSGSGGSGGSGGSGGSGGSDPGTGLGAILSKSTFESMFPGRSPFYTYEALISAAATFPAFATTGDLTTRKREVAAFLANVSHETGGLVYVEEIAKAPYCDTSWGPPGCSCAAGKWYYGRGPIQLSWNGNYCAAGNALGLPLQANPDLLAQDANAAWRTGFWFWTTQTGAGTMTPHNAMVNGAGFGETIRSINGALECNGGNPAQVKSRVDKYLEFTGKLGVTPGNNTGC

>WP_044183154.1 RICIN domain-containing protein [Hyalangium minutum]

MSRMSMLRCISLLGVIGLGGCGANEMETLEPLSQLESAAIVSSLSEGDYVIRSAMTGKCVDVASSSTADGAKVQQWDCNGTNAQKFHISPTSDGFWKIINVNSGKGLDIKEVSTAQNAEVHQWSYLGGANQQFQFIARGNNQFSFHPRHTGMAIDLYWGSSNNGTIYVQYPYSGGPNQLFTLDKVSGGTTPPPTGTGLAAILSEATFNSMFPGRNGFYSYSALVAAASTFPAFATTGDTDTRKREVAAFLANVAHETGGLVYTEEINKSDMCDPSWGPPGCYCAAGKRYYGRGPIQLSWNGNYCAAGNALGLNLRDDPDLVARDANVAWRTGLWFWMTQTGAGSMTGHDAMVNNRGFGETIRTINGALECGGKNPGQVQSRVNNYLNFTGKLGVSAGANTGC

>WP_224249054.1 RICIN domain-containing protein [Cystobacter gracilis]

MSRMSMPRCVSLLAVIGLGGCAGDEWEAQEPLSQLESAAIVSSLSEGDYVIRSARTGKCVDVASSSTADGAKVQQWDCNGTNAQRFHISPTSDGFWKIINVNSGKALDIKGVSTAQNAELHQWSYVGGGNQQFQFIARGNNQFSFHPRHTGMAIDLYWGSADNGTIYVQYPYGGGDHQRFTLDKVGGTTPPPATGLASILSEATFNAMFPGRNGFYSYSALLAAASTFPAFATTGDTDTRKREVAAFLANVGHETGGLVHIEEINKSVMCDTSWGPPGCGCAPGKWYYGRGPIQLSWNGNYCAAGNALGVNLKDDPDLVARDAAIAWRTGLWFWMTQTGAGSMTAHDAMVNNRGFGETIRTINGAMECGGRNPGQVQSRVNNYLNFTGRLGVSAGGNTGC

>WP_082362387.1 glycoside hydrolase family 19 protein [Chondromyces crocatus]

MSRKLTIPGSLLSLLFAAAAVNAGGCVVDPSVDEDFEDFESQESAVCAGAPNWSENVAYGVGTVVQFGGKSYRCAQAHTSLAGWTPAAVPALWQETGTCSGSSSTTTSSSSSGGGTGSGGAGGGGGGSCNYPAWQQGRMYYPGDIVRFNGSLYIAEHENPGYDPTISTWFWDPYTCSGGTGGGGGGSGGSGGSGGGGNGGSGLAGVLSQNDFNAMFPNRNPFYTYNALVAAAATFPAFANTGSLETRKREVAAFLANVSHETGALVYIEEIQKGDYCDTSWGPPGCSCAPGKRYFGRGPIQLSWNGNYCAAGNALNLPLQSDPDLLSRDANAAWRTGLWFWTTQNGAGTMTAHSAMVNSRGFGETIRTINGALECNGRNPGQVQSRINTYQNFTNRLGVSPGNNLGC

>WP_255636364.1 RICIN domain-containing protein [Hyalangium versicolor]

MSMLRCVSLLGVVGLGGCAADEWEAQEQLSQVESAAVVSSISEGDYVIRSVMTGKCVDVASSSTADGAKVQQWDCNGTNAQKFHISPTSDGFWKIINVNSGKGLDIKEVSTAQNAQVHQWSYVGGGNQQFQFIARGNNQFSFHPRHTGMAIDLYWGSADNGTIYVQYPYTGGANQLFTFDKVSGGTTPPPATGLAGILSESTFNSMFPGRNGFYSYSALVAAANTFSAFATSGDTDTRKREVAAFLANVAHETGGLVYIEEINKSVMCDTSWGPPGCGCASGKWYYGRGPIQLSWNGNYCAAGNALGVNLKDDPDLVARDATIAWRTGLWFWMTQTGAGSMTAHDAMVNNRGFGETIRTINGALECGGRNPGQVQSRVNSYLDFTGRLGVSAGSNTGC

>WP_136924846.1 glycoside hydrolase family 19 protein [Polyangium aurulentum]

MSRNHSILRGSLLSLAFVAAAANAGGCIVEGSPEDDLDSAELALCSAPSWAEGVAYTVGTVVNYGGKSYQCQQNHTSLAGWTPAAVPALWLETGTCSGSTSSSSSSSSSASSGSGGAGGAGGSGSCNYPAWQQGKNYYTGNIVIFNGAAYIAEHDNPGYDPTISTWFWDPYSGCTGSTGSGGSGGSGGSGGSGGSDPGTGLGAILSKSMFESMFPSRAPFYTYEALVAAAATFPAFATTGDLTTRKREVAAFLANVSHETGGLVYIEEIAKAPYCDTSWGPPGCSCAPGKWYYGRGPIQLSWNGNYCAAGNALGLPLQANPDLLAQDANAAWRTGFWFWTTQTGAGTMTPHNAMVTGAGFGETIRTINGALECNGGNPGQVQSRVNKYLEFTSKLGVTPGNNTGC

>WP_095985405.1 glycoside hydrolase family 19 protein [Cystobacter fuscus]

MWRQGLRGFAKTFLSTAVVVSSLGMGLAAQDAAAACRGGWAEGTAYNVGDGVTYNGGKYSALVSHTACVGCGWNPVAAPSLWKTGGDCGTTTPPPTDPPPTGTGIAAILSESTFNSMFPGRNGFYTYSALVAAANTFPGFATTGDTDTRKREVAAFLANIAHETGGLVYIEEINKSVMCDTSWGPPGCGCAAGKMYYGRGPIQLSWNGNYCAAGNALGVDLKNDPDRVARDATIAWRTGLWFWMTQAGAGPRPAHDAIVNGFGFGGTIRSINGALECDGRNPAQVQSRVNSYLNFTSKLGVSPGGNTGC

>WP_095990964.1 glycoside hydrolase family 19 protein [Cystobacter fuscus]

MFYKPTYRGMALLGVLGGLSGCTVDEVASTEKLSQLESAAIVSSISEGDYVIRSVMTNKCIDVASSSTEDGAKVQQWDCNGTNAQKFHISPTSDGYFKIINVNSNKGLDIKEVSTAQNAQLHQWSYVGGGNQQFKFVGRGNSQFSMHVRHTDMAIDLYWGSADNGTQYVQYPYTGTANQLYTFDKVDGGTTPPPTGTGIAAILSEATFNSMFPGRNGFYTYSALVAAANTFPGFATTGDTDTRKREVAAFLANIAHETGGLVYIEEINKSVMCDTSWGPPGCGCAAGKMYYGRGPIQLSWNGNYCAAGNALGVDLKNDPDRVARDATIAWRTGLWFWMTQAGAGYRPAHDSMVNNLGFGETIRTINGALECNGKNPAQVQSRVNNYLNFTGKLGVNPGGNTGC

>WP_108074176.1 RICIN domain-containing protein [Vitiosangium sp. GDMCC 1.1324]

MSRMSMLRCVSLLGVIGLGGCAADEWEAQEPLSQLESAAIVSSLSEGDYVIRSVMTGKCVDVASSSTADGAKVQQWDCNGTNAQKFHISPTSDGFWKIINVNSGKGLDIKEVSTAQNAEFHQWSYVGGANQQFQFIARGNNQFSIHPRHTGMAMDLYWGSANNGTIYVQYPYEGRANQLFTLDKVGGTTPPPTGTGLAAILSEATFNSMFPGRNGFYSYSALVAAANTFPAFATTGNTDTRKREVAAFLANVSHETSGLVYIEEINKNVMCDTSWGPPGCGCAAGKWYYGRGPIQLSWNGNYCAAGNALGVNLKDDPDLVARNATIAWRTGLWFWMTQTGAGTMTAHNAMVNNQGFGETIRTINGALECGGRNPGQVQSRVNNYLNFTGRLGVSAGTNTGC

>WP_204226210.1 glycoside hydrolase family 19 protein [Archangium violaceum]

MRPQGLCDISKKFMFAALLFSSIATVLVPQIAAAACRGAWAEGTAYSTGDVVSYSGANYTALISHTACVGCGWNPVAAPSLWSTGGNCDGGGGNGGGGNGGGGTNPGVGGVLSEAMFNSMFPNRNPFYSFSAFFTAASTFPGFATTGDVDTRKREVAAFLANISHETGGLFYIEEINKSVMCDTSWGPPGCGCAPGKWYYGRGPIQLSWNGNYCAAGNALGLDLKNDPDLISRDAVVAWRTGLWFWMTQTGAGYMTGHDAMVNNVGFGETIRTINGALECNGRNPAQVQSRVNNYLDFTSRLGVNPGGNTGC

>OJH37211.1 chitinase [Cystobacter ferrugineus]

MASQHSTTEEMLDMRQGFCDISKKFLFAALLFSSMAMVLVPRAAEAACRGAWAEGTAYSAGDVVTYGGASYTALVAHTPCAGCGWNPVAAPSLWSTGGQCDGGGNGGGDGGGPASPGVGGVLSPAMFHNMFPGRNPFYSFESLFAAASTFPTFGTTGDVDTRKREVAAFLANVAHETGHLVYIEEINKSVMCDTSWGPPGCGCAPGKWYYGRGPIQLSWNGNYCAAGNALGLDLKNDPDLLSRDAVAAWRSALWFWMTQTGAGSRTGHDAIVSGAGFGETIRTINGALECNGRNPAQVQSRVNNYLRFTSMLGVSPGGNTGC

>WP_011554529.1 MULTISPECIES: RICIN domain-containing protein [Myxococcus]

MSGKFVSRSLALLGICGGLSALGGCGDAEPVPAALEPVGQVEGAAIVDTITEGTYVIRSVMTNKCIDVASSSTADGAKVQQWDCNGTNAQRFRVTPTSGGYFSIINVNSNKALDIKEASTAANALVHQWGYGGGANQQFRFVKEVGSEFSIRARHTDMAIDVYWGNTANGTELVQYPYEQRTNQRWTFDRIDGGGGNPGTGLAAILSESTFNAMFPNRNPFYTYSSLIAAASTFPAFANTGSLETRKREVAAFFANTAHETGNYVYVEEINRGDYCGSWGPPGCYCVAGKKYYGRGPIQLSWNGNYCAAGAALGLPLHTNPDLLAQDANASWRSAFWFWTTQAGAGTMSAHRAMVDGHGFGETIRTINGSLECNGRNPGQVQSRINNYQHFTNMLGVSPGGNLGC

>WP_245814616.1 glycoside hydrolase family 19 protein [Cystobacter ferrugineus]

MRQGFCDISKKFLFAALLFSSMAMVLVPRAAEAACRGAWAEGTAYSAGDVVTYGGASYTALVAHTPCAGCGWNPVAAPSLWSTGGQCDGGGNGGGDGGGPASPGVGGVLSPAMFHNMFPGRNPFYSFESLFAAASTFPTFGTTGDVDTRKREVAAFLANVAHETGHLVYIEEINKSVMCDTSWGPPGCGCAPGKWYYGRGPIQLSWNGNYCAAGNALGLDLKNDPDLLSRDAVAAWRSALWFWMTQTGAGSRTGHDAIVSGAGFGETIRTINGALECNGRNPAQVQSRVNNYLRFTSMLGVSPGGNTGC

>WP_204490605.1 glycoside hydrolase family 19 protein [Archangium primigenium]

MMGLKKLFSAAMMFSAMAMGFVAQDAAAACRGNWAEGTAYSVGDGVSYNGGKYTALQAHTACVGCGWNPAATPSLWKTGGDCSGGTTPPPTEPPPSTGGKGIAAFLSESQFNQMFPGRNGFYSYSALVAAANTFSGFATQGSTDNQKREVAAFLANVAHETGNLVYIEEIAKSTMCDTSWGPPGCGCAAGKQYYGRGPIQLSWNGNYCAAGNALGVDLKNDPDRVARDATIAWRTGFWFWMTQTGAGSMTAHNAMANGVGFGETIRTINGALECGGRNPAQVQSRINNYTRFCQILGVSVGSNTGC

>WP_176416701.1 MULTISPECIES: RICIN domain-containing protein [unclassified Myxococcus]

MSGKFVSRSLALLGICGGLSALGGCGGAEPAPAALEPVGQVEAAAIVDTITEGTYVIRSVMTSKCIDVASSSTADGAKVQQWDCNGTNAQRFRVTPTSGGYFSIINVNSNKALDIKEVSTAANALVHQWSYGGGANQQFRFVKEVGSEFSIRARHTDMAIDVYWGSTANGTELVQYPYEQRTNQRWTFDRVDGGGGNPGTGLAAILSESTFNAMFPNRNPFYTYSSLIAAANSFPAFANTGSLETRKREVAAFFANTAHETGHYVYVEEINRGDYCGSWGPPGCNCVAGKKYYGRGPIQLSWNGNYCAAGTALGLPLHTNPDLLAQDPNAAWRSAFWFWTTQAGAGTMSAHRAMVDGRGFGETIRTINGSLECNGGNPGQVQSRIDNYQRFTSMLGVSPGANLGC

>WP_074958445.1 RICIN domain-containing protein [Myxococcus fulvus]

MSGKFVSRSLALLGICGGLSALGGCDRAEPASADLEPVGQVESAAIVDTITAGTYVIRSVMTNKCIDVDSSSTADGAKVQQWDCNGTNAQRFVVTPTSGGYFSIINVNSNKALDIKEASVAANALVHQWSYVGGTNQQFRFVKEVGSEFSIRARHTDMAIDVYWGNTANGTELVQYPYEQRTNQRWTFDRIDGGGGTGLAAILSESTFNAMFPNRNPFYTYSSLIAAASTFPAFANTGSLETRKREVAAFFANTAHETGNYVYIEEINRGPYCGTWGPPGCNCVAGKQYYGRGPIQLSWNGNYCAAGAALNLPLHTNPDLLAQDANASWRSAFWFWTTQTGAGSMTAHSAMVNGAGFGETIRTINGTLECNGGNPGQVQSRIQKYQDIANLLGVSPGGNLGC

>WP_268781793.1 glycoside hydrolase family 19 protein [Myxococcus sp. NMCA1]

MSGKFVSRSLALLGICGGLSALGGCGDAEPAPAALEAVGQVEGAAIVDTITEGTYVIRSVMTNKCIDVASSSTADGAKVQQWDCNGTNAQRFRVTPTSGGYFSMINVNSNKALDIKEASTAANALVHQWGYGGGANQQFRFVKEVGSEFSIRARHTDMAIDVYWGNTANGTELVQYPYEQRTNQRWTFDRIDGGGGNPGTGLAAILSESTFNAMFPNRNPFYTYSSLIAAASTFPAFANTGSLETRKREVAAFFANTAHETGNYVYVEEINRGDYCGAWGPPGCYCVAGKKYYGRGPIQLSWNGNYCAAGTALGLPLHTNPDLLAQDANAAWRSAFWFWTTQAGAGTMSAHRAMVDGRGFGETIRTINGSLECNGGNPGQVQSRINNYQRFTGMLGVSPGDNLGC

>WP_253994529.1 RICIN domain-containing protein [Myxococcus qinghaiensis]

MSGKFVSRSLALLGICGGLSALGGCESAEPAPAALEPVGQVESAAIVDTITEGTYVIRSVMTNKCIDIDSSSTADGAKVQQWDCNGTNAQRFLVTPTSGGYFSIVNVNSNKGLDIKDGSTAANALVQQWGYGGGTNQQFRFVKEVGSEFSIRARHTDMAIDVYWGNTANGTELVQYPYEQRTNQRWTFDRIDGGGGTGLAAILSESTFNAMFPNRNPFYTYSSLIAAASTFPAFANTGSLETRKREVAAFFANTAHETGNYVYIEEIARGPYCGSWGPPGCNCAPGKQYYGRGPIQLSWNGNYCAAGAALNLPLHTNPDLLAQDANASWRSAFWFWTTQTGAGNMTAHSAMVNGHGFGETIRTINGTLECNGGNPGQVQSRIQKYQDIASMLGVSPGSNLGC

>WP_284667602.1 RICIN domain-containing protein [Myxococcus sp. SDU36]

MSGKFVSRSLALLGICGGLSALGGCGGAEPAPAALEPVGQVEAAAIVDTITEGTYVIRSVMTNKCIDVASSSTADGAKVQQWDCNGTNAQRFRVTPTSGGYFSIINVNSNKALDIKEVSTAANALVHQWSYGGGANQQFRFVKEVGSEFSIRARHTDMAIDVYWGSTANGTELVQYPYEQRTNQRWTFDRIDGGGGNPGTGLAAILSESTFNAMFPNRNPFYTYSSLIAAANSFPAFANTGSLETRKREVAAFFANTAHETGNYVYVEEINRGDYCGSWGPPGCNCVAGKKYYGRGPIQLSWNGNYCAAGTALGLPLHTNPDLLAQDPNAAWRSAFWFWTTQAGAGTMSAHRAMVDGRGFGETIRTINGALECNGGNPGQVQSRIDNYQRFTSMLGVSPGANLGC

>WP_140799005.1 RICIN domain-containing protein [Myxococcus xanthus]

MSGKFVSRSLALLGICGGLSALGGCGDAEPAPTALEPVGQVEGAAIVDTITEGSYVIRSVMTNKCIDVASSSTADGAKVQQWDCNGTNAQRFRVTPTSGGYFSIINVNSNKALDIKEASTAANALVHQWGYGGGANQQFRFVKEVGSEFSIRARHTDMAIDVYWGNTANGTELVQYPYEQRTNQRWTFDRIDGGGGNPGTGLAAILSESTFNAMFPNRNPFYTYSSLIAAASTFPAFANTGSLETRKREVAAFFANTAHETGNYVYVEEINRGDYCGAWGPPGCYCVAGKKYYGRGPIQLSWNGNYCAAGTALGLPLHTNPDLLAQDANAAWRSAFWFWTTQAGAGTMSAHRAMVDGRGFGETIRTINGSLECNGGNPGQVQSRINNYQRFTGMLGVSPGDNLGC

>WP_140855717.1 RICIN domain-containing protein [Myxococcus xanthus]

MSGKFVSRSLALLGICGGLSALGGCGDAEPAPAALEPVGQVEGAAIVDTITEGTYVIRSVMTNKCIDVASSSTADGAKVQQWDCNGTNAQRFRVTPTSGGYFSITNVNSNKALDIKEASTAANALVHQWGYGGGANQQFRFVKEVGSEFSIRARHTDMAIDVYWGNTANGTELVQYPYEQRTNQRWTFDRIDGGGGNPGTGLAAILSESTFNAMFPNRNPFYTYSSLIAAASTFPAFANTGSLETRKREVAAFFANTAHETGNYVYVEEINRGDYCGAWGPPGCYCVAGKKYYGRGPIQLSWNGNYCAAGTALGLPLHTNPDLLAQDANAAWRSAFWFWTTQAGAGTMSAHRAMVDGRGFGETIRTINGSLECNGGNPGQVQSRINNYQRFTGMLGVSPGDNLGC

>WP_140876036.1 RICIN domain-containing protein [Myxococcus xanthus]

MSGKFVSRSLALLGICGGLSALGGCGDAEPAPAALEPVGQVEGAAIVDTITEGTYVIRSVMTNKCIDVASSSTADGAKVQQWDCNGTNAQRFRVTPTSGGYFSIINVNSNKALDIKEASTAANALVHQWGYGGGANQQFRFVKEVGSEFSIRARHTDMAIDVYWGNTANGTELVQYPYEQRTNQRWTFDRIDGGGGNPGTGLAAILSESTFNAMFPNRNPFYTYSSLIAAASTFPAFANTGSLETRKREVAAFFANTAHETGNYVYVEEINRGDYCGAWGPPGCYCVAGKKYYGRGPIQLSWNGNYCAAGTALGLPLHTNPDLLAQDANAAWRSAFWFWTTQAGAGTMSAHRAMVDGRGFGETIRTINGSIECNGGNPGQVQSRINNYQRFTGMLGVSPGDNLGC

>WP_239013920.1 glycoside hydrolase family 19 protein [Archangium violaceum]

MRQGFCDISKKFLLAAMLFSSMTMVLVPQAAEAACRGAWAEGTAYSAGDVVTYGGATYTALVTHTPCAGCGWDPVAAPSLWSTGGNCDGGGNGNGGGGGGGASPGVGGVLSPGMFYNMFPDRNLFYSFESFFAAASTFPTFATTGDIDTRKREVAAFLANVAHETGHLVYVEEINKSVMCDTSWGPPGCGCAPGKWYYGRGPLQLSWNGNYCAAGNALGLDLMNDPDLLSRDAVAAWRSALWFWMTQTGAGSRTGHDAIVSGAGFGETIRTINGALECNGRNPAQVQSRVNNYLKFTSMLGVSPGANTGC

>QRK04340.1 chitinase [Archangium violaceum]

MTEENLDMRQGFCDISKKFLLAAMLFSSMTMVLVPQAAEAACRGAWAEGTAYSAGDVVTYGGATYTALVTHTPCAGCGWDPVAAPSLWSTGGNCDGGGNGNGGGGGGGASPGVGGVLSPGMFYNMFPDRNLFYSFESFFAAASTFPTFATTGDIDTRKREVAAFLANVAHETGHLVYVEEINKSVMCDTSWGPPGCGCAPGKWYYGRGPLQLSWNGNYCAAGNALGLDLMNDPDLLSRDAVAAWRSALWFWMTQTGAGSRTGHDAIVSGAGFGETIRTINGALECNGRNPAQVQSRVNNYLKFTSMLGVSPGANTGC

>WP_248544242.1 RICIN domain-containing protein [Myxococcus fulvus]

MSGHLVSRSLALLGICGGLSVLGGCERAEPASADLDPVGQVESAAIVDTITAGTYVIRSVMTNKCIDVASSSTADGAKVQQWDCNGTNAQRFVVTPTSGGYFSIINVNSNKALDIKEASVAANALVHQWSYGGGTNQQFRFVKEVGSEFSIRARHTDMAIDVYWGNTANGTELVQYPYEQRTNQRWTFDRVDGGGGTGLAAILSESTFNAMFPNRNPFYTYSSLIAAASTFPAFANTGSLETRKREVAAFFANTAHETGNYVYIEEIARGPYCGTWGPPGCNCVAGKQYYGRGPIQLSWNGNYCAAGAALNLPLHTNPDLLAQDANASWRSAFWFWTTQTGAGSMTAHSAMVNGAGFGETIRTINGALECNGGNPGQVQSRIQKYQDIANLLGVSPGGNLGC

>WP_267697367.1 RICIN domain-containing protein [Myxococcus sp. MISCRS1]

MSGHLVSRSLALLGICGGLSVLGGCERAEPASADLDPVGQVESAAIVDTITAGTYVIRSVMTNKCIDVASSSTADGAKVQQWDCNGTNAQRFVVTPTSGGYFSIINVNSNKALDIKEASVAANALVHQWSYGGGTNQQFRFVKEVGSEFSIRARHTDMAIDVYWGNTANGTELVQYPYEQRTNQRWTFDRVDGGGGTGLAAILSESTFNAMFPNRNPFYTYSSLIAAASTFPAFANTGSLETRKREVAAFFANTAHETGNYVYIEEIARGPYCGTWGPPGCNCVAGKQYYGRGPIQLSWNGNYCAAGAALNLPLHTNPDLLAQDANASWRSAFWFWTTQTGAGSMTAHSAMVNGAGFGETIRTINGTLECNGGNPGQVQSRIQKYQDIANLLGVSPGGNLGC

>WP_090494079.1 RICIN domain-containing protein [Myxococcus virescens]

MSGKFVSRSLALLGICGGLSALGGCGGAEPVPATLEPVGQVEGAAIVDTITEGTYVIRSVMTNKCIDVASSSTADGAKVQQWDCNGTNAQRFRVTPTSGGYFSIINVNSNKALDIKEVSTAPNARVHQWSYGGGANQQFRFVKEVGSEFSIRARHTDMAIDVYWGNTANGTELVQYPYEQRTNQRWTFDRIDGGGGNPGTGLAAILSESTFNAMFPNRNPFYTYSSLIAAANTFPAFANTGSLETRKREVAAFFANTAHETGNYVYVEEINRGDYCGSWGPPGCYCVAGKKYYGRGPIQLSWNGNYCAAGAALGLPLHTNPDLLAQDANAAWRSAFWFWTTQAGAGTMSAHRAMVDGHGFGETIRTINGSLECNGGNPGQVQSRINNYQHFTNMLGVSPGGNLGC

>AKF80321.1 chitinase [Myxococcus fulvus 124B02]

MSGNFVSRSLALLGICGGLSALGGCERAEPASADLEPVGQVESAAIVDTITAGTYVIRSVMTNKCIDVDSSSTADGAKVQQWDCNGTNAQRFVVTPTSGGYFSIINVNSNKALDIKEASVAANALVHQWSYGGGTNQQFRFVKEVGSEFSIRARHTDMAIDVYWGNTANGTELVQYPYEQRTNQRWTFDRVDGGGGTGLAAILSESTFNAMFPNRNPFYTYSSLIAAASTFPAFANTGSLETRKREVAAFFANTAHETGNYVYIEEIARGPYCGTWGPPGCNCVAGKQYYGRGPIQLSWNGNYCAAGAALNLPLHTNPDLLAQDANASWRSAFWFWTTQTGAGSMTAHSAMVNGAGFGETIRTINGTLECNGGNPGQVQSRIQKYQDIANLLGVSPGGNLGC

>WP_141619247.1 glycoside hydrolase family 19 protein [Myxococcus sp. AB036A]

MSGKFVSRSLALLGICGGLSALGGCGDAEPMPAALEPVGQVEGAAIVDTITEGTYVIRSVMTNKCIDVASSSTADGAKVQQWDCNGTNAQRFRVTPTSGGYFSIINVNSNKALDIKEVSTAPNALVHQWSYGGGANQQFRFVKEVGSEFSIRARHTDMAIDVYWGNTANGTELVQYPYEQRTNQRWTLDRIDGGGGNPGTGLAAILSESTFNAMFPNRNPFYTYSSLIAAANTFPAFANTGSLETRKREVAAFFANTAHETGNYVYVEEINRGDYCGSWGPPGCYCVAGKKYYGRGPIQLSWNGNYCAAGAALGLPLHTNPDLLAQDANAAWRSAFWFWTTQAGAGTMSAHRAMVDGYGFGETIRTINGSLECNGGNPGQVQSRINNYQHFTNMLGVSPGGNLGC

>WP_141592146.1 glycoside hydrolase family 19 protein [Myxococcus sp. AB056]

MSGKFVSRSLALLGICGGLSALGGCGDAEPVPAALEPVGQVEGAAIVDTITEGTYVIRSVMTNKCIDVASSSTADGAKVQQWDCNGTNAQRFRVTPTSGGYFSIINVNSNKALDIKEVSTAPNALVHQWSYGGGANQQFRFVKEVGSEFSIRARHTDMAIDVYWGNTANGTELVQYPYEQRTNQRWTLDRIDGGGGNPGTGLAAILSESTFNAMFPNRNPFYTYSSLIAAANTFPAFANTGSLETRKREVAAFFANTAHETGNYVYVEEINRGDYCGSWGPPGCYCVAGKKYYGRGPIQLSWNGNYCAAGAALGLPLHTNPDLLAQDANAAWRSAFWFWTTQAGAGTMSAHRAMVDGYGFGETIRTINGSLECNGGNPGQVQSRINNYQHFTNMLGVSPGGNLGC

>WP_223785466.1 RICIN domain-containing protein [Myxococcus sp. AS-1-15]

MSGHLASRSLALLGICGGLSVLGGCERAEPASADLDPVGQVESAAIVDTITAGTYVIRSVMTNKCIDVASSSTADGAKVQQWDCNGTNAQRFVVTPTSGGYFSIINVNSNKALDIKEASVAANALVHQWSYGGGTNQQFRFVKEVGSEFSIRARHTDMAIDVYWGNTANGTELVQYPYEQRTNQRWTFDRVDGGGGTGLAAILSESTFNAMFPNRNPFYTYSSLIAAASTFPAFANTGSLETRKREVAAFFANTAHETGNYVYIEEIARGPYCGTWGPPGCNCVAGKQYYGRGPIQLSWNGNYCAAGAALNLPLHTNPDLLAQDANASWRSAFWFWTTQTGAGSMTAHSAMVNGAGFGETIRTINGTLECNGGNPGQVQSRIQKYQDIANLLGVSPGGNLGC

>WP_163778223.1 RICIN domain-containing protein [Myxococcus vastator]

MSGKFVSRSLALLGICGGLSALGGCGGAEPAPAALEPVGQVEGAAIVDTVTEGTYVIRSVMTNKCIDVASSSTADGAKVQQWDCNGTNAQRFRVTPTSGGYFSIINVNSNKALDIKEVSTAANALVHQWSYGGGANQQFRFVKEVGSEFSIRARHTDMAIDVYWGNTANGTELVQYPYEQRTNQRWTFDRIDGGGGNPGTGLAAILSESTFNAMFPNRNPFYTYSSLIAAANTFPAFANTGSLETRKREVAAFFANTAHETGNYVYVEEINRGDYCGSWGPPGCNCVAGKRYYGRGPIQLSWNGNYCAAGTALGLPLHTNPDLLAQDPNAAWRSAFWFWTTQAGAGTMSAHRAMVDGRGFGETIRTINGALECNGGNPGQVQSRIDNYLRFTGMLGVSPGANLGC

>WP_204491464.1 glycoside hydrolase family 19 protein [Archangium primigenium]

MTSKIVSRGLSLVGVLAGLSGCAVEETKAPEQVAKLESAAIVSSVSAGTYVIRSAQNNKCLDIANSGTADGTKLQLWDCNGTNAQKFAVSATSDGYFKILNVNSNKAIDVKDVSTAQNAEIHQWSYVGGNNQQWKIVGRGNNQFSLHARHTDMVMDLLWGSANNGTGFVQYLYTGTANQLFTFDSTSGGTTPPPSGGKGISAYLSESQFNQMFPGRNGFYSYSALVAAANTFPDFATQGSVDNQKREVAAFLANVAHETGNLVYIEEIAKSTMCDTSWGPPGCGCAAGKQYYGRGPIQLSWNGNYCAAGNALGVDLKNDPDRVARDATIAWRTGFWFWMTQTGAGSMTAHNAMANGVGFGETIRTINGALECGGRNPGQVQSRIDNYNRFTQIIGVSAGGNTGC
